# A new 4-atom linker enables PROTAC development and imaging

**DOI:** 10.1039/d6cb00127k

**Published:** 2026-04-30

**Authors:** Spyros Letsios, Giovana Carrasco, Martin Lee, Mateen Wagiet, Marta Madureira, Marley Samways, Zaid Khan, Valerie G. Brunton, Olivera Grubisha, Alison N. Hulme

**Affiliations:** a EaStChem School of Chemistry, University of Edinburgh Edinburgh EH9 3FJ UK Alison.Hulme@ed.ac.uk; b Cancer Research UK Scotland Centre (Edinburgh), Institute of Genetics & Cancer, University of Edinburgh Edinburgh EH4 2XR UK; c UCB Slough UK

## Abstract

Proteolysis Targeting Chimeras (PROTACs) are heterobifunctional molecules, emerging as a promising class of drugs. Amongst their structural components, the linker moiety plays a pivotal role in modulating their biological activity and physicochemical properties. Current PROTAC design strategies involve utilising shorter and more rigid linkers to limit the number of possible conformations within the ternary complex. We hypothesised that employing a short diyne spacer as the linker between the two ligands could generate highly potent PROTACs. Here, we report a series of diyne-bearing, low nanomolar BRD4 degraders recruiting the CRL4^CRBN^ E3 ligase complex. As well as providing highly-active degraders, the Raman-active diyne moiety also enables the label-free visualisation of intracellular drug uptake at low micromolar concentrations *via* stimulated Raman scattering (SRS) microscopy. This work demonstrates the potential of diyne-based PROTAC linkers for both drug development and to address a key challenge within the field in understanding the cellular uptake mechanisms and intracellular localisation of PROTACs.

## Abstract 2

A rigid diyne linker gives a potent PROTAC that can be directly visualised in live cells by stimulated Raman scattering (SRS) microscopy.

## Graphical abstract

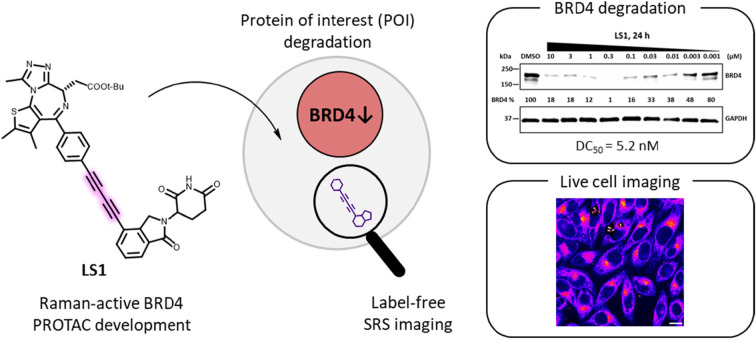

## Introduction

PROTACs are heterobifunctional molecules consisting of three components: a ligand binding to the protein of interest (POI), a ligand recruiting the E3 ubiquitin ligase, and a linker connecting the two. They recruit the ubiquitin proteasome system (UPS) by facilitating the formation of a ternary complex consisting of POI:PROTAC:E3 ligase, which leads to target protein ubiquitination and subsequent degradation *via* the proteasome.^1,2^ PROTACs offer many advantages over traditional small-molecule inhibitors, the most significant of which may be their catalytic mode of action. Because of this, PROTACs require only sub-stoichiometric intracellular concentrations to achieve target degradation.^3,4^ This property may help reduce overall toxicity and minimise off-target effects.^5^

Recent advances in the field have shown that the linker not only connects the POI and E3 ligase ligands, but also strongly influences degradation activity and overall PROTAC efficacy.^6^ By introducing modifications to the linker, such as altering the length, composition or rigidity, the physicochemical, pharmacokinetic (PK) and pharmacodynamic (PD) properties can be optimised.^6–9^ Notably, limiting the number of possible ternary complex conformations by using shorter linkers or increasing linker rigidity can enhance the stability of the ternary complex.^10^ These adjustments ease computational analysis by limiting the complex's degrees of freedom, and also help maintain a lower molecular weight.^6,10^ However, each protein system is unique, and developing a potent PROTAC can be a lengthy process that may require testing linkers of different length and rigidity. Therefore, advances in the field often referred to as “linkerology” are required to optimise PROTAC linker design.^9^ We sought to evaluate the use of a rigid 4-atom diyne to overcome some of the current limitations in PROTAC linker development.

**Fig. 1:**
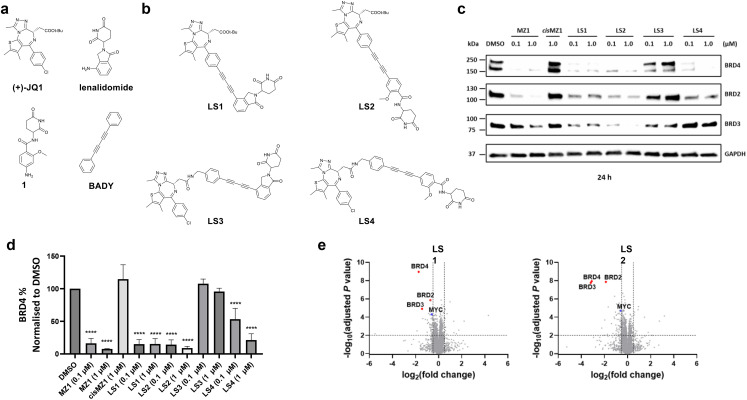
Designed Raman-active CRBN based PROTACs are potent BET degraders. (a) Chemical structures of BET bromodomain inhibitor (+)-JQ1, CRBN ligands lenalidomide and 1, and Raman-active probe BADY. (b) Designed Raman-active PROTACs LS1–LS4 bearing CRBN E3 ligase ligands. (c) Representative western blot after 24 h treatment of HeLa cells with DMSO, MZ1, *cis*MZ1, LS1, LS2, LS3 and LS4. (d) BRD4 quantification by western blot following the treatment conditions described in (c). Bars represent the mean ± SD from *n* = 3 biological replicates. Statistical analysis was performed using one-way ANOVA compared to DMSO; **** *p* < 0.0001. (e) Quantitative whole proteome analysis of HeLa cells after 6 h treatment with 30 nM of LS1 or LS2, compared to DMSO treatment (*n* = 4 biological replicates).

While significant progress has been made in PROTAC design strategies, high-resolution imaging of PROTACs that would yield useful insights on their intracellular localisation and mode of action remains underexplored. In recent years, stimulated Raman scattering (SRS) microscopy has emerged as a powerful new tool for label-free drug imaging.^11–15^ It offers valuable insights into the uptake and trafficking of (bio)molecules and aids selection of those with the most favourable drug-like properties. Among various functional groups, alkynes have been extensively employed as bioorthogonal Raman labels, generating signals within the cell-silent region of Raman spectra (1800–2800 cm^−1^).^16,17^ Advances in the design and development of alkyne-based Raman probes have significantly driven progress in this area.^18–20^ To date, the bisarylbutadiyne (BADY) moiety, which produces a strong signal at approximately 2200 cm^−1^, has been among the most successful Raman labels for drug imaging applications, due to its strong signal intensity and stability (Fig. 1a).^21,22^

In this study, we set out to design and synthesise a series of PROTACs that targeted the bromodomain and extra-terminal domain (BET) family proteins BRD2, BRD3, and BRD4. BRD4, in particular, has been one the most well investigated protein targets in PROTAC development.^23–25^ By coupling the aromatic rings of both the POI and E3 ligase ligands to a rigid 4-atom diyne linker, thus mimicking the BADY Raman probe, we sought to generate PROTACs that would produce an intense vibrational signal in the cell-silent region. By exploiting this feature, we planned to track the uptake of our PROTACs directly using SRS microscopy, and visualise their intracellular localisation at the low micromolar concentrations that are typical of drug screening assays, while confirming effective degradation activity under imaging conditions. If successful, this approach would provide the first high-resolution visualisation of PROTACs without the need to modify their structure to attach a label, offering new insights into PROTAC behaviour.

## Results and discussion

### Design and synthesis of diyne containing PROTACs

For the design of heterobifunctional BET degraders, the pan-BET inhibitor (+)-JQ1 was chosen as the ligand for the POI, as it has been extensively studied in PROTAC development (Fig. 1a).^23–28^(+)-JQ1 offers multiple exit vectors, including the *para*-Cl and ester moieties, suitable for attachment of the diyne spacers. Both exit vectors have been shown to accommodate rigid groups without significantly affecting POI binding.^26,29,30^ Cereblon (CRBN) protein was used to recruit the POI to an E3 ligase complex. Both lenalidomide and benzamide-type (1) CRBN binders were utilised to facilitate this recruitment, since these agents mimic the naturally occurring CRBN ligand (Fig. 1a).^31–33^

Two PROTACs were designed using the *para* position of the phenyl group of (+)-JQ1 as the exit vector. LS1 and LS2 (Fig. 1b) were obtained by linking the alkyne derivative of (+)-JQ1 to a bromo-alkyne derivative of lenalidomide (12) or of 1 (19) *via* Cadiot–Chodkiewicz coupling, with a yield of 63% and 33%, respectively for this key step (see Schemes S1–S4 for synthesis).^34^ To obtain PROTACs using the ester moiety as the exit vector (Fig. 1b), bisarylbutadiyne (BADY) derivatives of the CRBN ligands were synthesised with a methylamine handle at the *meta* and *para* positions respectively (Scheme S5). These CRBN binders were then coupled to the acid derivative of (+)-JQ1*via* amide bond formation to yield LS3 and LS4 in 47% and 59%, respectively (Scheme S6). Milligram quantities of LS1–LS4 were obtained for biological testing in ≥95% purity, as determined by LC-MS analysis (see SI for chromatograms).

### PROTACs bearing short diyne linkers are highly potent BET degraders

Due to the ability of (+)-JQ1 to bind the entire BET protein family, PROTACs LS1–LS4 were initially evaluated using western blot analysis to assess their degradation properties across the BET protein family members, BRD2, BRD3, and BRD4 (Fig. 1c). HeLa cells were treated with either 0.1 µM or 1.0 µM concentrations of compounds for 24 h. Treatments with the VHL-recruiting positive control BET PROTAC, MZ1, and the negative control, *cis*MZ1, were also conducted for comparison.^23^ Selective antibodies were used to probe for BRD2, BRD3, and BRD4. LS1 and LS2, which have the spacer element attached to the *para*-position of the phenyl moiety of (+)-JQ1, demonstrated notable BRD4 degradation at both concentrations, which was comparable to the effect of MZ1 (Fig. 1d). Interestingly, PROTACs with the linker at the ester moiety of (+)-JQ1 (LS3 and LS4) showed lower efficacy. BRD4 protein levels remained intact after LS3 treatment, while LS4 only effectively reduced BRD4 levels at 1.0 µM. Both LS1 and LS2 demonstrated partial degradation of BRD2 and BRD3, with LS2 showing the highest level of degradation against these proteins at 1.0 µM treatment.

To further characterise the most efficacious PROTACs, LS1 and LS2, and to assess whether they preferentially degrade BRD4, the prototypic member of the BET family, HeLa cells were treated with 30 nM of either LS1 or LS2 for 6 h, followed by quantitative whole-cell proteome analysis (Fig. 1e). Results indicated that LS1 most significantly downregulated BRD4, while LS2 equally downregulated BRD2, BRD3, and BRD4.

### Dose and time-dependent BRD4 degradation *via* the UPS

**Fig. 2:**
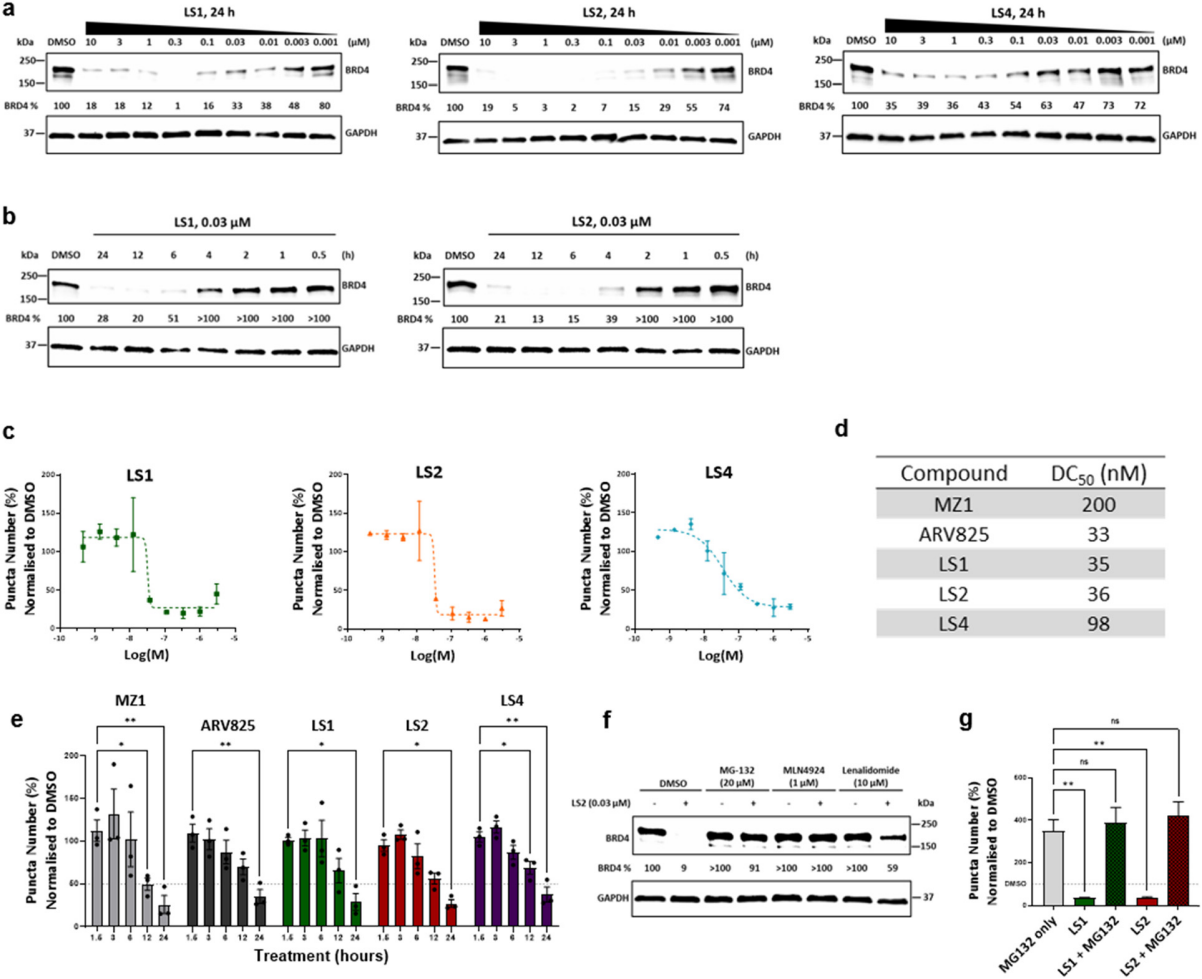
Characterisation of Raman-active BRD4 PROTACs. (a) Western blot analysis of BRD4 in HeLa cells treated with LS1, LS2 and LS4 BRD4 PROTACs at different concentrations for 24 h (*n* = 3 biological replicates). (b) Western blot analysis of BRD4 in HeLa cells treated with 0.03 µM of LS1 and LS2 at different timepoints (*n* = 3 biological replicates). (c) Immunofluorescence was performed to detect BRD4 puncta at different concentrations after treatment with BRD4 degraders for 24 h. Datapoints for BRD4 PROTACs were normalised to average of DMSO values to quantify the average BRD4 puncta per condition. Bars represent the mean ± SD from *n* = 2–3 biological replicates (d) half-maximal degradation concentrations (DC_50_) values calculated from the BRD4 puncta immunofluorescence assay. (e) Immunofluorescence was performed to detect BRD4 puncta at different time points after treatment with BRD4 PROTACs (concentrations: MZ1 300 nM, ARV825 150 nM, LS1 150 nM, LS2 150 nM, LS4 300 nM). BRD4 levels were normalised to respective time point DMSO values to quantify the average numbers of BRD4 puncta per condition per field of view. Statistical analysis performed using two-way ANOVA Dunnett's multiple comparison test, *n* = 3 biological replicates (ns non-significant, * *p* value < 0.05, ** *p* value < 0.01). (f) Western blot analysis of BRD4 in HeLa cells treated with 0.03 µM LS2 for 4 h. Cells were pre-treated for 2 h with DMSO, proteasome inhibitor MG132 (20 µM), neddylation inhibitor MLN4924 (1 µM), or lenalidomide (10 µM) (*n* = 3 biological replicates). (g) Immunofluorescence was performed to detect BRD4 puncta in HeLa cells after 12 h treatment with proteasome inhibitor MG132, and/or LS1 and LS2 (concentrations: MG132 20 µM, LS1 150 nM, LS2 150 nM). The average BRD4 puncta per condition were normalised to the average DMSO values and quantified for different treatments. Statistical analysis performed using two-way ANOVA Dunnett's multiple comparison test (*n* = 2 biological replicates, ns non-significant, ** *p* value < 0.01).

To further investigate the intracellular effects of the compounds, HeLa cells were treated with a range of concentrations of LS1, LS2 and LS4 for 24 h, and protein levels were quantified by immunoblotting. All compounds demonstrated BRD4 downregulation in a dose-dependent manner (Fig. 2a and Fig. S1a). Maximum degradation (>90%) was observed at 300 nM for LS1 and LS2, whereas LS4 only had a partial effect on BRD4, with a maximum degradation of approximately 65% at 300 nM (Fig. S1a). Interestingly, the level of BRD4 started to increase at concentrations of LS1 and LS2 above 1.0 µM and 3.0 µM, respectively. This phenomenon, commonly observed in three-component systems, is known as the hook effect, and arises from the formation of binary complexes at high PROTAC concentrations.^35,36^ To study the activities of LS1 and LS2 over time, HeLa cells were treated with 0.03 µM of each compound, and BRD4 levels were monitored by immunoblotting (Fig. 2b). Both compounds showed a progressive decrease in BRD4, with more than 80% degradation at 12 h (Fig. S2).

BRD4 forms puncta at super-enhancers, which are large nuclear phase-separated condensates that drive high levels of transcription.^37^ We used high content confocal imaging, to visualise nuclear BRD4 puncta and to determine the effect LS1 and LS2 PROTACs have on them.^38^ HeLa cells were treated with compounds, immunostained and BRD4 puncta were identified and counted as distinct fluorescent spots in the nucleus (Fig. S3). After 24 h treatment with a range of PROTAC concentrations, LS1 and LS2 demonstrated a reduction in the number of BRD4 puncta, with maximum efficacy achieved at ∼100 nM, and DC_50_ values of 35 nM and 36 nM, respectively. LS4 achieved a partial effect, and demonstrated a DC_50_ of 98 nM (Fig. 2c, d and Fig. S4a). Importantly, both LS1 and LS2 were over 5 times more potent than MZ1 (DC_50_ = 200 nM) and had comparable potency to CRBN recruiting BRD4 PROTAC ARV825 (DC_50_ = 33 nM) (Fig. 2d and Fig. S4b), underscoring the potential of diyne linkers in BRD4 PROTAC development.^24^ Time-dependent BRD4 degradation was confirmed using high-content imaging after treatment with 150 nM of ARV825, LS1, and LS2, or 300 nM of MZ1 and LS4 (Fig. 2e and Fig. S5). All PROTACs led to a decrease of BRD4 puncta, indicative of BRD4 degradation in cell nuclei over time, with more than a 50% decrease observed after 24 h. Interestingly, the rate of degradation observed in this assay was slower compared to the western blot analysis, a phenomenon previously observed.^38^

We next investigated the mechanism of BRD4 degradation by LS2. Treatment of HeLa cells with 0.03 µM LS2 for 4 h effectively degraded BRD4 (Fig. 2f). Pre-treatment with the proteasome inhibitor MG132 or the neddylation inhibitor MLN4924 completely rescued BRD4 from degradation by LS2 (Fig. 2f).^39^ Additionally, introducing an excess of CRBN ligand, lenalidomide, provided some protection against BRD4 degradation. This was also confirmed using the immunofluorescence assay where co-treatment with MG132 abolished degradation by LS1 or LS2 (150 nM) (Fig. 2g and Fig. S6). These data demonstrate that the PROTACs degrade BRD4 *via* the ubiquitin-proteasome system in a CRBN-dependent manner.

### Ternary complex modelling and surface plasmon resonance (SPR)

We then explored the molecular mechanism of LS1 and how its short, rigid linker can bring BRD4 and CRBN in close proximity. Using computational modelling, we predicted a ternary complex structure, benefiting from the known binding modes of (+)-JQ1 and lenalidomide ligands, to BRD4^BD1^ (PDB ID: 3mxf) and CRBN/DDB1 (PDB ID: 5fqd), respectively.^28,40^ Therefore, the only uncertain degree of freedom in LS1 is the dihedral angle of the diyne linker. This feature allowed for more predictable ternary complex structures compared to PROTACs with flexible linkers.^6^

**Fig. 3:**
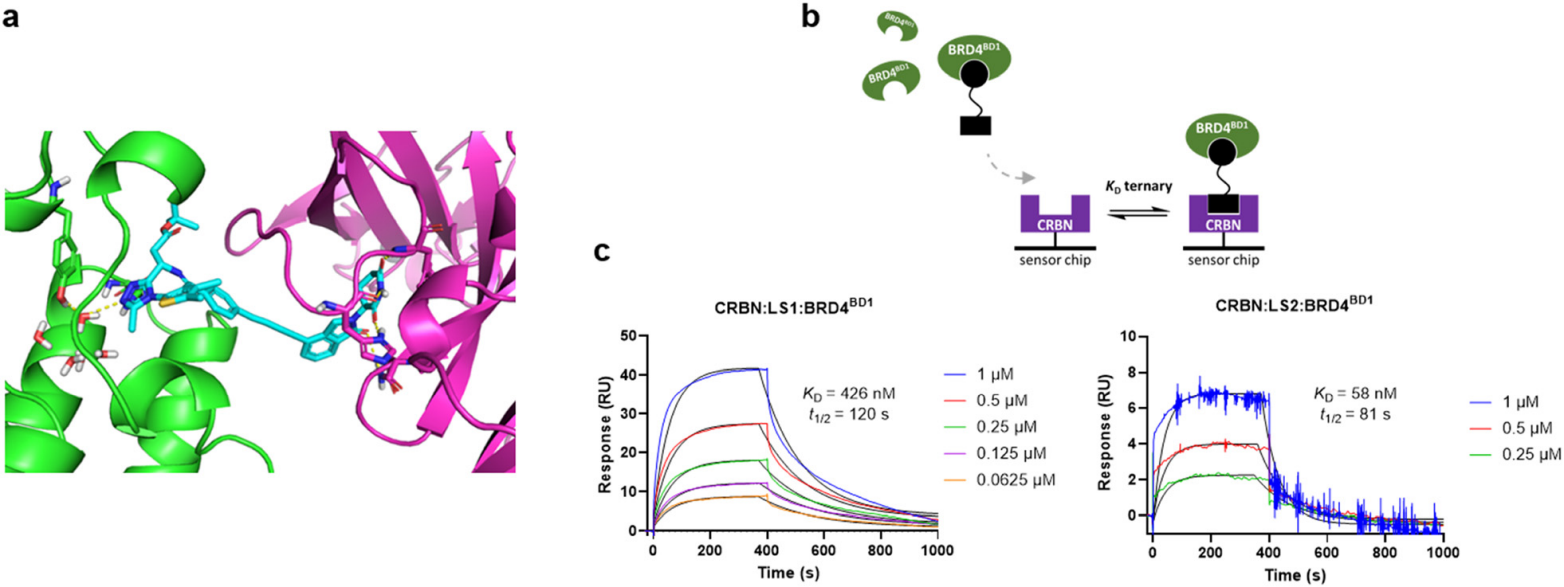
LS1 and LS2 form stable ternary complexes. (a) Modelled structure of the ternary complex formed between BRD4^BD1^ (green), LS1 (cyan) and CRBN (magenta). The structure shown is a representative snapshot taken from a 50 ns molecular dynamics simulation. (b) Schematic representation of the SPR binding assay used to measure the kinetics of ternary complex formation. (c) Representative SPR sensorgrams depicting the ternary complex binding kinetics for LS1:BRD4^BD1^ (left) and LS2:BRD4^BD1^ (right). (LS1: *n* = 2 independent experiments, LS2: *n* = 1).

36 ligand conformations were generated with equally spaced diyne dihedral angles. The BRD4 and CRBN proteins were then aligned onto each ligand conformer, rejecting any alignments which resulted in significant clashes between BRD4 and CRBN. Our analysis revealed that LS1's structural rigidity restricts BRD4-CRBN interfaces, with only 4 viable conformations out of 36. In contrast, LS2 which is less conformationally restricted, allowed for 23 conformations, highlighting the impact of structural restriction on protein alignment. Further refinement using molecular dynamics yielded a stable structure, with a representative snapshot of the BRD4(BD1):LS1:CRBN ternary complex, shown in Fig. 3a. These findings underscore the ability of diyne linkers to generate efficacious BRD4 PROTACs.

To monitor dissociation kinetics on ternary complexes we performed a Surface Plasmon Resonance (SPR) assay.^41^ When examining ternary complex formation within biophysical assays, a commonly used term is the “cooperativity” factor (*α*).^36^ This factor is defined as the ratio of binary over ternary dissociation constants (*α* = *K*_D_ binary/*K*_D_ ternary). When *α* > 1, cooperativity is positive, indicating enhanced ternary complex affinity. While not essential for PROTAC activity, gathering evidence suggests that stable ternary complexes with positive cooperativity can aid in PROTAC design.^41^

The SPR assay served to assess whether or not either LS1 or LS2 would create the appropriate multifaceted complex, consisting of BRD4^BD1^:PROTAC:CRBN, which is the required intermediate for ubiquitin transfer.^7^ Our assay involved pre-mixing the PROTAC with near-saturating concentrations of BRD4^BD1^ and then perfusing this complex solution to a pre-captured CRBN protein on the SPR chip surface (Fig. 3b). LS1 generated a *K*_D_ response of 426 nM with a half-life of *t*_1/2_ = 120 s, whilst in binary study format, the *K*_D_ for CRBN binding of LS1 was found to be 1320 nM (Fig. 3c, S7 and Table 1 and Table S1). Therefore, LS1 demonstrated positive cooperativity with an *α* value of 3.1. Further SPR studies confirmed that LS2 also facilitated ternary complex formation, with a *K*_D_ response of 58 nM and a half-life of *t*_1/2_ = 81 s. In binary assay format, LS2 produced a *K*_D_ of 1810 nM (Fig. 3c, Table 1 and Table S1), giving an *α* value of 31.1, indicating impressively positive cooperativity. These results suggest that the new diyne linker format adopted for both LS1 and LS2, despite its rigidity and short length, has the ability to form stable ternary complexes with promising dissociation kinetics. Positive control BRD4 PROTAC ARV825 having a flexible linker, was also utilised in this assay to provide comparison. It generated a *K*_D_ response of 188 nM with a half-life of *t*_1/2_ = 244 s, and an *α* value of 3.6 comparable to that of LS1 (Table 1).

**Table 1:** Binding kinetics of PROTAC:BRD4^BD1^ ternary complexes with immobilised CRBN measured by Surface Plasmon Resonance (SPR)^a^

Compound	*K*_D_ (nM)	*k*_on_ (M^−1^ s^−1^) × 10^5^	*k*_off_ (s^−1^)	*t*_1/2_ (s)	*α*
ARV825	188	0.21	0.0034	244	3.6
LS1	426	0.18	0.0084	120	3.1
LS2	58	1.47	0.0085	81	31.2

a For a detailed analysis of the binary complex results against BRD4^BD1^, BRD4^BD2^ and CRBN, see Table S1. SPR values were derived from fitted kinetic data: dissociation constant (*K*_D_ = *k*_off_/*k*_on_), and cooperativity (*α* = *K*_D_ binary (CRBN)/*K*_D_ ternary). (ARV825 & LS1: *n* = 2 independent experiments, LS2: *n* = 1).

### Label-free visualisation of LS1*via* SRS microscopy

**Fig. 4:**
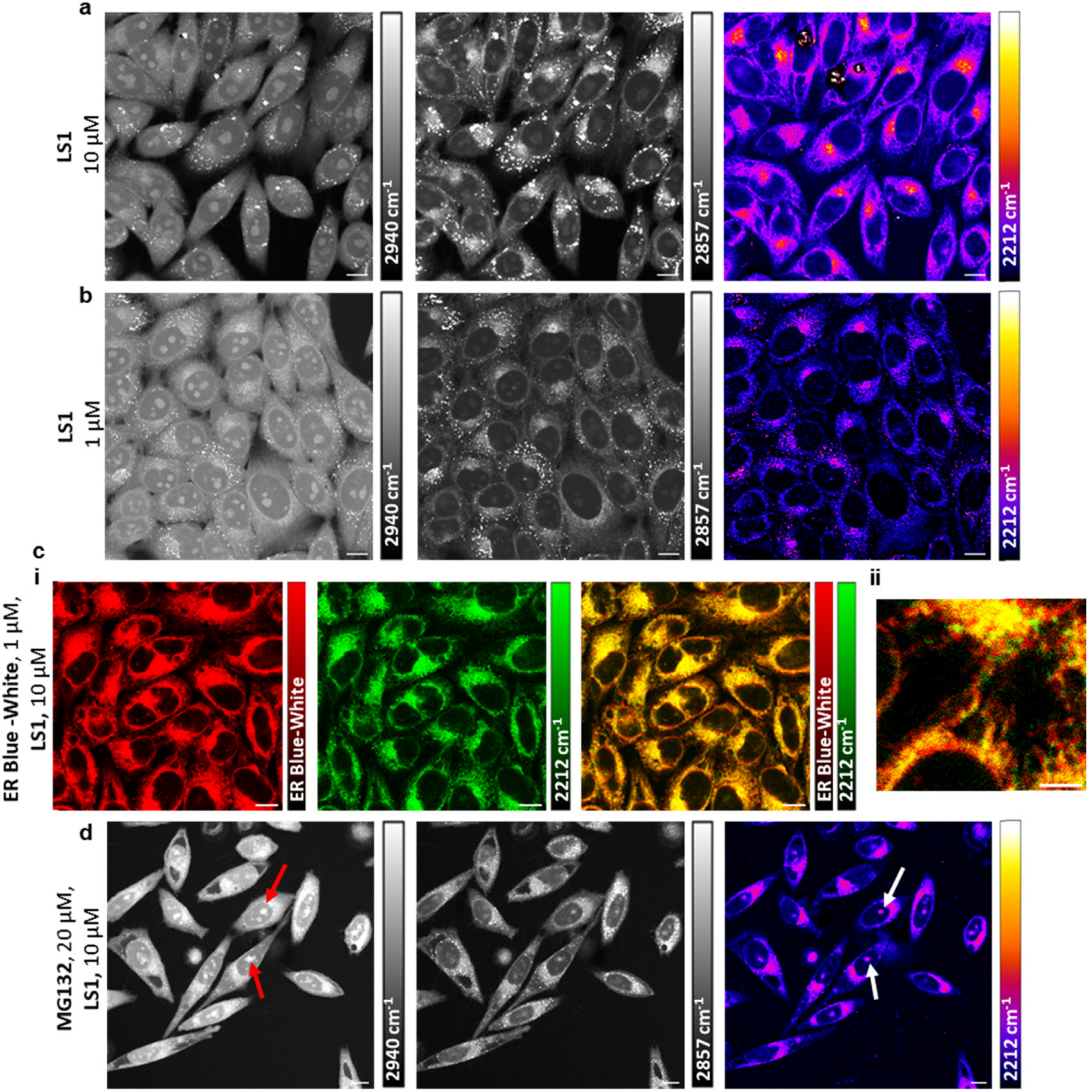
SRS imaging of live HeLa cells incubated with LS1. (a) 10 µM of LS1 for 1 h. (b) 1 µM of LS1 for 4 h. Images acquired at (L–R): 2940 cm^−1^ (CH_3_, proteins), 2844 cm^−1^ (CH_2_, lipids), 2212 cm^−1^ (C≡C, diyne). Alkyne images are background subtracted. Scale bar: 10 µm. (c) (i) 1 µM of ER tracker Blue-White for 30 min and 10 µM of LS1 for 4 h. Images acquired at (L–R): two photon fluorescence image (ER tracker Blue-White), 2212 cm^−1^ (C≡C, diyne), merge of images acquired. Scale bar: 10 µm. (ii) Zoomed image of the merged image from (i). Scale bar: 5 µm. (d) 20 µM of MG132 for 16 h and 10 µM of LS1 for 1 h. Images acquired at (L–R): 2940 cm^−1^ (CH_3_, proteins), 2844 cm^−1^ (CH_2_, lipids), 2212 cm^−1^ (C≡C, diyne). Red/white arrows indicate the nucleolar aggregates where LS1 is localised. Scale bar: 10 µm.

HeLa cells were incubated with LS1 (10 µM), and SRS imaging was employed to examine its intracellular localisation. By adjusting the energy difference between the pump and Stokes laser beams to be in resonance with cellular components such as proteins (CH_3_, 2940 cm^−1^) and lipids (CH_2_, 2857 cm^−1^), these components can be visualised directly (Fig. 4a). When tuned to 2212 cm^−1^, where the diyne linker resonates, LS1 was readily visible across the whole cell population after 1 h of treatment, with no changes in cellular morphology observed. Notably, LS1 visibly accumulated in a distinct area surrounding the nucleus (Fig. 4a). Cells treated with lower concentrations of LS1 (1 µM) demonstrated a similar intracellular localisation of drug compared to those of 10 µM treatments (Fig. 4b). These concentrations are typical of those used in initial drug screening assays and match or better the concentrations (3–25 µM) that have recently been used to directly visualise pomalidomide-bearing PROTACs using the intrinsic autofluorescence of this alkylamino-substituted phthalimide,^42,43^ while at the same time they offer much greater flexibility in PROTAC design.

To further study the intracellular localisation of LS1, we used multi-modal imaging. HeLa cells were simultaneously treated with LS1 (10 µM) and ER-tracker Blue-White (1 µM) to mark endoplasmic reticulum. Imaging showed that LS1 co-localised with ER-tracker (Fig. 4c, Pearson's coefficient: *r* = 0.83). For comparison, (+)-JQ1-*p*BADY and lenalidomide-BADY molecules were synthesised (see SI for synthesis). After SRS imaging both compounds demonstrated a similar localisation as LS1, mainly accumulating to the lipid rich region surrounding the nucleus (Fig. S8b, c). These findings align with previous imaging studies of (+)-JQ1 having amide-bound BADY and of 80S ribosome inhibitor anisomycin tagged with the BADY moiety.^21,22^ In contrast, when cells were incubated with the BADY probe (1 µM), it mainly accumulated within lipid droplets in the cytoplasm (Fig. S9). This indicates that the BADY probe itself does not direct the molecules toward the endoplasmic reticulum (ER). To provide comparison, the autofluorescence properties of the pomalidomide-bearing BRD4 PROTAC ARV825 were exploited to visualise its distribution within HeLa cells (Fig. S10a and b). Despite ARV825 containing a flexible PEG linker, its intracellular localisation was similar to that of LS1, and it was shown to be mainly localised within the ER of the cells (Fig. S10c). Taken together, these data suggest that our new linker moiety is not the driving factor behind the ER accumulation of our molecule. However, further studies will be required to fully elucidate these findings.

SRS microscopy offers the advantage that signal intensity is directly proportional to sample concentration.^44,45^ However, quantifying absolute intracellular concentrations with SRS can be challenging due to variations in signal intensity across cells.

We first demonstrated the linear relationship between LS1 concentration and Raman intensity through SRS imaging of LS1 in DMSO at various concentrations (Fig. S11a). By quantifying the intracellular concentration of LS1 in individual cells, we estimated the concentration range within cells (Fig. S11b). Quantification showed a stepwise increase in intracellular LS1, with 50- to 200-fold enrichment compared to treatment concentrations.

Although BRD4 is exclusively nuclear, LS1 predominantly localises to the endoplasmic reticulum (ER).^22,38^ Upon further examination, a weak nuclear LS1 signal was observed at a treatment concentration of 10 µM. A line profile of SRS intensity across a cell revealed a 3.5-fold increased nuclear intensity compared to background, while the cytoplasmic signal showed approximately a 5.5-fold increase in intensity (Fig. S12a and b). Additionally, hyperspectral imaging of the nuclear region revealed a weak on-resonance peak of LS1, further validating its presence within the nucleus (Fig. S12c and d). We also established that there was no unwanted degradation of the diyne vibrational motif in LS1 in the presence of intracellular nucleophiles such as glutathione (Fig. S13).^19,22^ Importantly, even after 4 h of treatment with 1 µM and 10 µM of LS1, more than 50% and 75% of BRD4 remained, respectively (Fig. S14). This indicates that the weak nuclear LS1 signal is not due to target protein degradation. Our previous results demonstrate that 10 µM and 1 µM concentrations of LS1 can efficiently degrade BRD4 after 24 h. Therefore, the nuclear levels of LS1 are sufficient to engage the target protein and degrade it *via* the UPS (Fig. 2a and c).

Intriguingly, proteasomal inhibition induces nucleolar aggregates containing ubiquitin and nucleoplasmic proteosome target proteins.^46^Fig. 2g shows that treatment with 20 µM MG132 for 12 h results in intranuclear BRD4 puncta approximately three times higher than in untreated cells. To enhance nuclear LS1 signal, we blocked the proteasome again with MG132 (20 µM) for 16 h before incubation with LS1. Notably, after proteasomal inhibition, LS1 localised not only in the ER but also within these nucleolar aggregates (Fig. 4d). Importantly, when BADY was added, it was not detected in the nucleolar aggregates (Fig. S15a), suggesting that the presence of LS1 is due to its binding to BRD4 and CRBN. Treatment with (+)-JQ1-*p*BADY and lenalidomide-BADY also showed a signal within the nucleolar aggregates, however, the signal intensity was much weaker compared to LS1 (Fig. S15b, c). A possible explanation is that both BRD4 and CRBN proteins are present in the aggregates, leading to higher quantities of LS1.

## Conclusions

PROTAC technology is an attractive approach to develop molecules for treating challenging diseases by targeting previously elusive proteins.^3^ Within this field, designing linkers with the ideal length, geometry and physicochemical properties remains a challenging area.^6,8^ In an effort to expand the linker toolbox for PROTAC development, we designed and synthesised CRBN-recruiting BET protein PROTACs using the pan-BET inhibitor (+)-JQ1, incorporating short, rigid diyne linkers.

Biological characterisation of our PROTACs, LS1–LS4, revealed that those having the linker attached at the *para* position of (+)-JQ1, LS1 and LS2, are particularly potent and rapid degraders of BRD4. LS1 and LS2 can achieve over 90% BRD4 degradation, and were shown to have DC_50_ values of 35 nM and 36 nM, respectively using a high-content confocal imaging assay. Mechanistic investigations confirmed that these PROTACs induce CRBN-dependent degradation *via* the UPS.

It has been previously reported that structurally constrained PROTAC linkers can enhance selectivity against BRD4 by limiting the number of accessible interprotein interactions.^27^ Interestingly, our quantitative whole-cell proteome analysis shows that despite the high conformational restriction of LS1 and LS2, both can significantly degrade BRD4, as well as BRD2 and BRD3. However, LS1 appears more selective for BRD4 compared to BRD2 and BRD3, suggesting that its higher conformational restriction results in increased selectivity. In contrast, LS2 shows no preference, which may be due to its more flexible structure.

SPR binding studies confirmed the formation of a stable, ternary complex, between BRD4^BD1^ and CRBN in the presence of LS1 or LS2. Notably, this complex exhibited positive cooperativity, with *α* values of 3.1 and 31.1, for LS1 and LS2, respectively. Understanding the cooperativity of PROTAC ternary complexes can provide crucial insights into their stability, potentially aiding in rational PROTAC design.^41^

Additionally, our computational model of the BRD4(BD1):LS1:CRBN ternary complex provided a structural hypothesis for ternary complex formation, while also demonstrating how the linker is predicted to be placed within the ternary complex. LS1 and LS2, to our knowledge, are the first known PROTACs with completely rigid and linear linkers. Their structural restriction provides an entropic advantage compared to flexible linker bearing PROTACs, allowing only rotational movement around the linker axis and significantly reducing potential conformations. Their structure together with our reported data make them attractive for *in silico* ternary complex structure predictions. Similar types of linkers could aid future drug design strategies by significantly reducing the uncertainty of linker protein interactions.

Utilising imaging technologies to directly track drugs can provide crucial information on intracellular drug localisation, identify mechanisms of drug resistance and guide modifications to improve cellular entry enhancing the success rate of drug candidates.^15^ It complements information provided by protein-tagging technologies that can track real-time degradation and recovery profiles for individual target proteins.^47^ Although, fluorescently labelled PROTACs have been utilised to demonstrate their mechanism of cellular uptake, the resolution provided by these is poor and the intracellular distribution of the PROTACs themselves remains largely unknown.^48^ Our SRS imaging data demonstrate that the label-free PROTAC LS1 can be visualised within cells at the low micromolar concentrations typically used in drug screening assays, and at which degradation activity has been confirmed. Unexpectedly, LS1 was observed mainly within the ER of the cells. Spectral analysis of SRS images revealed a weak nuclear signal for LS1, likely due to target engagement. Interestingly, ARV825, which lacks the rigid diyne moiety, was also co-localised within the ER of the cells, suggesting that the ER accumulation of LS1 is not driven by the linker's structure.

By exploiting the quantitative nature of SRS microscopy, we were able to measure the relative intracellular concentrations of LS1 within individual cells. After treatment with 1 µM of LS1, we observed approximately a 200-fold enrichment, however, at 10 µM, the enrichment decreased to 50-fold. Advancing this approach could be valuable for assessing drug permeability across cell membranes in the future.

Our findings demonstrate that a 4-atom, linear PROTAC linker, which cannot bend to fit the protein–protein interface, can be utilised to generate potent PROTACs while allowing for the formation of a stable ternary complex. These molecules are the first PROTACs, to our knowledge, that can be directly imaged using SRS microscopy in real time within live cells at biologically relevant concentrations and without the need for external probe attachment. This innovative approach not only enhances our understanding of PROTAC distribution but could also provide insights into drug localisation in resistant cell lines, as well as aid in studying the kinetics and mechanisms of uptake across various cell lines. We anticipate that future application of these linkers in PROTAC development will enable the targeting of novel proteins for both drug discovery and diagnostic purposes.

## Materials and methods

### Compounds

Detailed information of synthetic procedures for new compounds and intermediates can be found in Supplementary Methods. MZ1 and *cis*MZ1 were kindly provided by Boehringer Ingelheim *via* its open innovation platform opnMe, available at https://www.opnme.com/. (+)-JQ1 (T2110) used as a positive control was obtained from TargetMol. ARV-825 (21109) and MLN4924 (15217) were obtained from Cayman Chemical. **Lenalidomide** (SML2283) and MG132 (474790) were obtained from Sigma Aldrich/Merck. All compounds (≥95% purity, as determined by LC-MS analysis) were made up as 10 mM DMSO stock solutions. Laboratory areas in which teratogenic compounds were handled or stored were clearly labelled with appropriate hazard signage. All teratogenic compounds and contaminated materials were treated as hazardous and disposed accordingly.

### Mammalian cell culture

HeLa cells were cultured in DMEM (high glucose, GlutaMAX, Thermo Fischer Scientific), supplemented with 10% FBS (Thermo Fischer Scientific) and 1% Penicillin–Streptomycin (Thermo Fischer Scientific). All cells were maintained between 20–25 passages in 75 cm^2^ flasks and passaged with a confluency of 60–80%, using 0.05% Trypsin-EDTA (Sigma-Aldrich, No. T4174) and routinely tested for mycoplasma with a Mycoalert® Mycoplasma detection kit (Lonza, Little Chesterford, United Kingdom).

### Western blotting

HeLa cells (0.1 × 10^6^) were plated in 12-well plates. After 24 h, media was changed with compound-containing media. For competition assays, cells were pre-treated with 20 µM MG132, 1 µM of MLN4924 or 10 µM of lenalidomide for 2 h, before treating with LS2 at 30 nM for 4 h.

Cells were washed once with ice-cold PBS and lysed for 5 min at 4 °C with RIPA buffer (50 mM Tris-HCl, pH 7.4, 150 mM NaCl, 1% w/v Triton X-100, 0.5% w/v sodium deoxycholate, 0.1% w/v SDS; all from Sigma-Aldrich) supplemented with protease (0.4% v/v) and phosphatase (2% v/v) inhibitors (Sigma-Aldrich). After centrifugation (13 300*g* for 10 min at 4 °C) the protein concentration of the supernatant was quantified with Pierce™ BCA protein assay (23 227, Thermo Fischer Scientific) based on the manufacturer's instructions. Samples were prepared by 5 min incubation at 95 °C with NuPage™ LDS sample buffer (NP0007, Thermo Fisher Scientific). Proteins were separated according to size with Mini-Protean® TGX Precast gels with 20–30 µg protein per well and transferred to a PVDF membrane (Bio-Rad). The membrane was then blocked for 1 h with 5% milk TBS-T at room temperature, before incubating with primary antibodies at 4 °C overnight. The following primary antibodies were used: BRD2 (1 : 2000, Abcam, ab139690), BRD3 (1 : 200, Abcam, ab50818), BRD4 (1 : 1000, Thermo Fischer Scientific, A301-985A-T). Membranes were then washed with TBS-T three times and incubated for 1 h at room temperature with secondary anti-rabbit IgG HRP-linked antibody (1 : 3000, Cell Signalling Technology, 7074P2) or anti-mouse IgG HRP-linked antibody (1 : 3000, Cell Signalling Technology, 7076P2). After three TBS-T washes, the membrane was then incubated with Clarity Western ECL blotting substrate (Bio-Rad) or with SuperSignal™ West Femto Maximum Sensitivity Substrate (Thermo Fischer Scientific) and visualised using a ChemiDoc imaging system (Bio-Rad). All antibodies were diluted in 5% milk TBS-T.

### Immunofluorescence

HeLa cells (1 × 10^4^) were plated in 96-well imaging plates. After 24 h, cell growth media was replaced with 0.1% DMSO compound-containing media. For competition assay, cells were treated with 0.3% DMSO **MG132** and PROTAC containing media. Cells were fixed with 4% paraformaldehyde (PFA) for 15 min at room temperature. Cells were permeabilised using 10% (v/v) eBioscience™ Permeabilisation Buffer (10×, Thermo Fischer Scientific, 00-8333-56) in PBS for 15 min at room temperature and blocked using 10% (v/v) Blocker™ BSA (10×, Thermo Fischer Scientific, 37520) for 1 h at room temperature. Cells were then treated with primary anti-BRD4 (1 : 500, Abcam, ab128874) and incubated overnight at 4 °C. After washing three times with PBS, cells were incubated with fluorescent secondary antibody (Alexa Fluor 647, 1 : 1000, Thermo Fischer Scientific, A-21447), Alexa Fluor 568 phalloidin (1 : 10 000, Thermo Fischer Scientific, A12380) and NucBlue Fixed Cell ReadyProbes Reagent (2 drops per mL, Thermo Fischer Scientific, R37606) for 2 h at room temperature. All antibodies, phalloidin and NucBlue were diluted in blocking buffer. Images were acquired using Opera Phenix High Content Screening System (PerkinElmer). Images were processed and analysed using a pipeline to detect BRD4 puncta spots in live nuclei (Harmony v5.2).

### Whole-cell proteomics

HeLa cells (1 × 10^6^) were plated in 12-well plates. After 24 h, cells were treated in quadruplicate with 30 nM concentrations of LS1 and LS2, or with 0.1% DMSO. After 6 h, cells were washed three time with ice-cold PBS, and the cell pellets were isolated by centrifugation (300*g* for 4 min at 4 °C).

The pellet was lysed in 5% SDS, 100 mM Tris pH 8.5, 1 mg mL^−1^ chloroacetamide, 1.5 mg mL^−1^ Tris(2-carboxyethyl)phosphine. The lysate was sonicated using SoniPrep 150 (MSE) for 30 seconds and then heated at 95 °C for 30 min. The lysate was bound to MagReSyn® HILIC (Resyn Biosciences) beads and processed on KingFisher.^49^ Bound lysate was washed in acetonitrile followed by ethanol and the proteins were digested in 50 mM triethylammonium bicarbonate (TAEB) containing 1 µg trypsin. The resulting peptides were desalted using a C18 column.

The peptides were loaded onto 25 cm Aurora Columns (IonOptiks, Australia) and separated by nanoscale C18 reverse-phase liquid chromatography using an UltiMate 3000 RSLC nano system coupled online to an Orbitrap Fusion Lumos Tribrid mass spectrometer (Lumos) (all Thermo Fisher Scientific). HPLC buffers: 0.5% acetic acid in HPLC-grade water (buffer A); 0.5% acetic acid in HPLC-grade acetonitrile (buffer B) were prepared. HPLC method was programmed as follows: gradient increased from 5% to 30% buffer B in 70 min. The mass spectrometer was operated in DIA mode, acquiring a MS 350–1650 Da at 120k resolution followed by MS/MS on 45 windows with 0.5 Da overlap (200–2000 Da) at 30k with a NCE setting of 28.

### Molecular modelling

We adopted a “ligand-centric” approach to model the ternary complex structure formed between BRD4(BD1), LS1 and CRBN. This involved generating a series of LS1 conformers, where the dihedral angle in the linker was systematically explored in 10° increments (with the warhead conformations fixed). For each of these conformations, structures of BRD4(BD1) (PDB ID: 3mxf) and CRBN/DDB1 (PDB ID: 5fqd) were aligned by superimposing their respective ligands onto the PROTAC warheads (the protein structures were pre-prepared using the Schrödinger Protein Preparation Workflow).^28,40,50^ These structures were removed if they resulted in any clashes between protein backbone atoms, or greater than 20 total clashes between any heavy atoms (where a clash is defined as a heavy atom distance less than 2.4 Å).

Since the initial structures were generated by rigid-body alignment, we then relaxed the complexes. First, any protein sidechains involved in heavy atom clashes were removed and then re-modelled using the Schrödinger Protein Preparation Workflow.^50^ We then sought to use the OpenMM molecular dynamics (MD) software engine to further relax these complexes.^51^ In order to ensure a gradual relaxation for each structure, the solvated system was first minimised with harmonic restraints (10 kcal mol^−1^ Å^−2^ force constant) applied to all protein/ligand heavy atoms. The restraints were then removed on the protein sidechain atoms, and the system was re-minimised. This process was repeated to remove the restraints from the protein backbone atoms, and then finally from the ligand atoms. Each of these minimised structures was then simulated for 50 ns of MD, with structures saved every 0.5 ns (the first 10 ns of simulation was discarded as equilibration). Additional details around these MD simulations can be found in the Supporting Information. The structure shown in Fig. 3a was selected as a representative snapshot of these structural ensembles, as it showed the lowest median RMSD to all other simulation frames. This RMSD was calculated based on the BRD4 C_α_ atoms, after having aligned the CRBN backbone.

### SPR binding studies

SPR experiments were performed on Biacore T200 and BIAcore 1S+ instruments. Data generated was analysed in both: BIAcore T200 Evaluation Software (Version 3.0), and BIAcore Insight Evaluation Software (Version 6.0.7.1750). All interaction experiments were performed at 25 °C.

#### Immobilisation of BRD4^BD1^ and BRD4^BD2^

BRD4^BD1^ and BRD4^BD2^ (prepared in-house, see Table S2 for details) were amine-coupled on a Series S Sensor CM5 chip (Cat# 29149603) at 25 °C. The chip surface was first equilibrated with HBS-P+ (1×) (Cytiva, BR100671), followed by activation of the surface with EDC/NHS (Cytiva, BR100050) (contact time 420 s, flow rate 10 µL min^−1^). Thereafter, the sensor chip surface was equilibrated in running buffer (1% DMSO, HBS-P+ (1×)). BRD4^BD1^ and BRD4^BD2^ (1 µg mL^−1^, in running buffer), were then amine-coupled to the required surface density (100 RU).

#### Immobilisation of CRBN

GST-CRBN (Sino Biological, Cat# C55-30G) was captured at 25 °C on a Series S Sensor CM5 chip (Cat# 29149603), to which anti-GST antibody (Cytiva, BR100223) was immobilised following the GST Capture Kit protocol (BR100223). Briefly, the Sensor chip surface was equilibrated with HBS-P+ (1×) (Cytiva, BR100671), followed by activation of the surface with EDC/NHS (Cytiva, BR100671) (contact time 420 s, flow rate 10 µL min^−1^). The anti-GST antibody (30 µg mL^−1^, in running buffer), was then amine-coupled to the sensor chip surface, followed by deactivation using 1 M ethanolamine. Next, the sensor chip was equilibrated in running buffer (1% DMSO, HBS-P+ (1×)). GST tagged CRBN (1 µg mL^−1^, in running buffer) was then anti-GST antibody captured to the required density (100 RU).

#### Binary interaction experiments

Compounds (10 mM in 100% DMSO) were serially diluted in DMSO (10-point 2-fold dilutions, 50 mM to 98 µM). DMSO stock solutions were then diluted in HBS-P+ (1×), to make running buffer (1% DMSO, HBS-P+ (1×)) solutions (50 µM to 98 nM, 230 µL sample volume). Solutions were injected individually in multi-cycle kinetic format (contact time 120 s, flow rate 30 µL min^−1^, dissociation time 180 s) using a stabilisation period of 60 s and syringe wash (50% DMSO) between injections.

#### Ternary interaction experiments (immobilised CRBN)

Compounds (10 mM in 100% DMSO) were initially prepared at 2 µM in running buffer (1% DMSO, HBS-P+ (1×)). This solution was mixed 1 : 1 with a solution of 50 µM of BRD4^BD1^ or BRD4^BD2^ in running buffer, to prepare a final solution of 400 µL of 1 µM compound and 25 µM of bromodomain respectively. The complex was then serially diluted in running buffer containing 2 µM of bromodomain (4-point 2-fold serial dilution, 1 µM to 62.5 nM concentration of compound, and 25 µM to 3.44 µM concentration of bromodomain). Solutions were injected individually in multi-cycle kinetic format (contact time 400 s, flow rate 30 µL min^−1^, dissociation time 600 s) using a stabilisation period of 60 s and syringe wash (50% DMSO) between injections.

### Stimulated Raman scattering

For Imaging, supplemented phenol-red free DMEM (11054020, Gibco) was used. HeLa cells were plated in FluoroDish Cell Culture Dishes (35 mm, World Precision Instruments) at a density of 0.3 × 10^6^ cells per well in 2000 µL of cell suspension (70–80% confluency). The following day, cell growth media was replaced with compound-containing media diluted at the desired concentration (0.1% DMSO); cells were incubated for 1–4 h at 37 °C and imaged live.

Images were captured using a custom-designed multi-modal microscope setup. A picoEmerald S laser system (APE, Berlin, Germany) supplied a tunable pump laser (700–990 nm, 2 ps, 80 MHz repetition rate) alongside a spatially and temporally combined Stokes laser (1032 nm, 2 ps, 80 MHz repetition rate). The output beams were directed into the scanning unit of an Olympus FV1000MPE microscope through a series of dielectric mirrors and guided into an Olympus XLPL25XWMP N.A. 1.05 objective lens using a 690 nm short-pass dichroic mirror (Olympus). For SRS measurements, the Stokes beam was intensity modulated using a 20 MHz EoM integrated into the picoEmerald S. Forward scattered light was gathered with another 25× Olympus XLPL25XWMP N.A. 1.05 objective lens, with Stokes light being filtered out using a Chroma ET890/220 m filter. A telescope focused this light onto an APE silicon photodiode, linked to an APE lock-in amplifier set with a 20 µs time constant. The lock-in amplifier signal was transmitted to an Olympus FV10-Analog unit. Laser powers post-objective reached 20–100 mW for the pump laser and 50–400 mW for the Stokes laser. Images were recorded at resolutions of 512 × 512 or 1024 × 1024 pixels with a pixel dwell time ranging from 4 to 20 µs, by averaging 2–4 times, utilising Olympus's FluoView FV10-ASW scanning software. Image analysis and processing were carried out using ImageJ 1.53c.

To eliminate background processes in SRS images of alkynes, off-resonance images were captured by tuning the pump wavelength 2 nm (∼30 cm^−1^) away from the on-resonance image. These were then subtracted from the on-resonance image using ImageJ's ‘Image Calculator’ function. ImageJ also facilitated adjustments to brightness and contrast, as well as the assignment of false colours and scale bars.

### Statistical analysis

All statistical analyses and graphs were performed and generated using Prism version 9.3.0 (GraphPad, San Diego, CA, USA), and all data are presented as the mean ± SD. Statistical analysis for two groups of data from a single experiment were determined by unpaired *t*-test, differences between more than two groups with one or two independent variables were assessed by one-way or two-way ANOVA, respectively, followed by Tukey *post hoc* test with multiple comparisons between groups. *p*-Values to determine statistical significance are indicated in the text.

## Author contributions

SL conceptualisation, investigation, methodology, validation, writing – original draft & review. GC, ML, MW and MM investigation, methodology, validation, writing – review & editing. MS, and ZK investigation, methodology, writing – review & editing. VGB, OG and ANH conceptualisation, funding acquisition, supervision, writing – review & editing.

## Conflicts of interest

MW, MM, MS and OG are employees of UCB and may be shareholders.

## Supplementary Material

CB-007-D6CB00127K-s001

## Data Availability

The data supporting this article have been included as part of the supplementary information (SI). Supplementary information: Fig. S1–S18; MD simulation details; chemical and analytical methods for organic synthesis; experimental details and synthetic procedures for Schemes S1–S6; LC-MS traces & NMR spectra. See DOI: https://doi.org/10.1039/d6cb00127k.
