# Supplementary material for: A new 4-atom linker enables PROTAC development and imaging
Source: RSC Chem Biol. 2026 Apr 30. Online ahead of print. doi: 10.1039/d6cb00127k (PMC13141859; doi:10.1039/d6cb00127k)
Supplement: Supplementary file 1 [file CB-OLF-D6CB00127K-s001.pdf]

Supplementary Information (SI) for RSC Chemical Biology.
This journal is © The Royal Society of Chemistry 2026

Supplementary Information

A new 4-atom linker enables PROTAC development and imaging

Spyros Letsios,[ab] Giovana Carrasco,[b] Martin Lee,[b] Mateen Wagiet,[c] Marta Madureira,[c] Marley Samways,[c] Zaid Khan,[b] Valerie G. Brunton,[bd] Olivera Grubisha[cd] and Alison N. Hulme*[ad]

[a]EaStChem School of Chemistry, University of Edinburgh, David Brewster Road, Edinburgh, EH9 3FJ, UK.
E-mail: Alison.Hulme@ed.ac.uk
[b]Cancer Research UK Scotland Centre (Edinburgh), Institute of Genetics & Cancer, University of Edinburgh, Crewe Road South, Edinburgh, EH4 2XR, UK.
[c]UCB, Slough, UK
[d]These authors jointly supervised this work

Page **Contents**

S2 Figures S1 and S2: Quantification of BRD4 following Western blot analysis

S3 Figure S3: Pipeline analysis developed for high content imaging

S4 Figure S4: Immunofluorescence assay pipeline analysis for dose range experiment

S5 Figures S5 and S6: Representative immunofluorescence assay images demonstrating time-dependent and proteasomal-dependent degradation

S6 Figure S7 and Tables S1 and S2: Schematic representation of the binary complex SPR assay, binding kinetics data and list of proteins used in SPR assays

S7 Figure S8: SRS imaging of cells treated with DMSO or control compounds

S8 Figures S9 and S10: SRS imaging of cells treated with **BADY**, fluorescence imaging of **ARV825** treated cells and fluorescence imaging details

S9 Figures S11 and S12: Intracellular **LS1** concertation quantification and spectral information of live HeLa cells incubated with 10 μM **LS1**

S10 Figure S13 and S14: GSH Stability assessment of **LS1** and Western blot analysis at imaging conditions

S11 Figure S15: SRS imaging after proteasomal inhibition and incubation with control compounds

S12 Figures S16-S18: Uncropped Western blots

S13 MD simulation details

S14 Chemical and Analytical Methods

S15 Synthetic Procedures for Schemes S1-S6

S35 References

S36 LC-MS traces for compounds **LS1-4**

S40 ^{1}H and ^{13}C NMR spectra

a

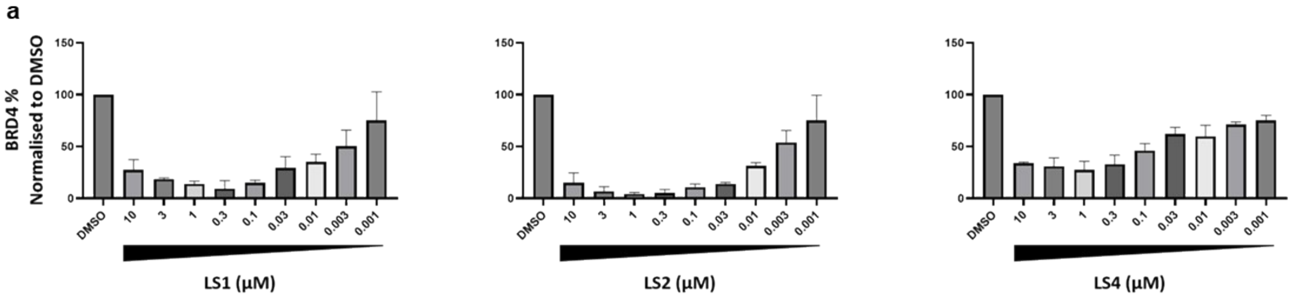

b

Compound	DC_{50} (nM)
LS1	5.2
LS2	5.2
LS4	57

Fig. S1| Quantification of BRD4 following Western blot analysis of HeLa cells treated for 24 h under specified conditions. (a) Percentage of BRD4 after treatment with serially diluted PROTACs **LS1**, **LS2**, and **LS4**, expressed relative to the DMSO control. Bars represent the mean ± SD from *n* = 3 biological replicates **(b)** Half-maximal degradation concentrations (DC_{50}) values derived from Western blot quantifications.

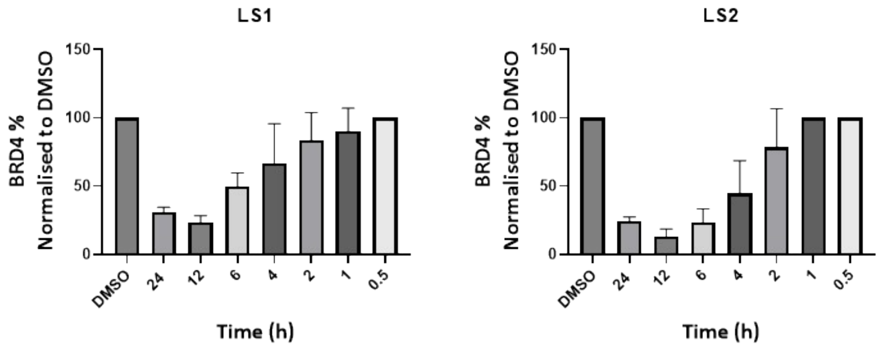

Fig. S2| Quantification of BRD4 following Western blot analysis of HeLa cells treated with 0.03 µM of LS1 or LS2 at different time points. BRD4 levels are expressed relative to the DMSO control. Bars represent the mean ± SD from *n* = 3 biological replicates.

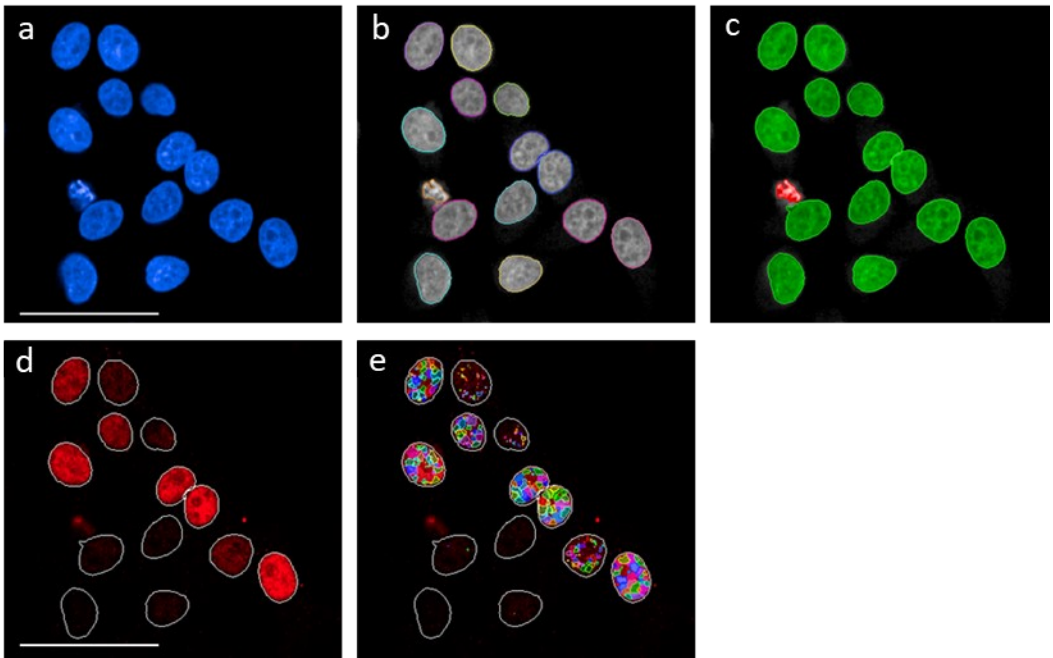

Fig. S3| Pipeline analysis developed for high-content confocal imaging. (a) Detection of cell nuclei using HOECHST staining (blue). (**b**) Detection of nuclei using a nuclei-specific mask. (**c**) Selection of live, non-dividing cells (green: selected nuclei, red: discarded nuclei). (**d**) Detection of BRD4 staining within selected nuclear regions (Red: Alexa Fluor 647) (**e**) Quantification of BRD4 condensates. Analysis was performed using Harmony v5.2. Scale bar: 50 µm.

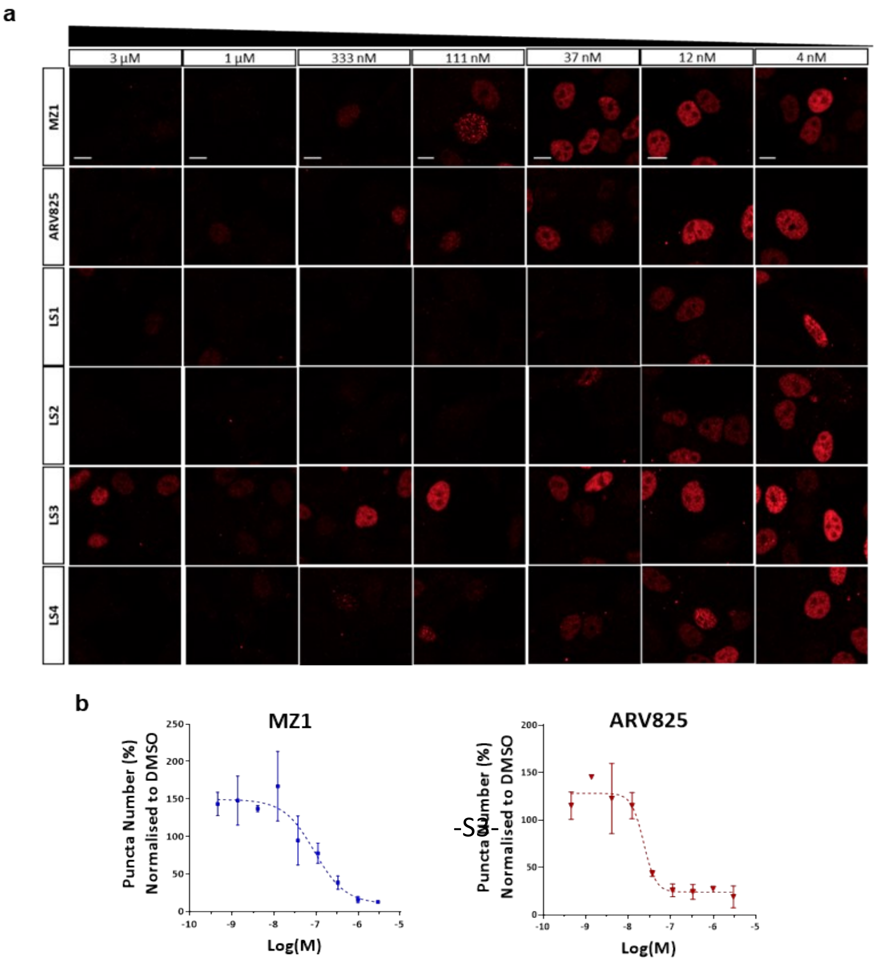

Fig. S4| Immunofluorescence assay pipeline analysis. (a) Representative images show nuclear BRD4 signals analysed after 24 h treatment with BRD4 PROTACs at various concentrations. DMSO at 0.1%. Scale bar: 10 µm. **(b)** HeLa cells were treated with a range of different concentrations of **MZ1** or **ARV825** for 24 h. Datapoints were normalised to average of DMSO values to quantify the average BRD4 condensates per field of view per condition. Bars represent the mean ± SD from *n* = 3 biological replicates.

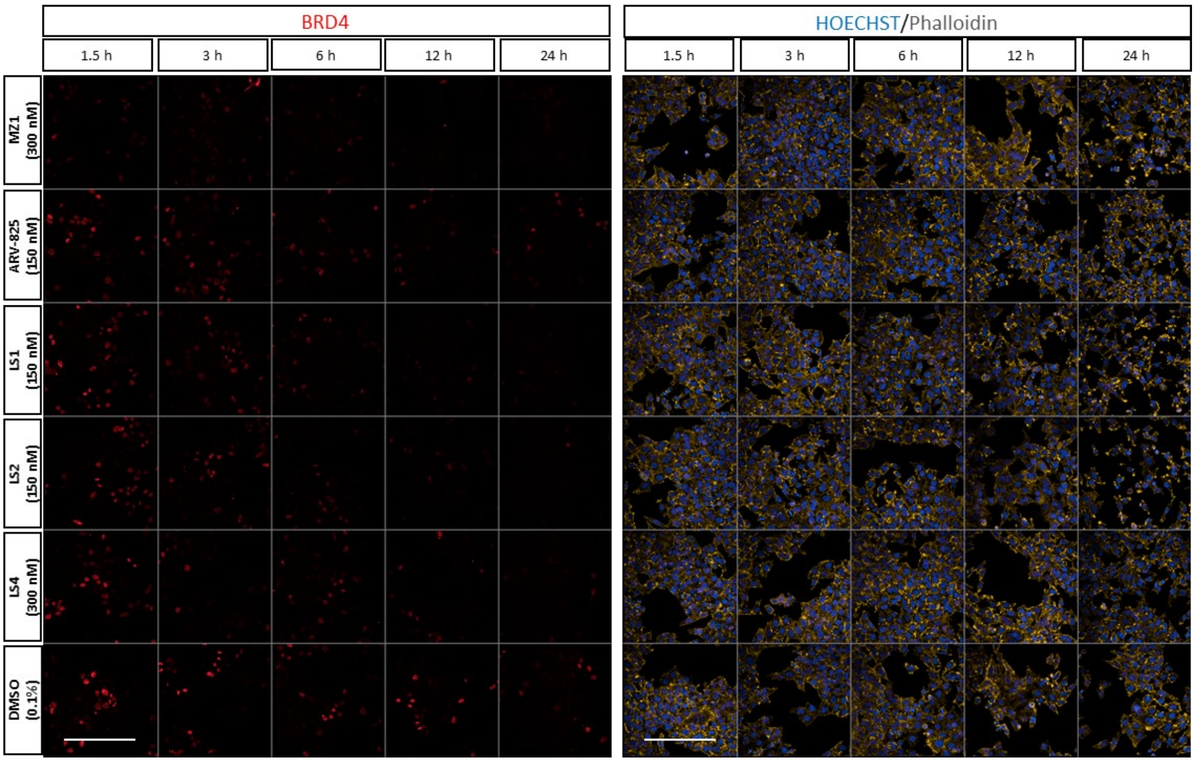

Fig. S5| Representative images showing nuclear BRD4 signals (left) analysed through the pipeline after treatment with BRD4 PROTACs at different timepoints. Images showing HeLa cells (right) at the same field of view as BRD4 images. Red: BRD4 (Alexa Fluor 647 secondary antibody signal), blue: cell nuclei (HOECHST), yellow: actin (Alexa Fluor 568 phalloidin)). Treatments: **MZ1** 300 nM, **ARV825** 150 nM, **LS1** 150 nM, **LS2** 150 nM, **LS4** 300 nM. DMSO at 0.1%. Scale bar: 200 µm.

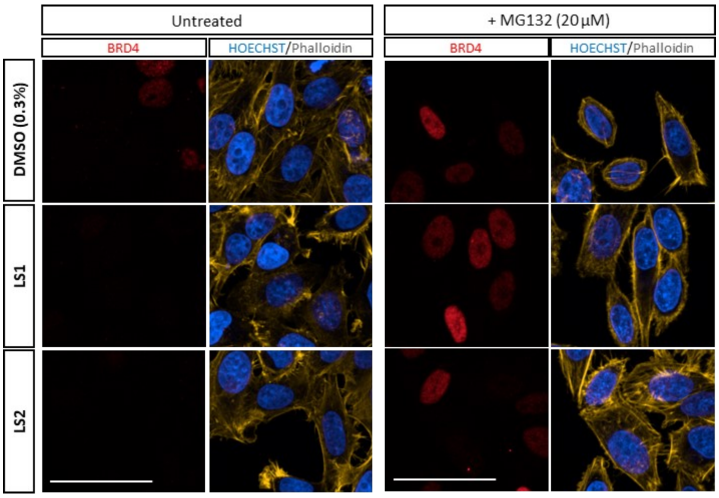

Fig. S6| Representative images to demonstrate BRD4 is degraded via UPS in cells. HeLa cells were treated for 12 h with proteasome inhibitor **MG132**, and/or **LS1** and **LS2** (Concentrations: **MG132** 20 µM, **LS1** 150 nM, **LS2** 150 nM). Red: BRD4 (Alexa Fluor 647 secondary antibody signal), blue: cell nuclei (HOECHST), yellow: actin (Alexa Fluor 568 phalloidin)). DMSO at 0.3%. Scale bar: 50 µm.

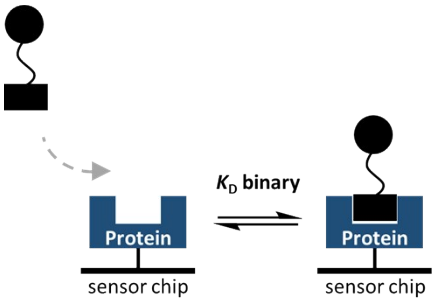

Fig. S7| Schematic representation of the SPR assay performed to measure binary complex formation. The proteins ($BRD4^{BD1}$, $BRD4^{BD2}$ or CRBN) were immobilised on a sensor chip, and PROTAC compounds were injected at various concentrations.

Table S1. Binding kinetics of PROTAC binary complexes with immobilised $BRD4^{BD1}$, $BRD4^{BD2}$ or CRBN measured by Surface Plasmon Resonance (SPR).

Compound	Protein	K_D (nM)	k_{on} ($M^{-1}s^{-1}$) × 10^5	k_{off} (s^{-1})
**(+)-JQ1**	$BRD4^{BD1}$	56	5.6	0.035
	$BRD4^{BD2}$	118	1.3	0.0076
	CRBN	NA	NA	NA
**ARV825**	$BRD4^{BD1}$	444	2.8	0.050
	$BRD4^{BD2}$	35	0.48	0.0015
	CRBN	686	11.1	0.76
**LS1**	$BRD4^{BD1}$	416	0.45	0.014
	$BRD4^{BD2}$	464	0.03	0.0015
	CRBN	1320	0.051	0.0064
**LS2**	$BRD4^{BD1}$	1235	0.156	0.020
	$BRD4^{BD2}$	187	0.052	0.00092
	CRBN	1810	0.99	0.10
**LS3**	$BRD4^{BD1}$	704	0.32	0.020
	$BRD4^{BD2}$	628	0.065	0.0036
	CRBN	19250	0.0066	0.0121
**LS4**	$BRD4^{BD1}$	741	0.27	0.019
	$BRD4^{BD2}$	1075	0.051	0.0054
	CRBN	1060	0.013	0.0014

SPR values were derived by selection of Kinetic fit model (1:1 binding). From dissociation constant ($K_D = k_{off}/k_{on}$). (n = 2 independent experiments, n = 1 for **ARV825** and **LS4** for CRBN binding experiment).

Table S2. Tabulated list of proteins used in SPR binary and ternary assays respectively.

Protein name	Protein MW	Sourced proteins details	Protein stock concentration
BRD4^{BD1}	15.5 kDa	In-house, UCB	20.5 µg/µl
BRD4^{BD2}	15 kDa	In-house, UCB	8.68 µg/µl
CRBN (Full-length GST Tag)	78 kDa	Sino Biological, Cat# C55-30G, Lot# J5325-10	0.05 µg/µl

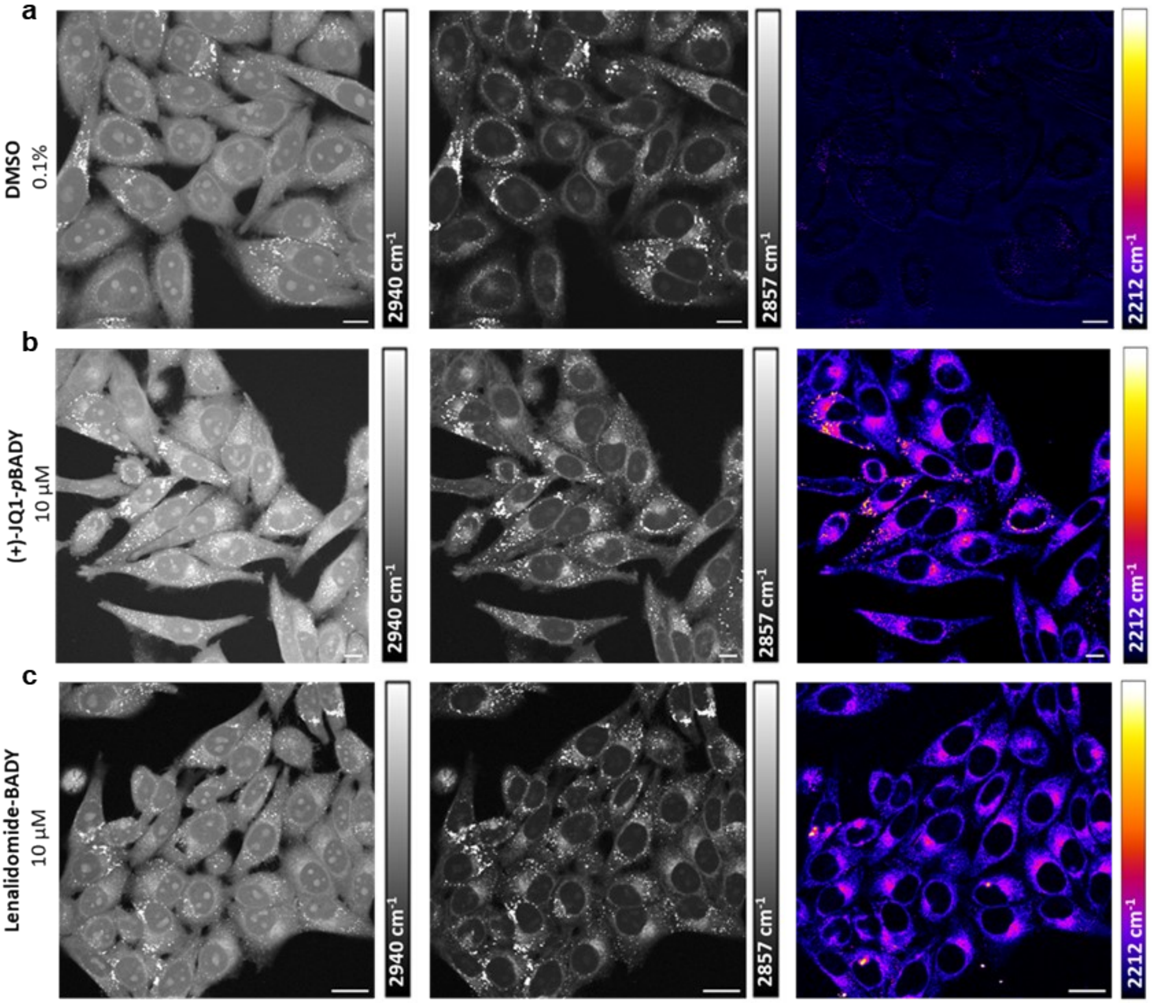

Fig. S8| SRS imaging of live HeLa cells incubated with controls. (**a**) 0.1% DMSO for 4 h. (**b**) 10 µM of **(+)-JQ1-*p*BADY** for 3 h. (**c**) 10 µM of **Lenalidomide-BADY** for 4 h. Images acquired at (L-R): 2940 cm^{-1} (CH_3, proteins), 2844 cm^{-1} (CH_2, lipids), 2212 cm^{-1} (C≡C, diyne). Alkyne images are background subtracted. (**a** and **b**, scale bar: 10 µm, **c**: scale bar: 20 µm)

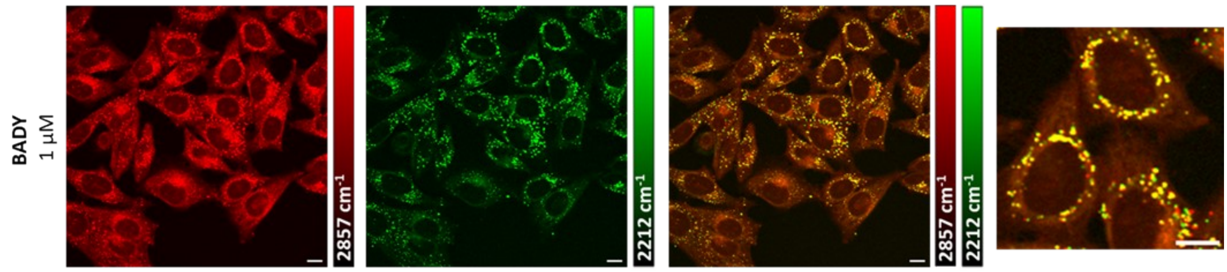

Fig. S9| BADY is accumulated within lipid droplets in the cytoplasm of the cells. HeLa cells were treated with 1 µM of **BADY** for 3 h. Images acquired at (L-R): 2844 cm^{-1} (CH_2, lipids), 2212 cm^{-1} (C≡C, diyne), merge of images acquired and zoomed merged image. Colocalisation analysis between 2844 cm^{-1} (CH_2, lipids) and 2212 cm^{-1} (C≡C, diyne) images, revealed a Pearson's coefficient of r = 0.68, scale bar: 10 µm.

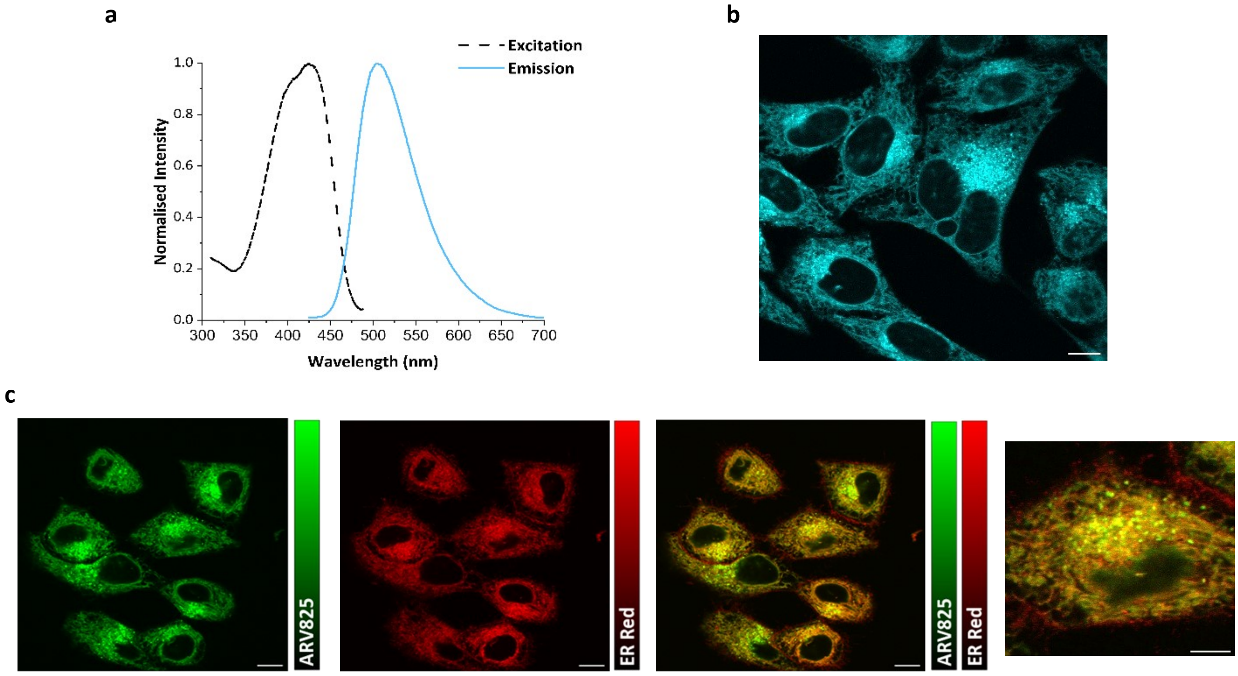

Fig. S10| Label-free Intracellular imaging of ARV825 in live HeLa cells. (**a**) Absorption and fluorescence emission spectra of a 100 µM **ARV825** solution in FluoroBrite DMEM. (**b**) Representative image of **ARV825** after HeLa cells were treated for 2 h with 1 µM (excitation laser: 405 nm, Scale bar: 10 µm). (**c**) HeLa cells were treated with 1 µM of **ARV825** and 1 µM of ER-Tracker Red for 3 h. Images acquired at (L-R): **ARV825**, ER-Tracker Red (excitation laser: 561 nm), merge of images acquired and zoomed merged image. Colocalisation analysis between **ARV825** and ER-Tracker Red images, revealed a Pearson's coefficient of r = 0.81 (Scale bar: 10 µm, zoomed image scale bar: 5 µm).

Fluorescence Imaging

Confocal images were captured with an Olympus FV3000 Confocal Laser Scanning Microscope using Fluoview FV31S-SW (version 2.4.1.198) Software. The microscope consists of an IX83 inverted frame and galvanometer scanhead coupled to a laser bed containing 405, 432, 488, 515, 561 and 640 nm laser lines. Fluorescence images were captured using an Olympus UPlanSApo 60× 0.95NA objective and detected by high sensitivity GaAsP photomultiplier tubes. Fluorescence signals were resolved onto the detectors using a series of dichroic mirrors and filters combined with spectral gratings selected as appropriate by the software. Images were scanned at 1024×1024 size, between 2-4 zoom and with 2× Kalman averaging. Images were further analysed in FIJI/ImageJ (version 1.54f).

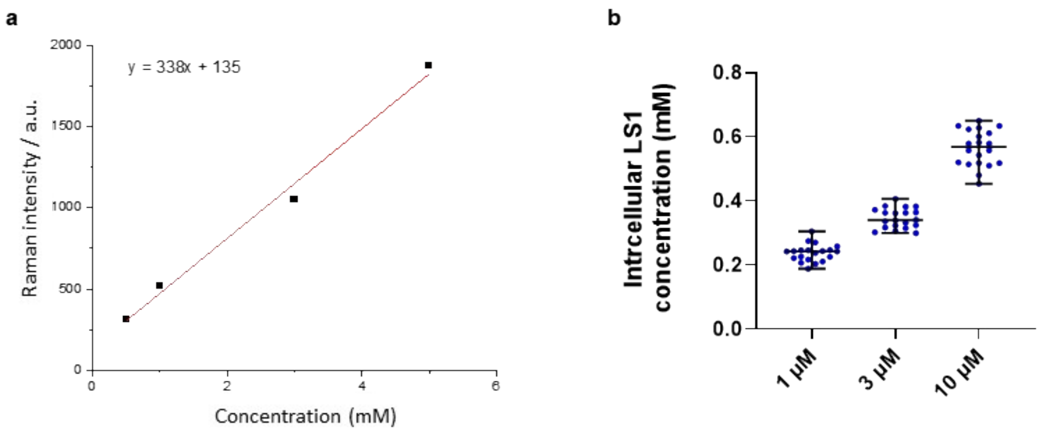

Fig. S11| Correlation between SRS intensity of LS1 measured at 2212 cm^{-1} and LS1 concentration. (**a**) **LS1** DMSO stock solutions at different concentrations (0.5, 1, 3, 5 mM) were imaged, and maximum intensities were plotted against concentration to derive the calibration line equation. (**b**) Mean intracellular **LS1** concentration in HeLa cells. Cells were treated with 10 µM, 3 µM, or 1 µM of **LS1** for 5-6 h. The Raman intensity of 20 individual cells was quantified and converted to mM concentrations using the calibration equation.

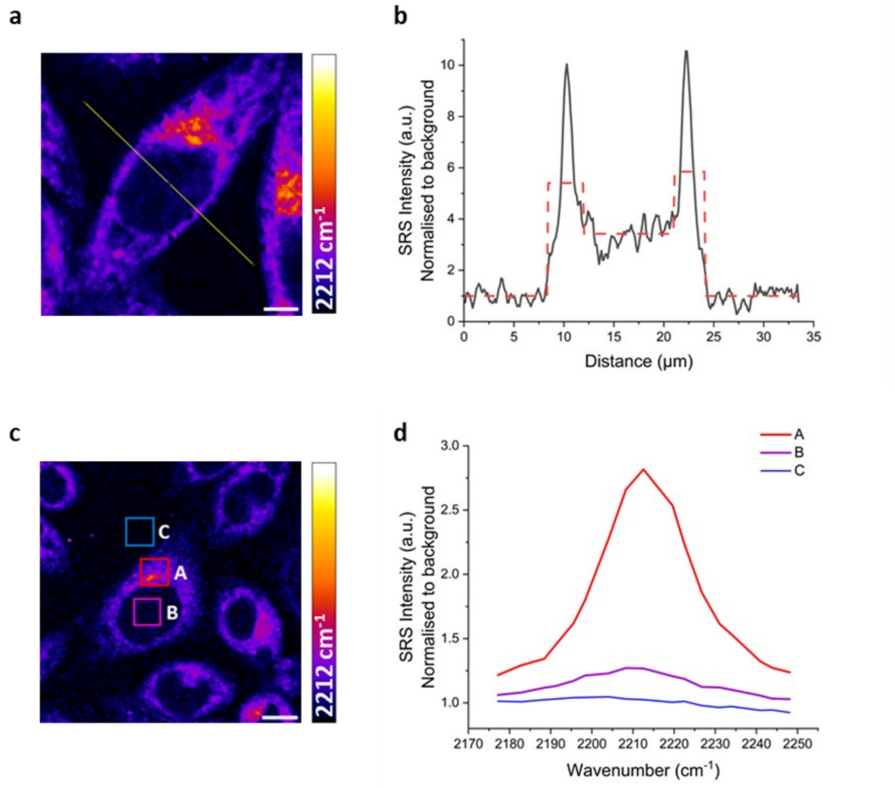

Fig. S12| SRS images and spectral information of live HeLa cells incubated with 10 μM LS1 for 1 h. (a) Representative image of **LS1** acquired at 2212 cm^{-1} (C≡C, diyne, scale bar: 5 μm), **(b)** SRS intensity profile of **LS1** along the yellow line from **(a)** (left to right). The red dashed line indicates the average intensity within each region (background, cytoplasm, and nucleus). **(c)** Representative image of **LS1**, acquired at 2212 cm^{-1} (C≡C, diyne) during hyperspectral imaging (2177-2248 cm^{-1}, scale bar: 10 μm). **(d)** SRS spectral information showing the **LS1** signal intensity within the boxed regions from **(c)**. (A) red: cytoplasm/endoplasmic reticulum, (B) purple: nucleus, (C) blue: background.

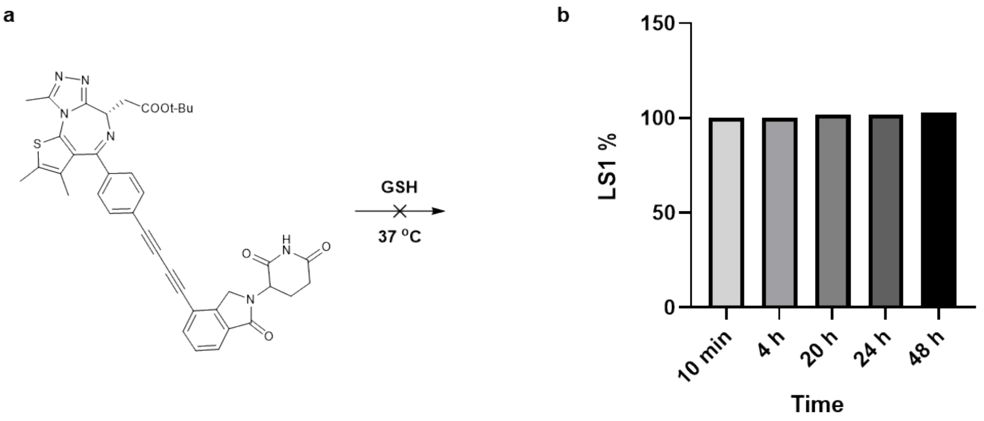

Fig. S13| Stability assessment of LS1 in the presence of GSH. (a) Schematic representation of the assay. **LS1** (100 μM) was mixed with **GSH** (1 mM) in PBS/ACN (1:1) and stirred at 37 °C. **(b)** The reaction mixture was tested by LC-MS at different time points. The percentage of **LS1** was determined from the area under the curve of LC-MS chromatograms at 280.4 nm and normalised to the **LS1** (100 μM) solution in PBS/ACN (1:1).

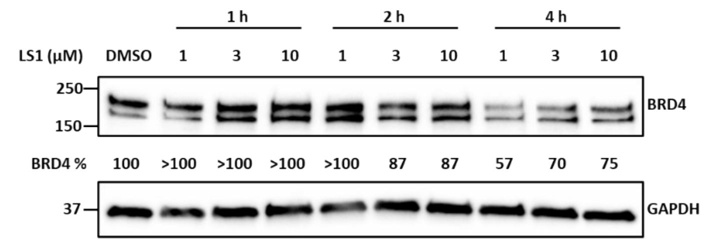

Fig. S14| Western blot analysis of BRD4 in HeLa cells treated with LS1 at imaging concentrations. HeLa cells were treated with **LS1** at different time points.

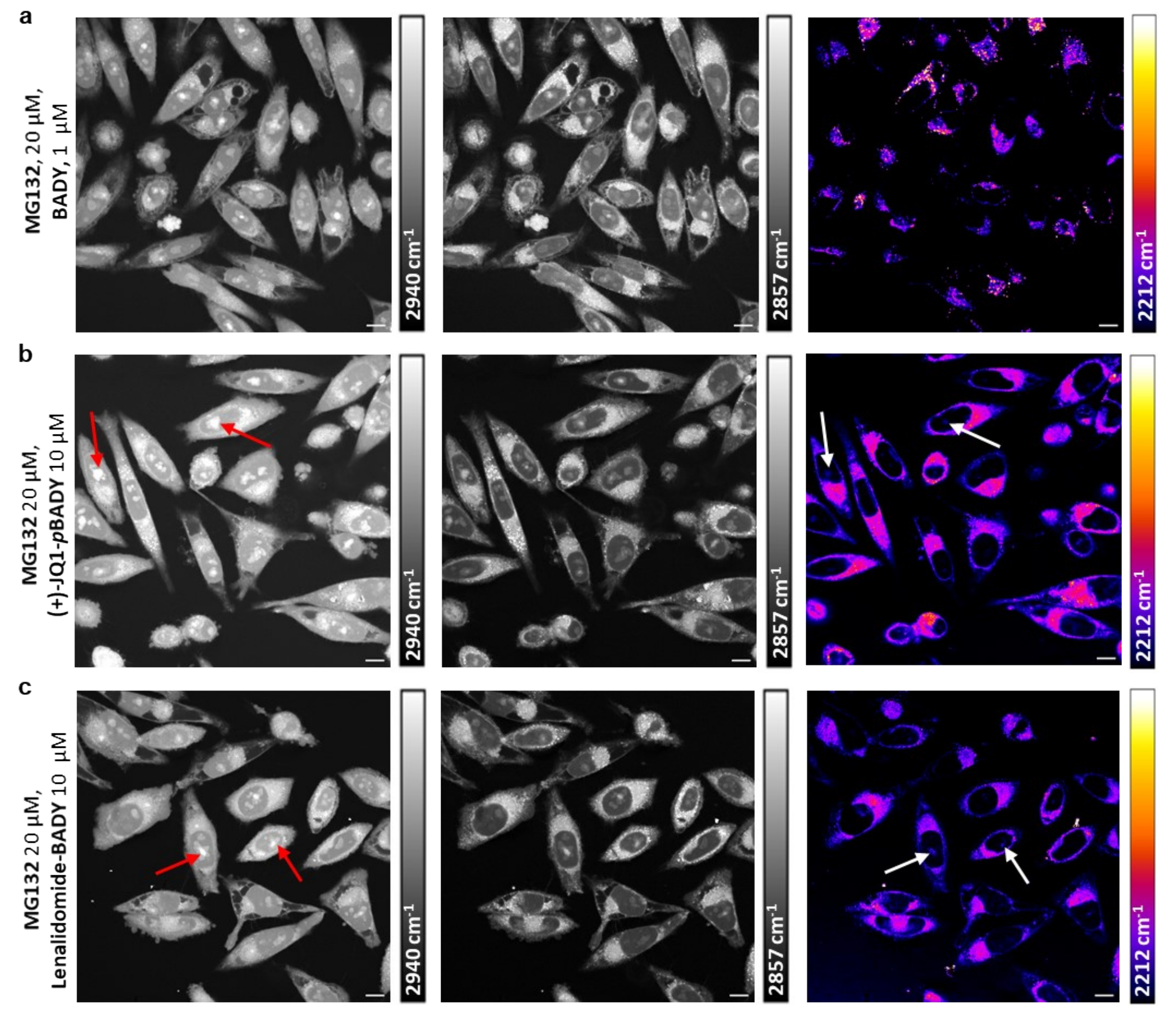

Fig. S15 | SRS imaging of live HeLa cells after proteasomal inhibition and incubated with controls. (**a**) 20 µM of **MG132** for 16 h and 1 µM of **BADY** for 2 h. (**b**) 20 µM of **MG132** for 16 h and 10 µM of **(+)-JQ1-*p*BADY** for 3 h. (**c**) 20 µM of **MG132** for 16 h and 10 µM of **Lenalidomide-BADY** for 2 h. Images acquired at (L-R): 2940 cm^{-1} (CH_3, proteins), 2844 cm^{-1} (CH_2, lipids), 2212 cm^{-1} (C≡C, diyne). Red/white arrows indicate the nucleolar aggregates where control compounds are co-localised. Scale bar: 10 µm.

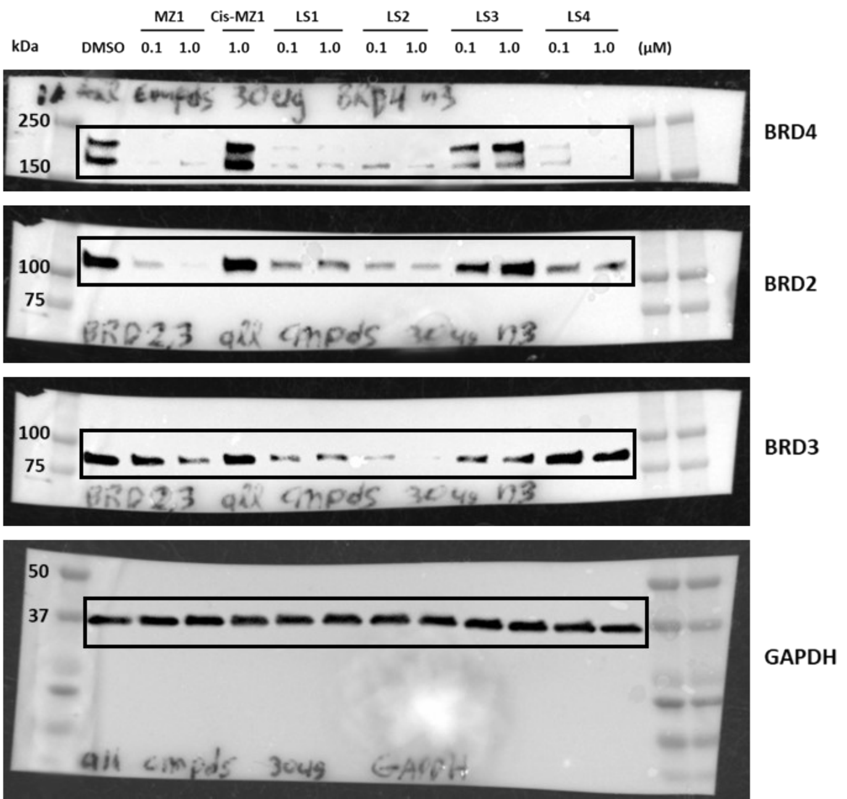

Fig. S16| Uncropped Western blot for Fig. 1b.

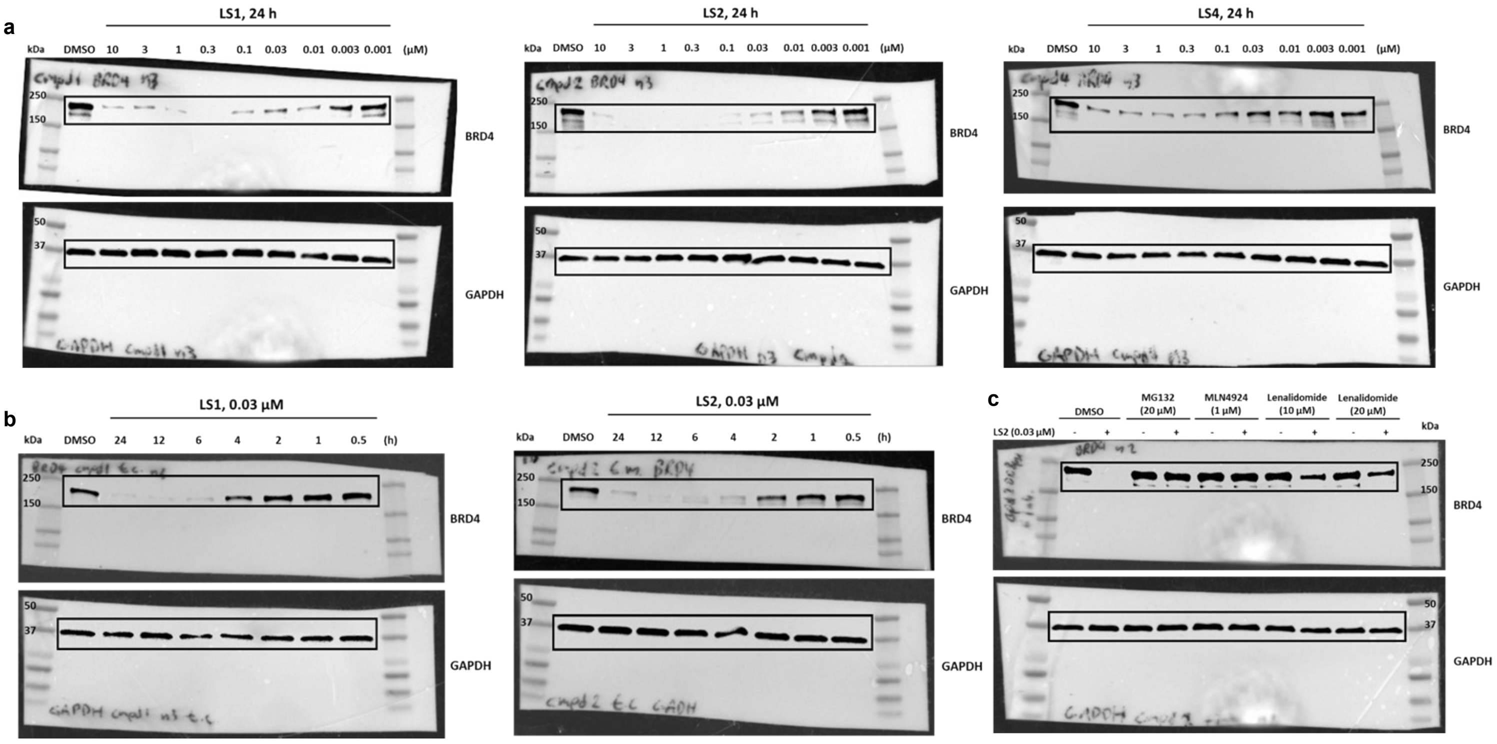

Fig. S17| Uncropped Western blots from Fig. 2a (**a**), Fig. 2b (**b**), and from Fig. 2f (**c**).

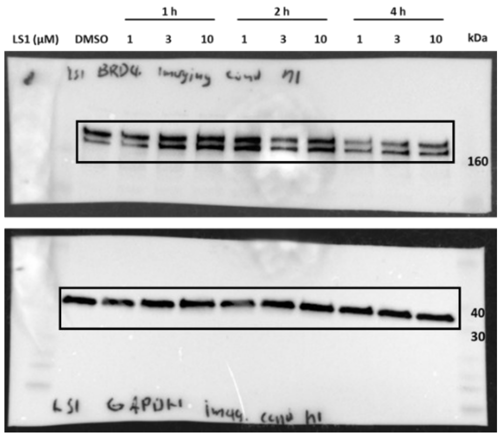

Fig. S18| Uncropped western blot for Fig. S14.

MD Simulation Details

Each system was solvated in a water box extending at least 8 Å from the protein in each direction, with sodium ions added to neutralise the system charge, using AmberTools.[1] The AMBER ff14SB force field was used to model the protein, the TIP3P force field for the water, Joung-Cheatham parameters for neutralising Na^+ ions, and the tetrahedral nonbonded dummy model was used for the CRBN-bound Zn^{2+} ion.[2–6] GAFF parameters with AM1-BCC charges were used for the ligand.[7–9] A cutoff of 12 Å was used for non-bonded interactions, with Particle Mesh Ewald used to calculate the contribution of long-range electrostatic interactions.[10] A Langevin integrator (timestep of 2 fs, and friction coefficient of 1 ps^{-1}) was used to simulate these systems at a constant temperature of 298 K, with a Monte Carlo barostat (applied every 25 timesteps) used to maintain a pressure of 1 bar.[11] The SETTLE algorithm was used to constrain water molecules, and the SHAKE algorithm was used to constrain all other bonds involving hydrogen atoms.[12–14]

Chemical and Analytical Methods

Reactions indicated as performed under inert atmosphere were carried out with a Schlenk line with flame-dried glassware prepared using circulation between vacuum and nitrogen. When employing Schlenk line technique, anhydrous solvents were used from commercial septated bottles (DMF) or a solvent purification system (THF) and transferred using argon or nitrogen. All other reactions were performed open to ambient conditions. A temperature of 18 – 22 °C can be assumed for reactions conducted at room temperature, unless explicitly indicated. IUPAC names of the chemical structures were generated on Marvinsketch.

All commercial starting materials were used as received from Fluorochem, Cayman Chemical, Acros, Fisher Scientific or Sigma Aldrich / Merck, unless explicitly stated. Flash chromatography columns were performed using CombiFlash® NextGen 100 system with RediSep® pre-packed silica column or manually using ACROS Organics™ or Fisher-Thermo Scientific silica gel (0.035-0.070 mm, 60 Å). Reactions were monitored by normal phase thin-layer chromatography (TLC) using Merck 60 F254 silica gel foil-backed plates with 0.2 mm coating thickness. Compounds were visualised by exposure of the plate to UV lights (254 nm).

Infrared (IR) spectra were obtained using a Shimadzu IRAffinity-1 solid state FT-IR spectrometer with neat samples. Characteristic bands for absorbances ≥ 1600 cm^{-1} are reported. Nuclear magnetic resonance (NMR) spectra were recorded at ambient temperature using a Bruker AVA500 or AVA600 spectrometer operating at 500 or 601 MHz (^{1}H spectra) and 126 or 151 MHz (^{13}C spectra), respectively. Residual solvent peaks ($CDCl_3$: ^{1}H δ 7.26 ppm, ^{13}C δ 77.16 ppm; DMSO-d_6: ^{1}H δ 2.50 ppm, ^{13}C δ 39.52 ppm.) were used as an internal reference. The abbreviations s = singlet, d = doublet, t = triplet, q = quartet, m = multiplet and br = broad (or combinations thereof) were used to describe the peak multiplicity in ^{1}H NMR spectra. Coupling constants (*J*) are adjusted to the nearest 0.1 Hz. ^{13}C NMR signals are assigned as quaternary (C), tertiary (CH), secondary (CH_2) or primary (CH_3) with appropriate coefficients (i.e., 2CH) for symmetrical compounds. LC-MS/MS experiments were performed using Agilent1200 instrument (pump Agilent G1312B SL binary, AutoSampler Agilent G1367A WP, Agilent G1316B Column oven, Agilent G1315C Starlight DAD) with a Phemoenex Kinetex C18 50×2.1 column and gradient methods of 10.02 min using 0.1% formic acid/water and 0.1% formic acid/MeCN as eluents.

Synthetic Procedures

Scheme S1. Synthesis of **(+)-JQ1** and its TIPS-protected alkyne derivative **(7)**.

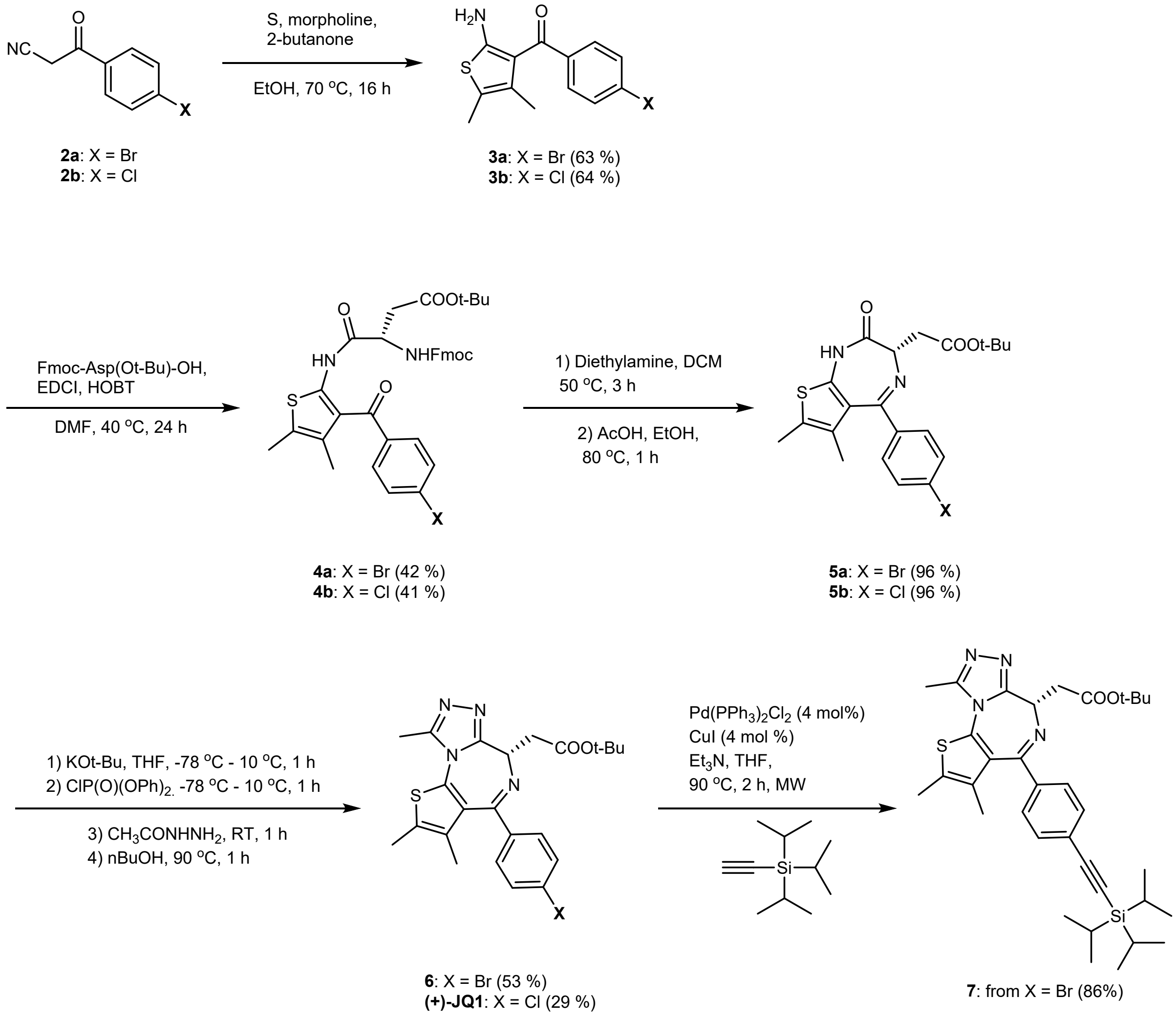

3-(4-Bromobenzoyl)-4,5-dimethylthiophene-2-amine (3a)

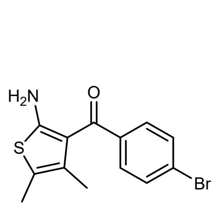

To a solution of 4-bromobenzoyl acetonitrile **2a** (4.00 g, 17.5 mmol), 2-butanone (4.79 mL, 52.5 mmol) and morpholine (2.32 mL, 26.8 mmol) in EtOH (80 mL) was added sulphur (1.14 g, 35.0 mmol). The mixture was heated to 70 °C. After 16 h, the mixture was cooled to room temperature, the solvent was evaporated, and the residue was poured into brine (80 mL). The aqueous layer was extracted with EtOAc (3 × 50 mL). The combined organic residues were washed with brine (50 mL), dried over $MgSO_4$, and concentrated *in vacuo*. The residue was purified using flash column chromatography (0 to 10 % EtOAc in Petroleum Ether 40-60) to give amine **3a** as a yellow solid (3.49 g, 63 %). **R$_f$** (10 % EtOAc in Petroleum Ether 40-60) = 0.42. **^{1}H NMR** (500 MHz, $CDCl_3$) δ 7.55 (d, *J* = 8.5 Hz, 2H, Ar*H*), 7.40 (d, *J* = 8.5 Hz, 2H, Ar*H*), 6.44 (br s, 2H, N*H*$_2$), 2.13 (s, 3H, C*H*$_3$), 1.56 (s, 3H, C*H*$_3$). **^{13}C NMR** (126 MHz, $CDCl_3$) δ 191.63 (C=O), 163.09 (ArC), 140.59 (ArC), 131.35 (2ArCH), 129.63 (2ArCH), 128.46 (ArC), 125.10 (ArC), 117.14 (ArC), 115.36 (ArC), 15.53

(CH_3), 12.55 (CH_3). **HRMS** (ESI) $[^{79}BrM+Na]^+$ found 331.9715, $C_{12}H_{14}{}^{79}BrNOSNa$ requires 331.9721, $[^{81}BrM+Na]^+$ found 333.9695, $C_{12}H_{14}{}^{81}BrNOSNa$ requires 333.9701.

3-(4-Chlorobenzoyl)-4,5-dimethylthiophene-2-amine (3b)

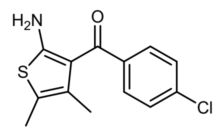

To a solution of 4-chlorobenzoyl acetonitrile **2b** (2.50 g, 13.9 mmol), 2-butanone (3.70 mL, 41.8 mmol) and morpholine (1.80 mL, 20.9 mmol) in ethanol (60 mL) was added Sulphur (0.893 g, 27.8 mmol). The mixture was heated to 70 °C. After 16 h, the mixture was cooled to room temperature, the solvent was evaporated, and the residue was poured into brine (80 mL). The aqueous layer was extracted with EtOAc (3 × 50 mL). The combined organic residues were washed with brine (50 mL), dried over $MgSO_4$, and concentrated *in vacuo*. The residue was purified using flash column chromatography (0 to 10 % EtOAc in Petroleum Ether 40-60) to give amine **3b** as an orange solid (2.37 g, 64 %). $\mathbf{R_f}$ (10 % EtOAc in Petroleum Ether 40-60) = 0.27. $\mathbf{^1H}$ **NMR** (500 MHz, $CDCl_3$) δ 7.48 (d, *J* = 8.6 Hz, 2H, Ar*H*), 7.37 (d, *J* = 8.6 Hz, 2H, Ar*H*), 6.41 (br s, 1H, NH_2), 2.13 (s, 3H, CH_3), 1.56 (s, 3H, CH_3). $\mathbf{^{13}C}$ **NMR** (126 MHz, $CDCl_3$) δ 191.58 (C=O), 162.98 (ArC), 140.13 (ArC), 136.72 (ArC), 129.47 (2ArCH), 128.40 (2ArCH), 117.21 (ArC), 115.37 (ArC), 102.98 (ArC), 15.50 (CH_3), 12.55 (CH_3).

The spectroscopic data are in good agreement with those reported in literature.[15]

tert-Butyl (*S*)-3-((((9*H*-fluoren-9-yl)methoxy)carbonyl)amino)-4-((3-(4- bromo benzoyl)-4,5-dimethylthiophen-2-yl)amino)-4-oxobutanoate (4a)

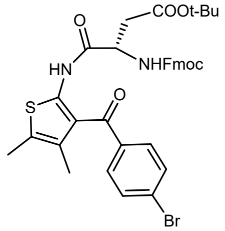

To a solution of Fmoc-Asp-(O*t*-Bu)-OH (6.95 g, 16.9 mmol) in DMF (23 mL) were added EDCI (2.63 g, 16.9 mmol) and HOBt (0.91 g, 6.75 mmol). The mixture was stirred at room temperature for 10 min, and then amine **3a** (3.49 g, 11.2 mmol) was added. The resulting mixture was stirred at 40 °C. After 24 h, the mixture was cooled to room temperature, and the residue was diluted with toluene (50 mL) and washed with brine (100 mL). The aqueous phase was extracted with toluene (2 × 50 mL). The combined organic residues were washed with brine (50 mL), dried over $MgSO_4$, and concentrated *in vacuo*. The residue was purified using flash column chromatography (0 to 15 % EtOAc in Petroleum Ether 40-60) to give amide **4a** as an orange solid (3.44 g, 43 %) with recovery of amine **3a** (1.22 g, 3.92 mmol). $\mathbf{R_f}$ (15 % EtOAc in Petroleum Ether 40-60) = 0.35. $\mathbf{^1H}$ **NMR** (601 MHz, $CDCl_3$) δ 11.75 (s, 1H, N*H*), 7.75 (d, *J* = 7.6 Hz, 2H, Ar*H*), 7.67 – 7.58 (m, 2H, Ar*H*), 7.50 –7.47 (m, 2H, Ar*H*), 7.40 – 7.34 (m, 4H, Ar*H*), 7.26 – 7.19 (m, 2H, Ar*H*), 6.13 (d, *J* = 9.2 Hz, 1H, N*H*), 4.82 – 4.76 (m, 1H, C*H*), 4.62 (t, *J* = 8.5 Hz, 1H, C*H*), 4.34 – 4.23 (m, 2H, CH_2), 3.13 (dd, J = 17.1, 4.9 Hz, 1H, CH_AH_B), 2.75 (dd, *J* = 17.1, 4.9 Hz, 1H, CH_AH_B), 2.28 (s, 3H, CH_3), 1.67 (s, 3H, CH_3), 1.43 (s, 9H, CH_3). $\mathbf{^{13}C}$ **NMR** (126 MHz, $CDCl_3$) δ 193.36 (C=O), 170.75 (C=O), 168.57 (C=O), 156.41 (C=O), 145.83 (ArC), 144.31 (ArC), 143.68 (ArC), 141.44 (ArC), 141.35 (ArC), 139.00 (ArC), 131.65 (2ArCH), 130.32 (2ArCH), 127.84 (ArCH), 127.79 (ArCH), 127.23 (ArCH), 127.16 (ArCH), 126.83 (2ArCH), 125.51 (2ArC), 125.33 (ArC), 123.17 (ArC), 120.07 (ArCH), 120.04 (ArCH), 82.34 (C), 68.04 (CH_2), 47.29 (CH), 40.99 (CH_2), 28.19 $(3CH_3)$, 23.97 (CH), 15.16 (CH_3), 12.69 (CH_3). **HRMS** (ESI) $[^{79}BrM+Na]^+$ found 725.1291, $C_{36}H_{35}{}^{79}BrN_2O_6SNa$ requires 725.1297, $[^{81}BrM+Na]^+$ found 727.1277, $C_{36}H_{35}{}^{81}BrN_2O_6SNa$ requires 727.1276.

tert-Butyl (S)-3-((((9H-fluoren-9-yl)methoxy)carbonyl)amino)-4-((3-(4-chlorobenzoyl)-4,5-dimethylthiophen-2-yl)amino)-4-oxobutanoate (4b)

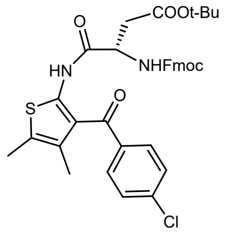

To a solution of Fmoc-Asp-(O*t*-Bu)-OH (5.47 g, 13.3 mmol) in DMF (25 mL) were added EDCI (2.06 g, 13.3 mmol) and HOBt (0.72 g, 5.732 mmol). The mixture was stirred at room temperature for 10 min, and then amine **3b** (2.36 g, 8.87 mmol) was added. The resulting mixture was stirred at 40 °C. After 24 h, the mixture was cooled to room temperature, and the residue was diluted with toluene (50 mL) and washed with brine (100 mL). The aqueous phase was extracted with toluene (2 × 50 mL). The combined organic residues were washed with brine (50 mL), dried over $MgSO_4$, and concentrated *in vacuo*. The residue was purified using flash column chromatography (0 to 15 % EtOAc in Petroleum Ether 40-60) to give amide **4b** as an orange solid (2.42 g, 41 %) with recovery of amine **3b** (1.22 g, 3.92 mmol). $\mathbf{R_f}$ (15 % EtOAc in Petroleum Ether 40-60) = 0.41. **^{1}H NMR** (500 MHz, $CDCl_3$) δ 11.73 (s, 1H, N*H*), 7.75 (d, *J* = 7.6 Hz, 2H, Ar*H*), 7.66 – 7.60 (m, 2H, Ar*H*), 7.44 (d, *J* = 8.3 Hz, 2H, Ar*H*), 7.38 (t, *J* = 7.4 Hz, 2H, Ar*H*), 7.32 (d, *J* = 8.3, 2H, Ar*H*), 7.26-7.20 (m, 2H, Ar*H*), 6.10 (d, *J* = 9.1 Hz, 1H, N*H*), 4.78 (br s, 1H, C*H*), 4.62 (br s, 1H, C*H*), 4.34 – 4.24 (m, 2H, CH_2), 3.13 (dd, *J* = 17.0, 4.9 Hz, 1H, CH_AH_B), 2.73 (dd, *J* = 17.0, 4.9 Hz, 1H, CH_AH_B), 2.26 (s, 3H, CH_3), 1.68 (s, 3H, CH_2), 1.43 (s, 9H, 3CH_3). **^{13}C NMR** (126 MHz, $CDCl_3$) δ 193.23 (C=O), 170.72 (C=O), 168.55 (C=O), 156.40 (C=O), 145.70 (ArC), 144.30 (ArC), 143.67 (ArC), 141.43 (ArC), 138.53 (ArC), 130.20 (2ArCH), 128.68 (2ArCH), 127.78 (ArCH), 127.22 (2ArCH), 127.15 (2ArCH), 125.51 (ArC), 125.32 (ArC), 123.23 (ArC), 120.05 (ArCH), 120.03 (ArCH), 82.32 (C), 68.02 (CH_2), 51.86 (CH), 47.28 (CH), 36.91 (CH_2), 28.18 (3CH_3), 15.11 (CH_3), 12.67 (CH_3).

The spectroscopic data are in good agreement with those reported in literature.[15]

tert-Butyl (S)-2-(5-(4-bromophenyl)-6,7-dimethyl-2-oxo-2,3-dihydro-1H-thieno [2,3-e][1,4]diazepin-3-yl)acetate (5a)

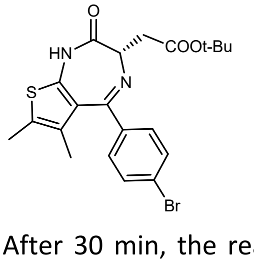

To a solution of amide **4a** (3.44 g, 4.89 mmol) in DCM (25 mL) was added diethylamine (2.96 mL, 29.4 mmol). The reaction solution was refluxed at 50 °C. After 3 h, the mixture was cooled to room temperature and the residue was concentrated *in vacuo* to give an orange oil which was used for the next step without further purification. The crude amide was dissolved in EtOH (25 mL) and acetic acid (2.80 mL, 48.9 mmol) was added. The mixture was refluxed at 80 °C. After 30 min, the reaction was cooled to room temperature and the solvent was removed under reduced pressure. The residue was purified using flash column chromatography (0 to 15 % EtOAc in Petroleum Ether 40-60) to give imine **5a** as a yellow solid (2.00 g, 88 %). $\mathbf{R_f}$ (20 % EtOAc in Petroleum Ether 40-60) = 0.45. **^{1}H NMR** (601 MHz, $CDCl_3$) δ 8.92 (s, 1H, N*H*), 7.51 (d, *J* = 8.9 Hz, 2H, Ar*H*), 7.38 (d, *J* = 8.9 Hz, 2H, Ar*H*), 4.23 (t, *J* = 6.6 Hz, 1H, C*H*), 3.36 (dd, *J* = 24.2, 7.4 Hz, 1H, CH_AH_B), 3.12 (dd, *J* = 24.2, 7.4 Hz, 1H, CH_AH_B), 2.30 (s, 3H, CH_3), 1.61 (s, 3H, CH_3), 1.50 (s, 9H, CH_3). **^{13}C NMR** (151 MHz, $CDCl_3$) δ 171.26 (C=O), 169.30 (C=O), 165.23 (C), 141.14 (ArC), 137.47 (ArC), 131.70 (2ArCH), 130.53 (2ArCH), 129.81 (ArC), 127.78 (ArC), 126.73 (ArC), 124.89 (ArC), 80.81 (C), 61.42 (CH), 37.63 (CH_2), 28.31 (3CH_3), 14.56 (CH_3), 13.00 (CH_3). **HRMS (ESI)** $[^{79}BrM+Na]^+$ found 485.0505, $C_{21}H_{23}{}^{79}BrN_2O_3SNa$ requires 485.0510, $[^{81}BrM+Na]^+$ found 487.0486, $C_{21}H_{23}{}^{81}BrN_2O_3SNa$ requires 487.0490.

***tert*-Butyl (*S*)-2-(5-(4-chlorophenyl)-6,7-dimethyl-2-oxo-2,3-dihydro-1*H*-thieno [2,3-*e*][1,4]diazepin-3-yl)acetate (5b)**

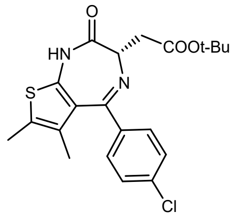

To a solution of amide **4b** (1.81 g, 2.75 mmol) in DCM (15 mL) was added diethylamine (1.66 mL, 16.5 mmol). The reaction solution was refluxed at 50 °C. After 3 h, the mixture was cooled to room temperature and the residue was concentrated *in vacuo* to give an orange oil which was used for the next step without further purification. The crude amine was dissolved in EtOH (25 mL) and acetic acid (1.43 mL, 24.9 mmol) was added. The mixture was refluxed at 80 °C. After 30 min, the reaction was cooled to room temperature and the solvent was removed under reduced pressure. The residue was purified using flash column chromatography (0 to 15 % EtOAc in Petroleum Ether 40-60) to give imine **5b** as an orange solid (1.11 g, 96 %). **R_f** (20 % EtOAc in Petroleum Ether 40-60) = 0.41. **^{1}H NMR** (500 MHz, $CDCl_3$) δ 8.99 (s, 1H, N*H*), 7.41 (d, *J* = 8.7 Hz, 2H, Ar*H*), 7.33 (d, *J* = 8.7 Hz, 2H, Ar*H*), 4.21 (t, *J* = 7.0 Hz, 1H, C*H*), 3.33 (dd, *J* = 16.9, 7.0 Hz, 1H, CH_AH_B), 3.10 (dd, *J* = 16.9, 7.0 Hz, 1H, CH_AH_B), 2.28 (s, 3H, CH_3), 1.59 (s, 3H, CH_3), 1.47 (ds, 9H, $3CH_3$). **^{13}C NMR** (126 MHz, $CDCl_3$) δ 171.13 (C=O), 169.25 (C=O), 164.99 (C=N), 141.02 (ArC), 136.91 (ArC), 136.34 (ArC), 130.16 (2ArCH), 129.68 (ArC), 128.61 (2ArCH), 127.71 (ArC), 126.58 (ArC), 80.66 (C), 61.26 (CH), 37.50 (CH_2) 28.16 ($3CH_3$), 14.41 (CH_3), 12.86 (CH_3).

The spectroscopic data are in good agreement with those reported in literature.[15]

***tert*-Butyl (*S*)-2-(4-(4-bromophenyl)-2,3,9-trimethyl-6*H*-thieno[3,2-*f*][1,2,4]triazolo[4,3-*a*][1,4]diazepin-6-yl)acetate (6)**

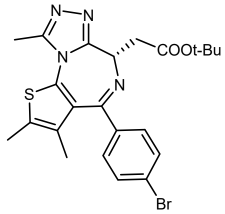

To a solution of imine **5a** (2.00 g, 4.31 mmol) in THF (20 mL) was added potassium *tert*-butoxide (0.969 g, 8.63 mmol) dropwise at -78 °C. The reaction mixture was warmed to -10 °C immediately. After 30 min, the mixture was cooled to -78 °C and diphenyl chlorophosphite (2.32 g, 8.63 mmol) was added. After 45 min, acetylhydrazide (0.959 g, 13.0 mmol) was added and the mixture was warmed to room temperature. After 1 h, 1-Butanol (25 mL) was added and the mixture was warmed to 90 °C and refluxed for 1 h. Then, the mixture was allowed to cool to room temperature and the solvent was removed *in vacuo*. The residue was redissolved in DCM (60 mL) and was washed with saturated $NaHCO_3$ (20 mL), brine (20 mL), dried over $MgSO_4$ and concentrated *in vacuo*. The residue was purified using flash column chromatography (0 to 100 % EtOAc in Petroleum Ether 40-60) to give triazole **6** as an orange solid (1.15 g, 53 %) with recovery of imine **5a** (0.700 g, 1.51 mmol). **R_f** (70 % EtOAc in Petroleum Ether 40-60) = 0.22. **$[a]^{22}_D$** = +48 (*c* 1.0, $CHCl_3$). **^{1}H NMR** (500 MHz, $CDCl_3$) δ 7.49 (d, *J* = 8.7 Hz, 2H, Ar*H*), 7.33 (d, *J* = 8.7 Hz, 2H, Ar*H*), 4.55 (dd, *J* = 7.7, 6.3 Hz, 1H, C*H*), 3.54 (dd, *J* = 17.0, 7.1 Hz, 2H, CH_AH_B), 3.52 (dd, *J* = 17.0, 7.1 Hz, 2H, CH_AH_B), 2.67 (s, 3H, CH_3), 2.41 (s, 3H, CH_3), 1.69 (s, 3H, CH_3), 1.50 (s, 9H, $3CH_3$). **^{13}C NMR** (126 MHz, $CDCl_3$) δ 171.00 (C=O), 163.83 (C=N), 155.65 (ArC), 149.94 (ArC), 137.32 (ArC), 132.50 (ArC), 131.81 (2ArCH), 130.96 (ArC), 130.77 (ArC), 130.46 (ArC), 130.17 (2ArCH), 125.26 (ArC), 81.07 (C), 54.11 (CH), 38.00 (CH_2), 28.32 ($3CH_3$), 14.57 (CH_3), 13.23 (CH_3), 12.03 (CH_3). **HRMS (ESI)** $[^{79}BrM+Na]^+$ found 523.0774, $C_{23}H_{25}{}^{79}BrN_4O_2SNa$ requires 523.0779, $[^{81}BrM+Na]^+$ found 525.0755, $C_{23}H_{25}{}^{81}BrN_4O_2SNa$ requires 525.0759.

***tert*-Butyl (*S*)-2-(4-(4-chlorophenyl)-2,3,9-trimethyl-6*H*-thieno[3,2-*f*][1,2,4]triazolo[4,3-*a*][1,4]diazepin-6-yl)acetate ((+)-JQ1)**

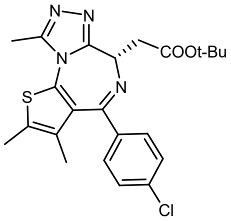

To a solution of imine **5b** (1.07 g, 2.56 mmol) in THF (12 mL) was added potassium *tert*-butoxide (0.48 mL, 3.84 mmol) dropwise at -78 °C. The reaction mixture was warmed to -10 °C immediately. After 30 min, the mixture was cooled to -78 °C and diphenyl chlorophosphite (1.03 g, 3.84 mmol) was added. After 45 min, acetylhydrazide (0.285 g, 3.84 mmol) was added and the mixture was warmed to room temperature. After 1 h, 1-Butanol (14 mL) was added and the mixture was warmed to 90 °C and heated under reflux for 1 h. Then, the mixture was allowed to cool to room temperature and the solvent was removed *in vacuo*. The residue was redissolved in DCM (60 mL) and washed with saturated $NaHCO_3$ (20 mL), brine (20 mL), dried over $MgSO_4$ and concentrated *in vacuo*. The residue was purified using flash column chromatography (0 to 100 % EtOAc in Petroleum Ether 40-60) to give **(+)-JQ1** as an orange solid (0.342 g, 29 %) with recovery of **5b** (0.504 g, 1.28 mmol). $\mathbf{R_f}$ (70 % EtOAc in Petroleum Ether 40-60) = 0.24. **Lit.** $\mathbf{[a]^{22}_{D}}$ = +55 (*c* 0.5, $CHCl_3$)[15], found $[a]^{22}_{D}$ = +40 (*c* 0.5, $CHCl_3$) **^{1}H NMR** (601 MHz, $CDCl_3$) δ 7.40 (d, *J* = 8.6 Hz, 2H, Ar*H*), 7.32 (d, *J* = 8.6 Hz, 2H, Ar*H*), 4.55 (dd, *J* = 14.2, 6.3 Hz, 1H, C*H*), 3.54 (dd, *J* = 17.0, 6.4 Hz, 1H, CH_AH_b), 3.53 (dd, *J* = 17.0, 6.4 Hz, 1H, CH_AH_b), 2.66 (s, 3H, CH_3), 2.40 (s, 3H, CH_3), 1.68 (s, 3H, CH_3), 1.49 (s, 9H, $3CH_3$). **^{13}C NMR** (151 MHz, $CDCl_3$) δ 170.95 (C=O), 163.71 (C=N), 155.64 (ArC), 149.91 (ArC), 136.84 (ArC), 136.81 (ArC), 132.43 (ArC), 130.93 (ArC), 130.76 (ArC), 130.50 (ArC), 129.92 (2ArCH), 128.81 (2ArCH), 81.03 (C), 54.05 (CH), 37.97 (CH_2), 28.29 ($3CH_3$), 14.52 (CH_3), 13.21 (CH_3), 11.99 (CH_3).

The spectroscopic data are in good agreement with those reported in literature.[15]

***tert*-Butyl 2-[(9*S*)-4,5,13-trimethyl-7{4[2(triisopropylsilyl)ethynyl]phenyl}-3-thia-1,8,11,12-tetraazatricyclo [8.3.0.0^{2,6}] trideca-2(6),4,7,10,12-pentaen-9-yl]acetate (7)**

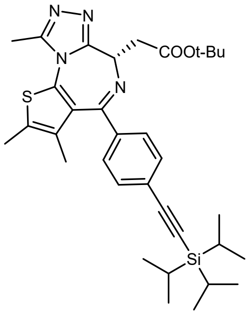

To an oven-dried microwave vial aryl bromide **6** (0.500 g, 1.00 mmol) was dissolved in anhydrous THF (5 mL). To the solution $Pd(PPh_3)_2Cl_2$ (0.028 g, 0.04 mmol), CuI (8.0 mg, 0.04 mmol), Et_3N (0.42 mL, 3.00 mmol) and TIPS-acetylene (0.546 g, 3.00 mmol) were added and stirred at 90 °C for 2 h under microwave conditions. The reaction mixture was diluted with ice-cold EtOAc (10 mL) and filtered through celite. The organic residue was concentrated *in vacuo*. The residue was purified using flash column chromatography (0 to 100 % EtOAc in Petroleum Ether 40-60) to give TIPS-protected alkyne **7** as a yellow oil (0.516 g, 86%). $\mathbf{R_f}$ (70 % EtOAc in Petroleum Ether 40-60) = 0.31. **^{1}H NMR** (601 MHz, $CDCl_3$) δ 7.44 (d, *J* = 8.6 Hz, 2H, Ar*H*), 7.39 (d, *J* = 8.6 Hz, 2H, Ar*H*), 4.56 (dd, *J* = 7.7, 6.3 Hz, 1H, C*H*), 3.57 – 3.53 (m, 2H, CH_2), 2.67 (s, 3H, CH_3), 2.40 (s, 3H, CH_3), 1.68 (s, 3H, CH_3), 1.50 (s, 9H, CH_3), 1.12 (m, 21H, $6CH_3$ and 3C*H*). **^{13}C NMR** (151 MHz, $CDCl_3$) δ 171.04 (C=O), 164.14 (C=N), 155.74 (ArC), 149.93 (ArC), 138.03 (ArC), 132.37 (ArC), 132.18 (2ArCH), 131.12 (ArC), 130.72 (ArC), 130.58 (ArC), 128.45 (2ArCH), 125.81 (ArC), 106.60 (C), 93.28 (C), 81.02 (C), 54.14 (CH), 38.05 (CH_2), 28.33 ($3CH_3$), 18.80 ($6CH_3$), 14.56 (CH_3), 13.22 (CH_3), 12.04 (CH_3), 11.45 (3CH). **HRMS** (ESI) $[M+Na]^+$ found 625.3003, $C_{34}H_{47}N_4O_2SSiNa$ requires 625.3004.

Scheme S2. Synthesis of **12**.

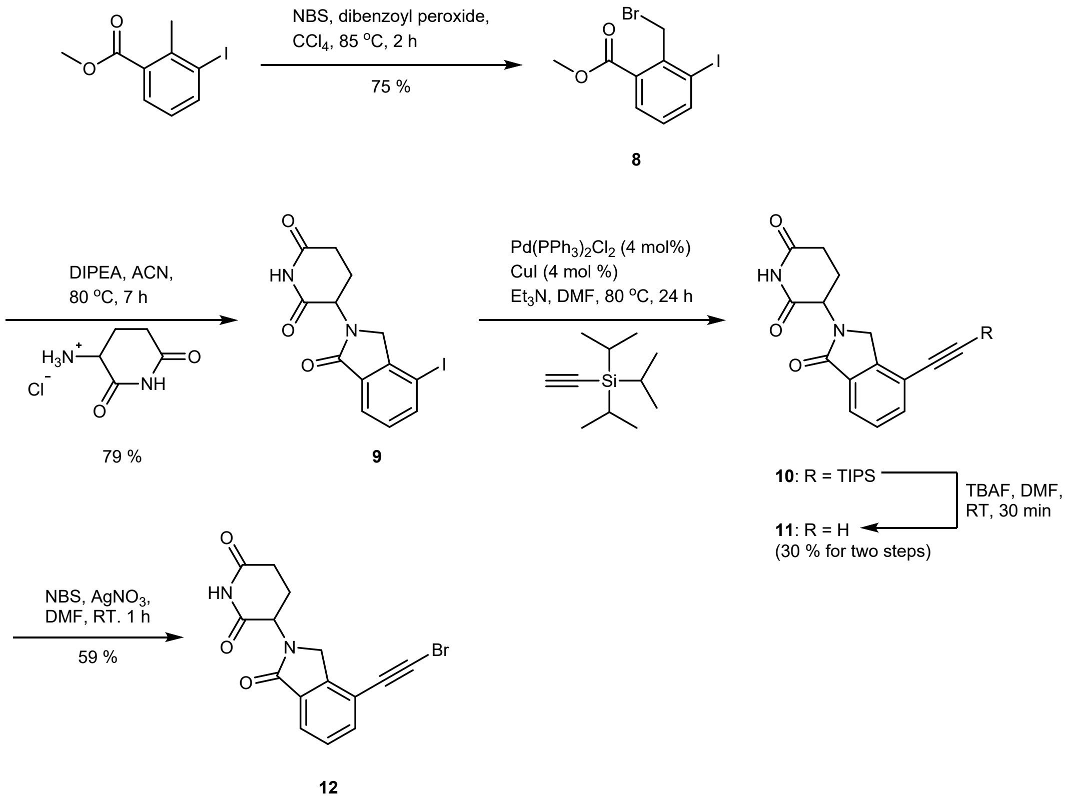

Methyl 2-(bromomethyl)-3-iodobenzoate (8)

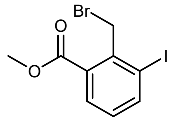

To a solution of methyl 3-iodo-2-methylbenzoate (0.1 g, 1.45 mmol) in CCl_4 (6 mL) were added NBS (0.387 g, 2.17 mmol) and dibenzoyl peroxide (0.067 g, 0.44 mmol). The reaction was warmed to 85 °C and heated under reflux for 4 h. After completion of the reaction, as indicated by TLC, the mixture was filtered. The filtrate was diluted with EtOAc (25 mL) and washed with $NaHCO_3$ (25 mL, sat. aq.) and brine (25 mL). The organic residue was dried over $MgSO_4$ and concentrated *in vacuo*. The product was purified using column chromatography (0-5 % EtOAc in Petroleum Ether 40-60) to obtain the bromide **8** as a yellow oil (0.352 g, 75%). $\mathbf{R_f}$ (3 % EtOAc in Petroleum Ether 40-60) = 0.56. **^{1}H NMR** (601 MHz, $CDCl_3$) δ 8.04 (dd, *J* = 7.6, 1.4 Hz, 1H, Ar*H*), 7.89 (dd, *J* = 7.6, 1.4 Hz, 1H, Ar*H*), 7.05 (t, *J* = 7.9 Hz, 1H, Ar*H*), 5.13 (s, 2H, C*H*$_2$), 3.95 (s, 3H, C*H*$_3$). **^{13}C NMR** (151 MHz, $CDCl_3$) δ 166.52 (C=O), 143.98 (ArCH), 140.59 (ArC), 137.13 (ArC), 131.24 (ArCH), 129.72 (ArCH), 103.61 (ArC), 52.72 (CH_3), 36.20 (CH_2).

The spectroscopic data are in good agreement with those reported in literature.[16]

3-(4-Iodo-1-oxoisoindolin-2-yl)piperidine-2,6-dione (9)

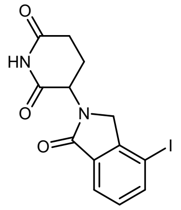

Bromide **8** (0.300 g, 0.84 mmol), 3-aminopiperidine-2,6-dione hydrochloride (0.209 g, 1.27 mmol) and DIPEA (0.74 mmol, 4.23 mmol) were dissolved in acetonitrile (3.5 mL). The solution was refluxed for 7 h. After the reaction was over, the resulting mixture was concentrated *in vacuo*. The crude product was partitioned with EtOAc (10 mL) and water (10 mL). The precipitated solid was collected by filtration and washed with water (10 mL). 3-(4-iodo-1-oxoisoindolin-2-yl)piperidine-2,6-dione **9** was collected as a purple solid (0.246 g, 79%). **R_f** (70 % EtOAc in Petroleum Ether 40-60) = 0.63. **^{1}H NMR** (601 MHz, DMSO-d_6) δ 11.00 (s, 1H, N*H*), 8.04 (d, *J* = 7.6 Hz, 1H, Ar*H*), 7.77 (d, *J* = 7.6 Hz, 1H, Ar*H*), 7.35 (t, *J* = 7.6 Hz, 1H, Ar*H*), 5.14 (dd, *J* = 13.3, 5.1 Hz, 1H, C*H*), 4.28 (d, *J* = 17.5 Hz, 1H, CH_AH_B), 4.14 (d, *J* = 17.5 Hz, 1H, CH_AH_B), 2.95 - 2.87 (m, 1H, CH_CH_D), 2.65 – 2.58 (m, 1H, CH_CH_D), 2.50 - 2.45 (m, 1H, CH_EH_F), 2.05 – 2.00 (m, 1H, CH_EH_F). **^{13}C NMR** (151 MHz, DMSO-d_6) δ 182.82 (C=O), 170.87 (C=O), 167.62 (C=O), 146.37 (ArCH), 140.49 (ArC), 133.28 (ArC), 130.23 (ArCH), 122.89 (ArCH), 91.62 (ArC), 51.69 (CH), 51.11 (CH_2), 31.18 (CH_2), 22.26 (CH_2).

The spectroscopic data are in good agreement with those reported in literature.[17]

3-(1-Oxo-4-{2-[tris(propan-2-yl)silyl]ethynyl}-2,3-dihydro-1*H*-isoindol-2-yl)piperidine-2,6-dione (10)

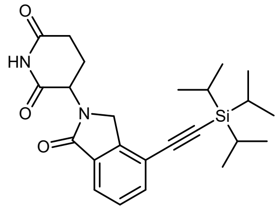

To a solution of 3-(4-iodo-1-oxoisoindolin-2-yl)piperidine-2,6-dione **9** (0.246 g, 0.66 mmol) and TIPS-acetylene (0.363 g, 1.99 mmol) in anhydrous DMF (6 mL), triethylamine (0.28 mL, 1.99 mmol) was added. $Pd(PPh_3)_2Cl_2$ (0.019 g, 0.03 mmol) and CuI (5.0 mg g, 0.03 mmol) were added and the mixture was stirred at 80 °C for 24 h under nitrogen. After completion of the reaction, as indicated by TLC, the mixture was diluted with ice-cold EtOAc (25 mL) and filtered through celite. The organic residue was concentrated under reduced pressure, and the product was purified using flash column chromatography (45 % EtOAc in Petroleum Ether 40-60) to give the alkyne **10** as a white powder (0.177 g, 63%). **R_f** (70 % EtOAc in Petroleum Ether 40-60) = 0.82. **^{1}H NMR** (601 MHz, DMSO-d_6) δ 10.99 (s, 1H, N*H*), 7.77 (d, *J* = 7.5 Hz, 1H, Ar*H*), 7.72 (d, *J* = 7.5 Hz, 1H, Ar*H*), 7.56 (t, *J* = 7.5 Hz, 1H, Ar*H*), 5.11 (dd, *J* = 13.3, 5.1 Hz, 1H, C*H*), 4.45 (d, *J* = 17.5 Hz, 1H, CH_AH_B), 4.36 (d, *J* = 17.5 Hz, 1H, CH_AH_B), 2.92 - 2.86 (m, 1H, CH_CH_D), 2.63 – 2.59 (m, 1H, CH_CH_D), 2.45 - 2.40 (m, 1H, CH_EH_F), 2.07 – 2.03 (m, 1H, CH_EH_F), 1.12 – 1.11 (m, 21H, 3C*H* and 6CH_3). **^{13}C NMR** (151 MHz, DMSO-d_6) δ 172.84 (C=O), 170.95 (C=O), 167.41 (C=O), 144.05 (ArC), 134.63 (ArCH), 132.21 (ArC), 128.78 (ArCH), 123.63 (ArCH), 117.84 (ArC), 102.37 (C), 95.90 (C), 51.89 (CH), 47.17 (CH_2), 31.19 (CH_2), 22.75 (CH_2), 18.48 (6CH_3), 10.58 (3CH). **HRMS** (ESI) $[M+Na]^+$ found 447.2074, $C_{24}H_{33}N_2O_3SiNa$ requires 447.2080.

3-(4-Ethynyl-1-oxo-2,3-dihydro-1*H*-isoindol-2-yl)piperidine-2,6-dione (11)

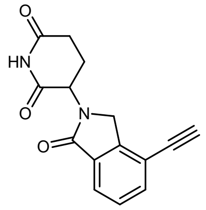

To a solution of alkyne **10** (31 mg, 0.07 mmol) in DMF (0.70 mL) was added tetrabutylammonium fluoride (0.03 mL, 0.10 mmol) dropwise. The mixture was stirred for 4 h at room temperature. After completion of the reaction, as indicated by TLC, the mixture was diluted with EtOAc (20 mL) and washed with NH_4Cl (3 × 20 mL). The organic residue was dried over $MgSO_4$ and concentrated *in vacuo*, to give the terminal alkyne **11** as a white powder (21.0 mg, 47%) which was used for the next reaction without further purification. **R_f** (70 % EtOAc in Petroleum Ether 40-60) = 0.52. **^{1}H NMR** (601 MHz, DMSO-d_6) δ 10.99 (s, 1H, N*H*), 7.78 (d, *J* = 7.5 Hz, 1H, Ar*H*), 7.74 (d, *J* = 7.5 Hz, 1H, Ar*H*), 7.56

(t, *J* = 7.5 Hz, 1H, Ar*H*), 5.13 (dd, *J* = 13.3, 5.1 Hz, 1H, C*H*), 4.60 (s, 1H, C*H*), 4.50 (d, *J* = 17.7 Hz, 1H, C$H_A$$H_B$), 4.35 (d, *J* = 17.7 Hz, 1H, C$H_A$$H_B$), 2.94-2.88 (m, 1H, C$H_C$$H_D$), 2.61 – 2.56 (m, 1H, C$H_C$$H_D$), 2.48 – 2.43 (m, 1H, C$H_E$$H_F$), 2.03 – 1.99 (m, 1H, C$H_E$$H_F$). **^{13}C NMR** (151 MHz, DMSO-d_6) δ 172.87 (C=O), 170.94 (C=O), 167.48 (C=O), 144.38 (ArC), 134.66 (ArCH), 132.12 (ArC), 128.74 (ArCH), 123.71 (ArCH), 117.30 (ArC), 86.07 (C), 79.30 (CH), 51.69 (CH), 46.95 (CH_2), 31.21 (CH_2), 22.32 (CH_2). **HRMS** (ESI) $[M+Na]^+$ found 291.0740, $C_{15}H_{13}N_2O_3Na$ requires 291.0746.

3-[4-(2-Bromoethynyl)-1-oxo-2,3-dihydro-1*H*-isoindol-2-yl]piperidine-2,6-dione (12)

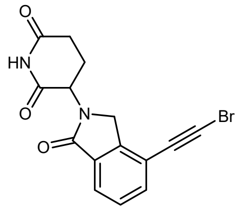

To a solution of terminal alkyne **11** (21.0 mg, 0.08 mmol) in DMF (0.75 mL) was added *N*-bromosuccinimide (0.092 g, 0.1 mmol) and $AgNO_3$ (1.0 mg, 0.01 mmol). The mixture was stirred for 2 h in room temperature. After completion of the reaction, as indicated by TLC, the precipitate was removed by filtration and the filtrate was diluted with EtOAc (10 mL) and washed with brine (3 × 10 mL). The organic fraction was dried over $MgSO_4$ and concentrated *in vacuo*. The crude product was purified by flash column chromatography (60% EtOAc in petroleum ether 40-60) to give the brominated alkyne **12** as a white powder (16 mg, 59%). **R_f** (60% EtOAc in petroleum ether 40-60) = 0.41. **^{1}H NMR** (500 MHz, DMSO-d_6) δ 10.99 (s, 1H, N*H*), 7.78 (d, *J* = 7.7 Hz, 1H, Ar*H*), 7.75 (d, *J* = 7.7 Hz, 1H, Ar*H*), 7.56 (t, *J* = 7.7 Hz, 1H, Ar*H*), 5.14 (dd, *J* = 13.3, 5.2 Hz, 1H, C*H*), 4.52 (d, *J* = 17.8 Hz, 1H, C$H_A$$H_B$), 4.35 (d, *J* = 17.8 Hz, 1H, C$H_A$$H_B$), 2.95 – 2.87 (m, 1H, C$H_C$$H_D$), 2.62 – 2.56 (m, 1H, C$H_C$$H_D$), 2.48 – 2.43 (m, 1H, C$H_E$$H_F$), 2.03 – 1.97 (m, 1H, C$H_E$$H_F$). **^{13}C NMR** (126 MHz, DMSO-d_6) δ 172.82 (C=O), 170.89 (C=O), 167.36 (C=O), 144.52 (ArC), 134.72 (ArCH), 132.13 (ArC), 128.70 (ArCH), 123.70 (ArCH), 117.46 (ArC), 75.67 (C), 58.34 (C), 51.60 (CH), 46.83 (CH_2), 31.16 (CH_2), 22.29 (CH_2).

Scheme S3. Synthesis of **19**.

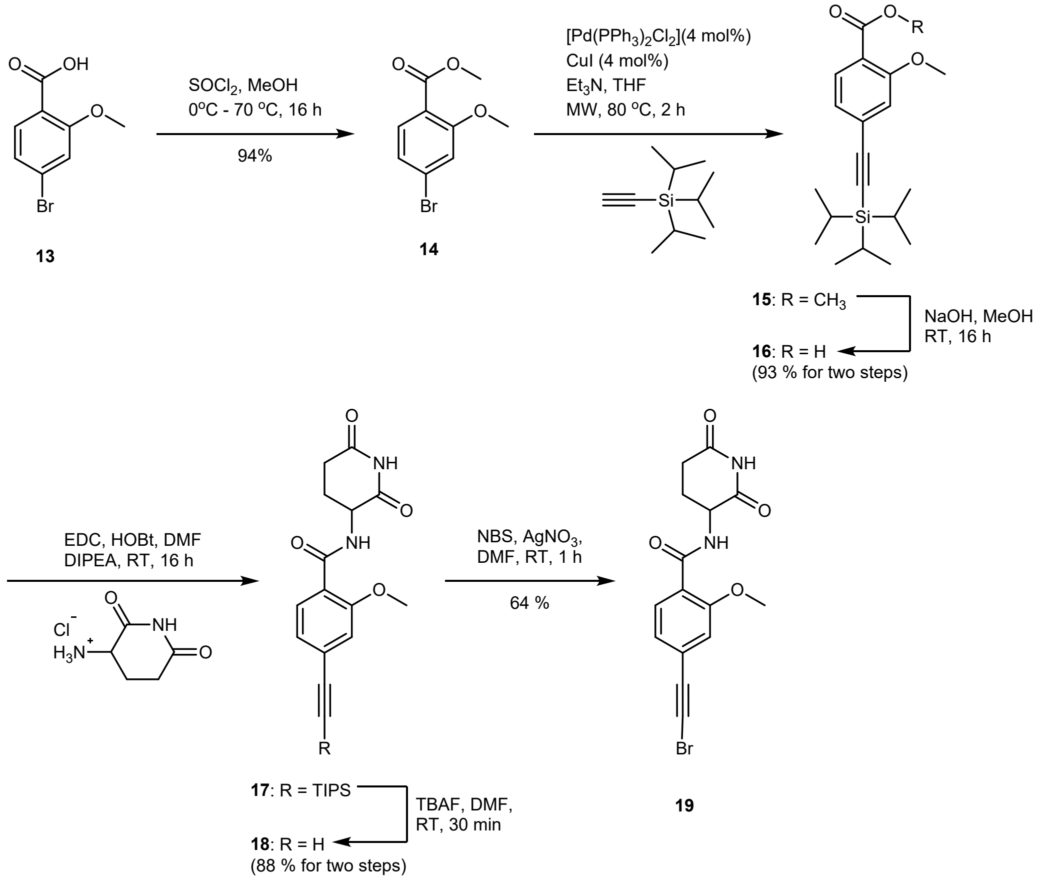

Methyl 4-bromo-2-methoxybenzoate (14)

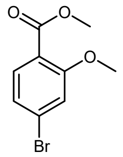

To a solution of 4-bromo-2-methoxybenzoic acid **13** (1.5 g, 6.5 mmol) in MeOH (30 mL) was slowly added $SOCl_2$ (0.70 mL, 9.7 mmol) at 0 °C. The mixture was refluxed at 70 °C for 16 h. After the reaction was finished, the solvent was removed *in vacuo* and the resulting residue was redissolved in EtOAc (50 mL), washed with $NaHCO_3$ (50 mL), brine (50 mL), dried over $MgSO_4$, and concentrated under reduced pressure to give the ester **14** as a white solid (1.49 g, 94%). $\mathbf{R_f}$ (10 % EtOAC in Petroleum Ether 40-60) = 0.34. **^{1}H NMR** (500 MHz, $CDCl_3$) δ 7.67 (d, *J* = 8.6 Hz, 1H, Ar*H*), 7.14 – 7.11 (m, 2H, Ar*H*), 3.90 (s, 3H, C*H*$_3$), 3.88 (s, 3H, C*H*$_3$). **^{13}C NMR** (126 MHz, $CDCl_3$) δ 166.04 (C=O), 159.84 (ArC), 133.04 (ArCH), 127.85 (ArC), 123.56 (ArCH), 119.03 (ArC), 115.83 (ArCH), 56.45 (CH_3), 52.26 (CH_3).

The spectroscopic data are in good agreement with those reported in literature.[18]

Methyl 2-methoxy-4-{2-[tris(propan-2-yl)silyl]ethynyl}benzoate (15)

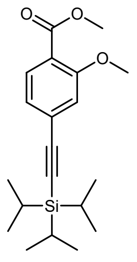

In an oven-dried microwave vial ester **14** (1.00 g, 4.08 mmol) was dissolved in anhydrous THF (12 mL). To the solution $Pd(PPh_3)_2Cl_2$ (0.114 g, 0.16 mmol), CuI (0.031 g, 0.16 mmol), Et_3N (1.70 mL, 12.2 mmol) and TIPS-acetylene (1.49 g, 8.16 mmol) were added and the reaction mixture was stirred at 80 °C for 1 h under microwave conditions. After completion of the reaction, the mixture was diluted with ice-cold EtOAc (25 mL) and filtered through celite. The organic residue was concentrated *in vacuo*. The residue was purified using flash column chromatography (0 to 5 % EtOAc in Petroleum Ether 40-60) to give alkyne **15** as a yellow oil (1.32 g, 93 %). **R_f** (10 % EtOAc in Petroleum Ether 40-60) = 0.44. **^{1}H NMR** (500 MHz, $CDCl_3$) δ 7.73 (d, *J* = 8.0 Hz, 1H, Ar*H*), 7.08 (d, *J* = 8.0 Hz, 1H, Ar*H*), 7.02 (s, 1H, Ar*H*), 3.91 (s, 3H, C*H*$_3$), 3.88 (s, 3H, C*H*$_3$), 1.18 – 1.09 (m, 21H, 3C*H* and 6C*H*$_3$). **^{13}C NMR** (126 MHz, $CDCl_3$) δ 166.24 (C=O), 159.03 (ArC), 131.78 (ArCH), 128.77 (ArC), 124.14 (ArCH), 119.95 (ArC), 115.33 (ArCH), 106.19 (C), 93.85 (C), 56.25 (CH_3), 52.20 (CH_3), 18.79 (6CH_3), 11.42 (3CH). **HRMS** (ESI) $[M+Na]^+$ found 369.1856, $C_{20}H_{31}O_3SiNa$ requires 369.1862.

2-Methoxy-4-{2-[tris(propan-2-yl)silyl]ethynyl}benzoic acid (16)

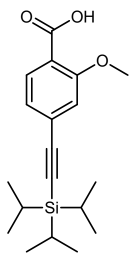

To a solution of alkyne **15** (1.32 g, 3.80 mmol) in MeOH (15 mL) was added sodium hydroxide (0.30 mL, 7.6 mmol, 1 M aq.) and the reaction mixture was stirred at room temperature for 16 h. After completion of the reaction the pH of the mixture was adjusted to 3 with the addition of of HCl (1 M aq.). The reaction mixture was extracted with DCM (3 × 50 mL). The combined organic residues were dried over $MgSO_4$ and concentrated under reduced pressure to give the acid **16** as a yellow solid (1.28 g, 100%). **R_f** (70 % EtOAc in Petroleum Ether 40-60) = 0.12. **^{1}H NMR** (601 MHz, $CDCl_3$) δ 8.11 (d, *J* = 8.0 Hz, 1H, Ar*H*), 7.23 (d, *J* = 8.0 Hz, 1H, Ar*H*), 7.10 (s, 1H, Ar*H*), 4.09 (s, 3H, C*H*$_3$), 1.14 (m, 21H, 3C*H* and 6C*H*$_3$). **^{13}C NMR** (151 MHz, $CDCl_3$) δ 164.89 (C=O), 157.78 (ArC), 133.83 (ArCH), 130.41 (ArC), 126.17 (ArCH), 117.43 (ArC), 114.90 (ArCH), 105.42 (C), 95.87 (C), 57.01 (CH_3), 18.78 (6CH_3), 11.39 (3CH). **HRMS** (ESI) $[M+Na]^+$ found 356.1726, $C_{19}H_{29}O_3SiNa$ requires 356.1784.

***N*-(2,6-dioxopiperidin-3-yl) 2-methoxy-4-{2-[tris(propan-2-yl)silyl]ethynyl}benzamide (17)**

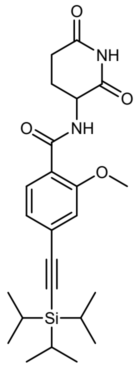

Acid **16** (1.12 g, 3.37 mmol) and 3-aminopiperidine-2,6-dione hydrochloride (2.22 g, 13.5 mmol) were suspended in DMF (15 mL). Subsequently, DIPEA (2.35 mL, 13.5 mmol), EDC (0.575 g, 3.70 mmol) and HOBt · H_2O (0.501 g, 3.70 mmol) were added and the mixture was stirred at room temperature. After 24 h, the reaction was quenched by the addition of semi saturated NH_4Cl solution (40 mL) and then extracted with 10 % MeOH in EtOAc (3 × 60 mL). The combined organic residues were washed with brine (40 mL), dried over $MgSO_4$ and concentrated *in vacuo*. The product was purified using flash column chromatography (0 to 50 % EtOAc in Petroleum Ether 40-60) to give amide **17** as a white solid (1.42 g, 95 %). **R_f** (70 % EtOAc in Petroleum Ether 40-60) = 0.62. **^{1}H NMR** (500 MHz, DMSO-*d*$_6$) δ 10.88 (s, 1H, N*H*), 8.62 (d, *J* = 7.4 Hz, 1H, N*H*), 7.83 (d, *J* = 8.2 Hz, 1H, Ar*H*), 7.17 – 7.14 (m, 2H, Ar*H*), 4.78 - 4.71 (m, 1H, C*H*), 3.95 (s, 3H, C*H*$_3$), 2.82 – 2.72 (m, 1H, C*H*$_A$H$_B$), 2.56 - 2.51 (m, 1H, CH$_A$*H*$_B$), 2.13 – 2.08 (m, 2H, C*H*$_2$), 1.13 – 1.08 (m, 21H, 3C*H* and 6C*H*$_3$). **^{13}C NMR** (126 MHz, DMSO-*d*$_6$) δ 172.89 (C=O), 172.16 (C=O), 163.85 (C=O), 157.12 (ArC), 131.17 (ArCH), 126.30 (ArC), 124.33 (ArCH),

122.33 (ArC), 114.77 (ArCH), 106.34 (C), 92.33 (C), 56.27 (CH_3), 50.04 (CH), 30.92 (CH_2), 24.04 (CH_2), 18.50 (6CH_3), 10.68 (3CH). **HRMS** (ESI) $[M+Na]^+$ found 465.2180, $C_{24}H_{34}N_2O_4SiNa$ requires 465.2186.

***N*-(2,6-dioxopiperidin-3-yl) 4-ethynyl-2-methoxybenzamide (18)**

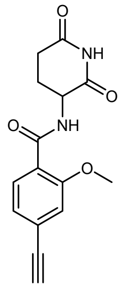

To a solution of amide **17** (50 mg, 0.11 mmol) in DMF (1 mL) was added tetrabutylammonium fluoride (0.04 mL, 0.14 mmol) dropwise. The mixture was stirred for 5 h at room temperature. After completion of the reaction, as indicated by TLC, the mixture was diluted with EtOAc (15 mL) and washed with NH_4Cl (3 × 15 mL). The organic residue was dried over $MgSO_4$ and concentrated *in vacuo*, to give the unprotected terminal alkyne **18** as a white powder (30 mg, 93 %) which was used in the next reaction without further purification. $\mathbf{R_f}$ (EtOAc) = 0.77. **^{1}H NMR** (601 MHz, DMSO-d_6) δ 10.88 (s, 1H, N*H*), 8.62 (d, *J* = 7.6 Hz, 1H, N*H*), 7.82 (d, *J* = 7.9 Hz, 1H, Ar*H*), 7.24 (s, 1H, Ar*H*), 7.17 (d, *J* = 7.9 Hz, 1H, Ar*H*), 4.76 – 4.72 (m, 1H, C*H*), 4.38 (s, 1H, C*H*), 3.93 (s, 3H, CH_3), 2.81 – 2.73 (m, 1H, C$H_A$$H_B$), 2.56 – 2.53 (m, 1H, C$H_A$$H_B$), 2.13 – 2.07 (m, 2H, C$H_2$). **^{13}C NMR** (151 MHz, DMSO-d_6) δ 172.95 (C=O), 172.17 (C=O), 163.97 (C=O), 157.05 (ArC), 131.08 (ArCH), 125.78 (ArC), 124.04 (ArCH), 122.50 (ArC), 115.25 (ArCH), 82.86 (C), 82.80 (C), 56.29 (CH_3), 50.05 (CH), 30.94 (CH_2), 24.07 (CH_2). **HRMS** (ESI) $[M+Na]^+$ found 309.0846, $C_{15}H_{14}N_2O_4Na$ requires 309.0851.

4-(2-Bromoethynyl)-*N*-(2,6-dioxopiperidin-3-yl) 2-methoxybenzamide (19)

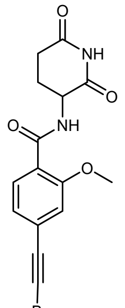

To a solution of terminal alkyne **30** (55.0 mg, 0.19 mmol) in DMF (1.5 mL) was added *N*-bromosuccinimide (44.0 mg, 0.25 mmol) and $AgNO_3$ (3.0 mg, 0.02 mmol). The mixture was stirred for 1 h at room temperature. After completion of the reaction, as indicated by TLC, the precipitate was removed by filtration and the filtrate was diluted with EtOAc (10 mL) and washed with brine (3 × 10 mL). The organic fraction was dried over $MgSO_4$ and concentrated *in vacuo*. The crude product was purified by flash column chromatography (60% EtOAc in petroleum ether 40-60) to give the brominated alkyne **19** as a white powder (0.045 g, 64%). $\mathbf{R_f}$ (60% EtOAc in petroleum ether 40-60) = 0.45. **^{1}H NMR** (601 MHz, DMSO-d_6) δ 10.88 (s, 1H, N*H*), 8.62 (d, *J* = 7.5 Hz, 1H, N*H*), 7.81 (d, *J* = 8.0 Hz, 1H, Ar*H*), 7.28 (s, 1H, Ar*H*), 7.17 (d, *J* = 8.0 Hz, 1H, Ar*H*), 4.76 – 4.72 (m, 1H, C*H*), 3.92 (s, 3H, CH_3), 2.81 – 2.73 (m, 1H, CH_ACH_B), 2.54 – 2.51 (m, 1H, CH_ACH_B), 2.11 – 2.07 (m, 2H, CH_2). **^{13}C NMR** (151 MHz, DMSO-d_6) δ 172.91 (C=O), 172.13 (C=O), 163.91 (C=O), 157.02 (ArC), 131.04 (ArCH), 125.82 (ArC), 124.06 (ArCH), 122.56 (ArC), 115.45 (ArCH), 79.21 (C), 56.31 (CH_3), 55.36 (C), 50.02 (CH), 30.93 (CH_2), 24.04 (CH_2). **HRMS (ESI)** $[^{79}BrM+Na]^+$ found 386.9951, $C_{15}H_{13}{}^{79}BrN_2O_4Na$ requires 386.9956, $[^{81}BrM+Na]^+$ found 388.9930, $C_{15}H_{13}{}^{81}BrN_2O_4Na$ requires 388.9936.

Scheme S4. Synthesis of **LS1** and **LS2**.

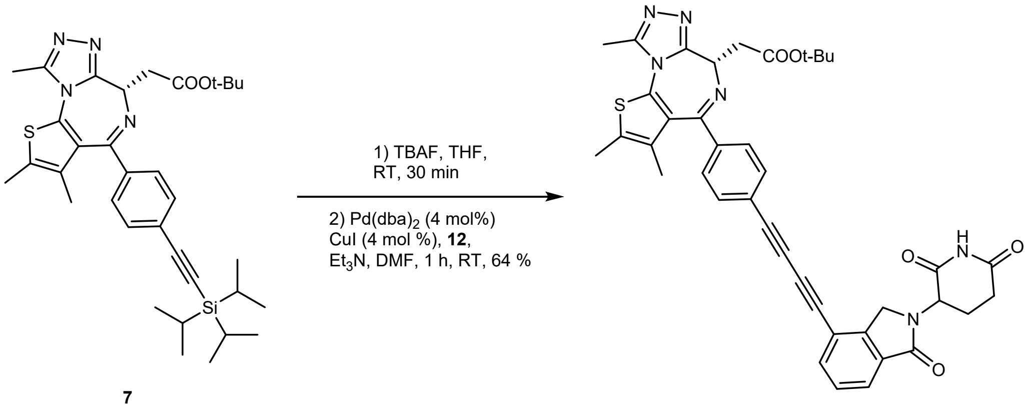

7

LS1

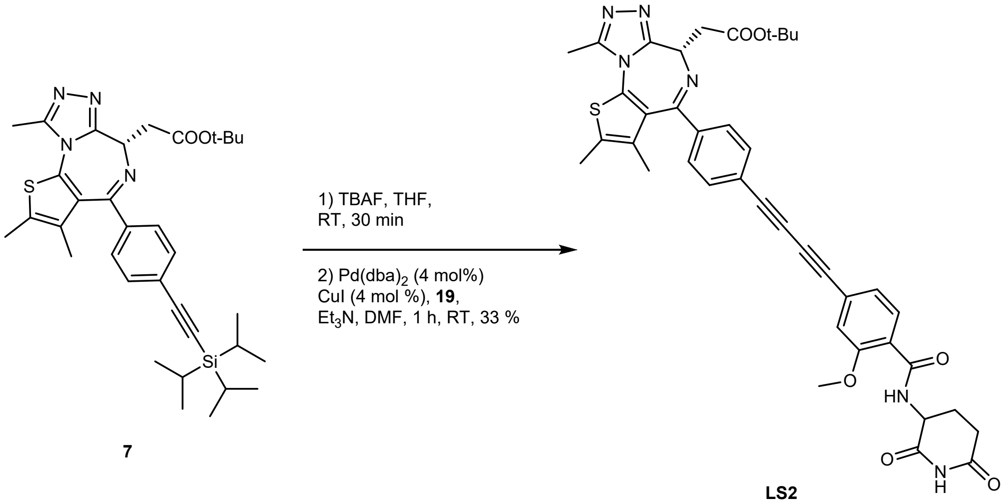

7

LS2

tert-Butyl 2-[(9_S_)-7-(4-{4-[2-(2,6-dioxopiperidin-3-yl)-1-oxo-2,3-dihydro-1_H_-isoindol-4-yl]buta-1,3-diyn-1-yl}phenyl)-4,5,13-trimethyl-3-thia-1,8,11,12-tetraazatricyclo[8.3.0.0^{2,6}] trideca-2(6),4,7,10,12-pentaen-9-yl]acetate (LS1)

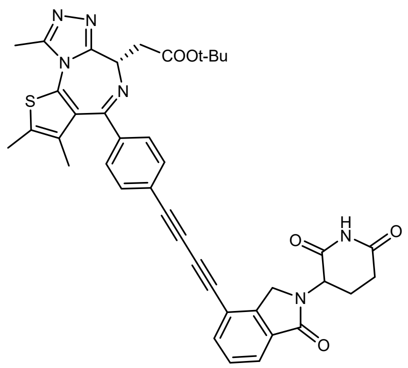

To a solution of TIPS protected alkyne **7** (25.0 mg, 0.04 mmol) in THF (0.4 mL) was added tetrabutylammonium fluoride (0.013 mL, 0.05 mmol) dropwise. The mixture was stirred at room temperature for 30 min. After completion of the reaction, THF was removed *in vacuo.* The residue was dissolved in EtOAc (10 mL) and washed with NH_4Cl (3 × 10 mL, sat. aq.). The organic residue was dried over $MgSO_4$ and concentrated *in vacuo* to give terminal alkyne **20** as a yellow oil (18.0 mg) which was used for the next step without further purification. To a solution of the crude alkyne **20** (18.0 mg, 0.040 mmol) and brominated alkyne **12** (11.0 mg, 0.03 mmol) in anhydrous DMF (0.4 mL), triethylamine (0.01 mL, 0.01 mmol) was added. $Pd(dba)_2$ (1.0 mg, 2.0 µmol) and CuI (0.2 mg, 1.0 µmol) were added and the mixture was stirred at room temperature for 1 h under nitrogen. After completion of the reaction, as indicated by TLC, the mixture was diluted with ice-cold EtOAc (10 mL) and filtered through celite. The organic residue was

concentrated under reduced pressure, and the product was purified using flash column chromatography (0-5 % MeOH in DCM) to give the diyne **LS1** as a yellow solid (11.0 mg, 64 %). **R$_f$** (3 % MeOH in DCM) = 0.26. **IR** (neat, cm^{-1}) 3088 (N-H), 2192 (C≡C), 1699 (C=O), 1592 (C=N), 1148 (C-O). **^{1}H NMR** (500 MHz, DMSO-*d*$_6$) δ 11.00 (s, 1H, N*H*), 7.88 (d, *J* = 7.1 Hz, 1H, Ar*H*), 7.85 (d, *J* = 7.1 Hz, 1H, Ar*H*), 7.68 (d, *J* = 8.2 Hz, 2H, ArH), 7.61 (t, *J* = 7.6 Hz, 1H, Ar*H*), 7.48 (d, *J* = 8.2 Hz, 2H, Ar*H*), 5.16 (dd, *J* = 13.2, 5.3 Hz, 1H, C*H*), 4.59 (d, *J* = 17.4 Hz, 1H, C*H*$_A$H$_B$), 4.46-4.40 (m, 2H, C*H* and CH$_A$*H*$_B$), 3.40-3.36 (m, 2H, C*H*$_2$), 3.13-3.07 (m, 1H, C*H*$_C$H$_D$), 2.95-2.88 (m, 1H, CH$_C$*H*$_D$), 2.60 (s, 3H, C*H*$_3$), 2.49-2.47 (m, 1H, C*H*$_E$H$_F$), 2.42 (s, 3H, C*H*$_3$), 2.03-1.98 (m, 1H, CH$_E$*H*$_F$), 1.64 (s, 3H, C*H*$_3$), 1.43 (s, 9H, C*H*$_3$). **^{13}C NMR** (126 MHz, DMSO-*d*$_6$) δ 172.82 (C=O), 170.85 (C=O), 169.70 (C=O), 167.18 (C=O), 163.32 (ArC), 163.31 (C=N), 145.39 (ArC), 141.07 (ArC), 138.97 (ArC), 135.34 (ArC), 132.58 (2ArCH), 132.42 (ArC), 132.30 (ArC), 130.83 (ArCH), 129.69 (ArC), 129.39 (ArCH), 128.92 (ArCH), 128.71 (2ArCH), 124.68 (ArC), 121.98 (ArC), 115.95 (ArC), 82.69 (C), 80.20 (C), 78.34 (C), 77.64 (C), 74.82 (C), 51.65 (CH), 46.90 (CH), 45.77 (CH$_2$), 37.41 (CH$_2$), 31.15 (CH$_2$), 27.79 (3CH$_3$), 22.29 (CH$_2$), 13.95 (CH$_3$), 12.66 (CH$_3$), 11.27 (CH$_3$). **HRMS** (ESI) [M+Na]$^+$ found 735.2360, $C_{40}H_{36}N_6O_5SNa$ requires 735.2366.

***tert*-Butyl 2-[(9*S*)-7-[4-(4-{4-[(2,6-dioxopiperidin-3-yl)carbamoyl]-3-methoxyphenyl}buta-1,3-diyn-1-yl)phenyl]-4,5,13-trimethyl-3-thia-1,8,11,12-tetraazatricyclo[8.3.0.0^{2,6}]trideca-2 (6),4,7,10,12-pentaen-9-yl]acetate (LS2)**

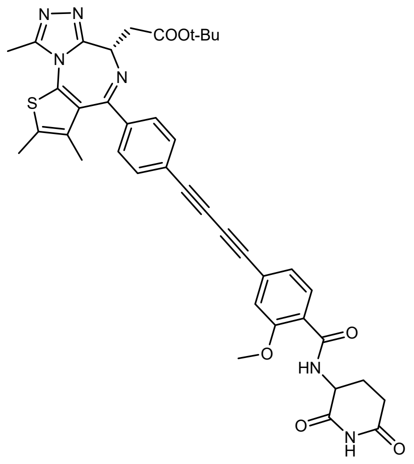

To a solution of TIPS protected alkyne **7** (22.0 mg, 0.040 mmol) in THF (0.36 mL) was added tetrabutylammonium fluoride (0.011 mL, 0.04 mmol) dropwise. The mixture was stirred at room temperature for 30 min. After completion of the reaction, THF was removed *in vacuo.* The residue was dissolved in EtOAc (10 mL) and washed with NH_4Cl (3 × 10 mL, sat. aq.). The organic residue was dried over $MgSO_4$ and concentrated *in vacuo* to give terminal alkyne **20** as a yellow oil (16.0 mg) which was used in the next step without further purification. To a solution of the crude alkyne **20** (16.0 mg, 0.040 mmol) and brominated alkyne **19** (13.0 mg, 0.03 mmol) in anhydrous DMF (0.4 mL), triethylamine (0.01 mL, 0.1 mmol) was added. Pd(dba)$_2$ (1.0 mg, 0.001 mmol) and CuI (0.30 mg, 0.001 mmol) were added and the mixture was stirred at room temperature for 1 h under nitrogen. After completion of the reaction, as indicated by TLC, the mixture was diluted with ice-cold EtOAc (10 mL) and filtered through celite. The organic residue was concentrated under reduced pressure, and the product was purified using flash column chromatography (80-90 % EtOAc in Petroleum Ether 40-60) to give diyne **LS2** as a yellow solid (8.0 mg, 33 %). **R$_f$** (EtOAc) = 0.15. **IR** (neat, cm^{-1}) 3362 (N-H), 3049 (N-H), 2924 (C-H), 2852 (C-H), 1708 (C=O), 1648 (C=O). **^{1}H NMR** (500 MHz, DMSO-*d*$_6$) δ 10.88 (s, 1H, N*H*), 8.64 (d, *J* = 7.5 Hz, 1H, N*H*), 7.83 (d, *J* = 8.3 Hz, 1H, Ar*H*), 7.68 (d, *J* = 8.4 Hz, 2H, Ar*H*), 7.48 (d, *J* = 8.4 Hz, 2H, Ar*H*), 7.41 (s, 1H, Ar*H*), 7.30 (d, *J* = 8.3 Hz, 1H, Ar*H*), 4.77 – 4.72 (m, 1H, C*H*), 4.45 (dd, *J* = 8.2, 6.2 Hz, 1H, C*H*), 3.94 (s, 3H, C*H*$_3$), 3.36 (dd, *J* = 22.8, 6.3 Hz, 1H, C*H*$_A$H$_B$), 3.30 – 3.28 (m, 1H, CH$_A$*H*$_B$), 2.81 – 2.73 (m, 1H, C*H*$_C$H$_D$), 2.60 (s, 3H, C*H*$_3$), 2.55-2.52 (m, 1H, CH$_C$*H*$_D$), 2.43 (s, 3H, C*H*$_3$), 2.12 -2.06 (m, 2H, C*H*$_2$), 1.64 (s, 3H, C*H*$_3$), 1.43 (s, 9H, C*H*$_3$). **^{13}C NMR** (126 MHz, DMSO-*d*$_6$) δ 172.90 (C=O), 172.08 (C=O), 169.71 (C=O), 163.82 (C=O), 163.32 (C=N), 157.02 (ArC), 154.63 (ArC), 149.87 (ArC), 138.92 (ArC), 132.61 (2ArCH), 132.37 (ArC), 131.13 (2ArCH), 130.82 (ArCH), 129.71 (ArCH), 129.41 (ArCH), 128.71 (ArC), 124.71 (ArC), 124.11 (ArC), 123.63 (ArC), 122.12 (ArC), 115.95 (ArC), 82.18 (C), 82.01 (C), 80.21 (C), 74.95 (C), 74.84 (C), 56.39 (CH$_3$), 53.76 (CH),

50.02 (CH), 37.43 (CH_2), 30.91 (CH_2), 27.80 ($3CH_3$), 24.03 (CH_2), 13.98 (CH_3), 12.68 (CH_3), 11.26 (CH_3). **HRMS** (ESI) $[M+Na]^+$ found 753.2466, $C_{40}H_{38}N_6O_6SNa$ requires 753.2471.

Scheme S5. Synthesis of **23** and **25**.

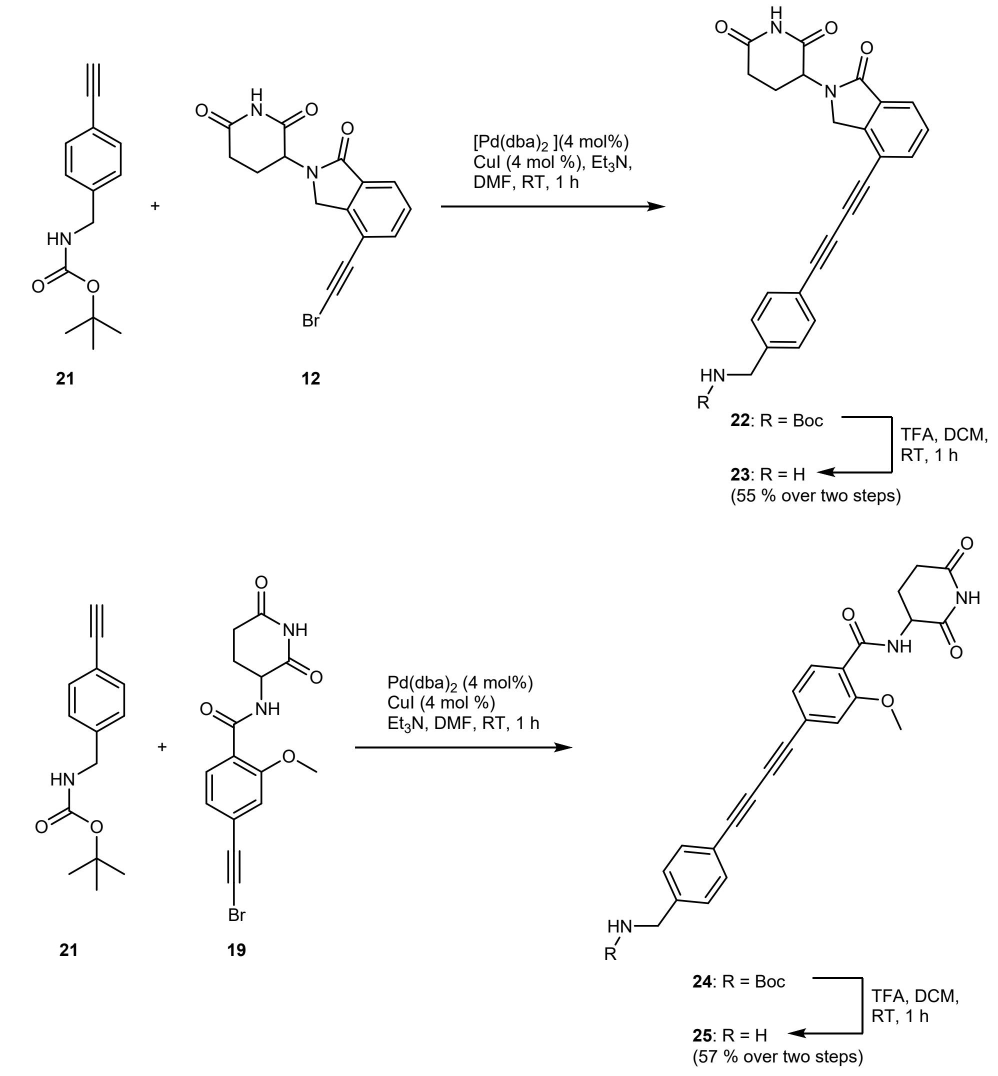

***tert*-Butyl (4-ethynylbenzyl)carbamate (21)**

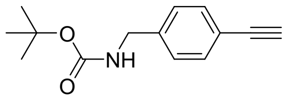

To a suspension of (4-ethynylphenyl)methanamine (0.150 g, 1.14 mmol) in THF (2 mL) was added Boc anhydride (0.898 g, 4.12 mmol). The reaction mixture was heated under reflux at 80 °C. After 16 h, the mixture was concentrated *in vacuo* and the product was purified using flash column chromatography (0-10 %, EtOAc in in Petroleum Ether 40-60) to give carbamate **21** as a white solid (0.110 g, 42%). $\mathbf{R_f}$ (10 % EtOAc in Petroleum Ether 40-60) = 0.44. **^{1}H NMR** (500 MHz, $CDCl_3$) δ 7.45 (d, *J* = 8.2 Hz, 2H, Ar*H*), 7.24 (d, *J* = 8.2 Hz, 2H, Ar*H*), 4.84 (br s, 1H, N*H*), 4.31 (d, *J* = 5.5 Hz, 2H, CH_2), 3.06 (s, 1H, C*H*), 1.46 (s, 9H, 3CH_3). **^{13}C NMR** (151 MHz, $CDCl_3$) δ 156.01 (C=O), 139.98 (ArC), 132.52 (2ArCH), 127.47 (2ArCH), 121.22 (ArC), 83.57 (C), 79.85 (C), 77.31 (CH), 44.55 (CH_2), 28.54 ($3CH_3$).

The spectroscopic data are in good agreement with those reported in literature.[19]

Boc 3-(4-{4-[4-(Aminomethyl)phenyl]buta-1,3-diyn-1-yl}-1-oxo-2,3-dihydro-1*H*-isoindol-2-yl)piperidine-2,6-dione (22)

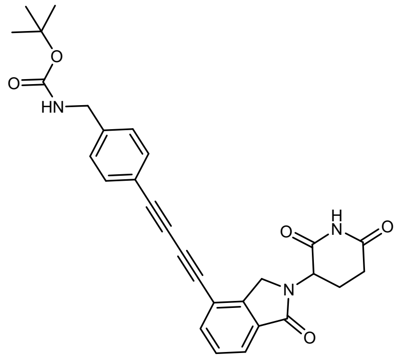

To a solution of brominated alkyne **12** (40.0 mg, 0.02 mmol) and *tert*-butyl (4-ethynylbenzyl)carbamate **21** (15.0 g, 0.05 mmol) in anhydrous DMF (0.35 mL), triethylamine (0.02 mL, 0.1 mmol) was added. $Pd(dba)_2$ (1.0 mg, 2.0 µmol) and CuI (0.3 mg, 2.0 µmol) were then added and the mixture was stirred at room temperature for 1 h. After completion of the reaction, as indicated by TLC, the mixture was diluted with ice-cold EtOAc (10 mL) and filtered through celite. The organic residue was concentrated under reduced pressure, and the product was purified using flash column chromatography (60 % EtOAc in Petroleum Ether 40-60) to give the diyne **22** as a yellow solid (10.0 g, 55%). $\mathbf{R_f}$ (60 % EtOAc in Petroleum Ether 40-60) = 0.33. ^{1}**H NMR** (601 MHz, DMSO-d_6) δ 11.00 (s, 1H, N*H*), 7.87 (d, *J* = 7.7 Hz, 1H, Ar*H*), 7.84 (d, *J* = 7.7 Hz, 1H, Ar*H*), 7.61 (t, *J* = 7.7 Hz, 1H, Ar*H*), 7.59 (d, *J* = 8.0 Hz, 2H, ArH), 7.45 (t, *J* = 6.2 Hz, 1H, N*H*), 7.30 (d, *J* = 8.0 Hz, 2H, Ar*H*), 5.16 (dd, *J* = 13.3, 5.2 Hz, 1H, C*H*), 4.60 (d, *J* = 17.9 Hz, 1H, CH_AH_B (*H*$_A$)), 4.42 (d, *J* = 17.9 Hz, 1H, CH_AH_B (*H*$_B$)), 4.16 (d, *J* = 6.2 Hz, 2H, CH_2), 2.95 -2.89 (m, 1H, CH_CH_D (*H*$_C$)), 2.62-2.58 (m, 1H, CH_CH_D (*H*$_D$)), 2.49-2.47 (m, 1H, CH_EH_F (*H*$_E$)), 2.03-1.99 (m, 1H, CH_EH_F (*H*$_F$)), 1.39 (s, 9H, 3C*H*$_3$). ^{13}**C NMR** (151 MHz, DMSO-d_6) δ 172.82 (C=O), 170.86 (C=O), 167.71 (C=O), 155.80 (C=O), 145.28 (ArC), 135.30 (ArC), 132.47 (2ArCH), 132.24 (ArCH), 128.89 (ArCH), 127.38 (2ArCH), 124.49 (ArCH), 118.19 (ArC), 116.18 (ArC), 91.01 (C), 83.45 (C), 77.96 (C), 77.29 (C), 72.84 (C), 51.64 (CH), 46.92 (CH_2), 43.16 (CH_2), 31.17 (CH_2), 28.21 ($3CH_3$), 22.29 (CH_2). **HRMS** (ESI) $[M+Na]^+$ found 520.1843, $C_{29}H_{27}N_3O_5Na$ requires 520.1848.

3-(4-{4-[4-(Aminomethyl)phenyl]buta-1,3-diyn-1-yl}-1-oxo-2,3-dihydro-1*H*-isoindol-2-yl)piperidine-2,6-dione (23)

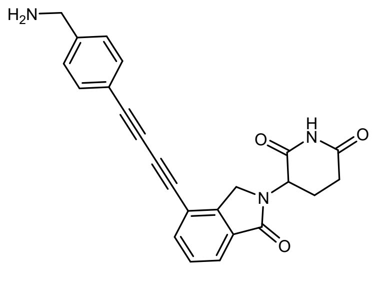

Diyne **22** (10.0 mg, 0.02 mmol) was dissolved in 20% TFA/DCM solution (0.2 mL) and stirred at room temperature for 1 h. The mixture was dried under nitrogen and to the residue was added a 1:1 mixture of DCM/Et_2O (5 mL). The solvent was removed under reduced pressure and the sequence repeated four times to give the trifluoroacetate salt of amine **23** as a white solid (10.03 mg, quant.), quant which was used in the next reaction without further purification. $\mathbf{R_f}$ (10 % MeOH in DCM) = 0.27.

Boc *N*-(2,6-dioxopiperidin-3-yl)
4-{4-[4-(Aminomethyl)phenyl]buta-1,3-diyn-1-yl})-2-methoxybenzamide (24)

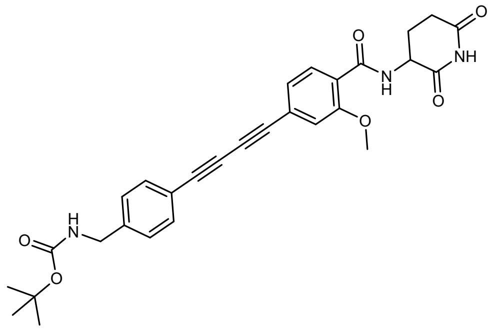

To a solution of brominated alkyne **19** (8.0 mg, 0.02 mmol) and *tert*-butyl (4-ethynylbenzyl)carbamate **21** (15.0 mg, 0.05 mmol) in anhydrous DMF (0.2 mL), was added triethylamine (0.01 mL, 0.06 mmol). $Pd(dba)_2$ (0.5 mg, 0.001 mmol) and CuI (0.2 mg, 0.001 mmol) were then added and the mixture was stirred at room temperature for 1 h. After completion of the reaction, as indicated by TLC, the mixture was diluted with ice-cold EtOAc (10 mL) and filtered through celite. The organic residue was concentrated under reduced pressure, and the product was purified using flash column chromatography (60 % EtOAc in Petroleum Ether 40-60) to give the diyne **36** as a yellow solid (7.0 mg, 57%). $\mathbf{R_f}$ (60 % EtOAc in Petroleum Ether 40-60) = 0.37. **^{1}H NMR** (601 MHz, DMSO-d_6) δ 10.88 (s, 1H, N*H*), 8.64 (d, *J* = 7.5 Hz, 1H, N*H*), 7.83 (d, *J* = 7.9 Hz, 1H, Ar*H*), 7.58 (d, *J* = 8.0 Hz, 2H, Ar*H*), 7.45 (t, *J* = 6.2 Hz, 1H, N*H*), 7.41 (s, 1H, Ar*H*), 7.31 – 7.28 (m, 3H, Ar*H*), 4.77 – 4.72 (m, 1H, C*H*), 4.16 (d, *J* = 6.2 Hz, 2H, CH_2), 3.94 (s, 3H, CH_3), 2.81-2.73 (m, 1H, CH_ACH_B), 2.55-2.51 (m, 1H, CH_ACH_B), 2.12-2.08 (m, 2H, CH_2), 1.40 (s, 9H, 3CH_3). **^{13}C NMR** (151 MHz, DMSO-d_6) δ 172.91 (C=O), 172.10 (C=O), 163.85 (C=O), 157.03 (ArC), 155.81 (C=O), 132.49 (2ArCH), 131.12 (ArCH), 127.37 (2ArCH), 124.62 (ArC), 124.39 (ArCH), 124.10 (ArC), 123.42 (ArC), 118.35 (ArCH), 115.91 (ArC), 82.95 (C), 80.96 (C), 77.97 (C), 75.19 (C), 72.96 (C), 56.38 (CH_3), 50.03 (CH), 43.18 (CH_2), 28.97 (CH_2), 28.22 (3CH_3), 24.04 (CH_2).

***N*-(2,6-dioxopiperidin-3-yl)**
4-{4-[4-(Aminomethyl)phenyl]buta-1,3-diyn-1-yl})-2-methoxybenzamide (25)

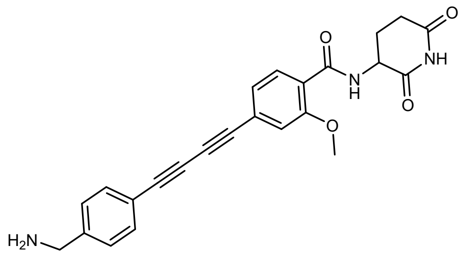

Diyne **24** (6.0 mg, 0.01 mmol) was dissolved in 20% TFA/DCM solution (0.2 mL) and stirred at room temperature for 1 h. The mixture was dried under nitrogen and to the residue was added a 1:1 mixture of DCM/Et_2O (5 mL). The solvent was removed under reduced pressure and the sequence repeated four times to give the trifluoroacetate salt of amine **24** as a white solid (6.0 mg, quant.) which was used to the next reaction without further purification.

Scheme S6. Synthesis of **LS3** and **LS4**.

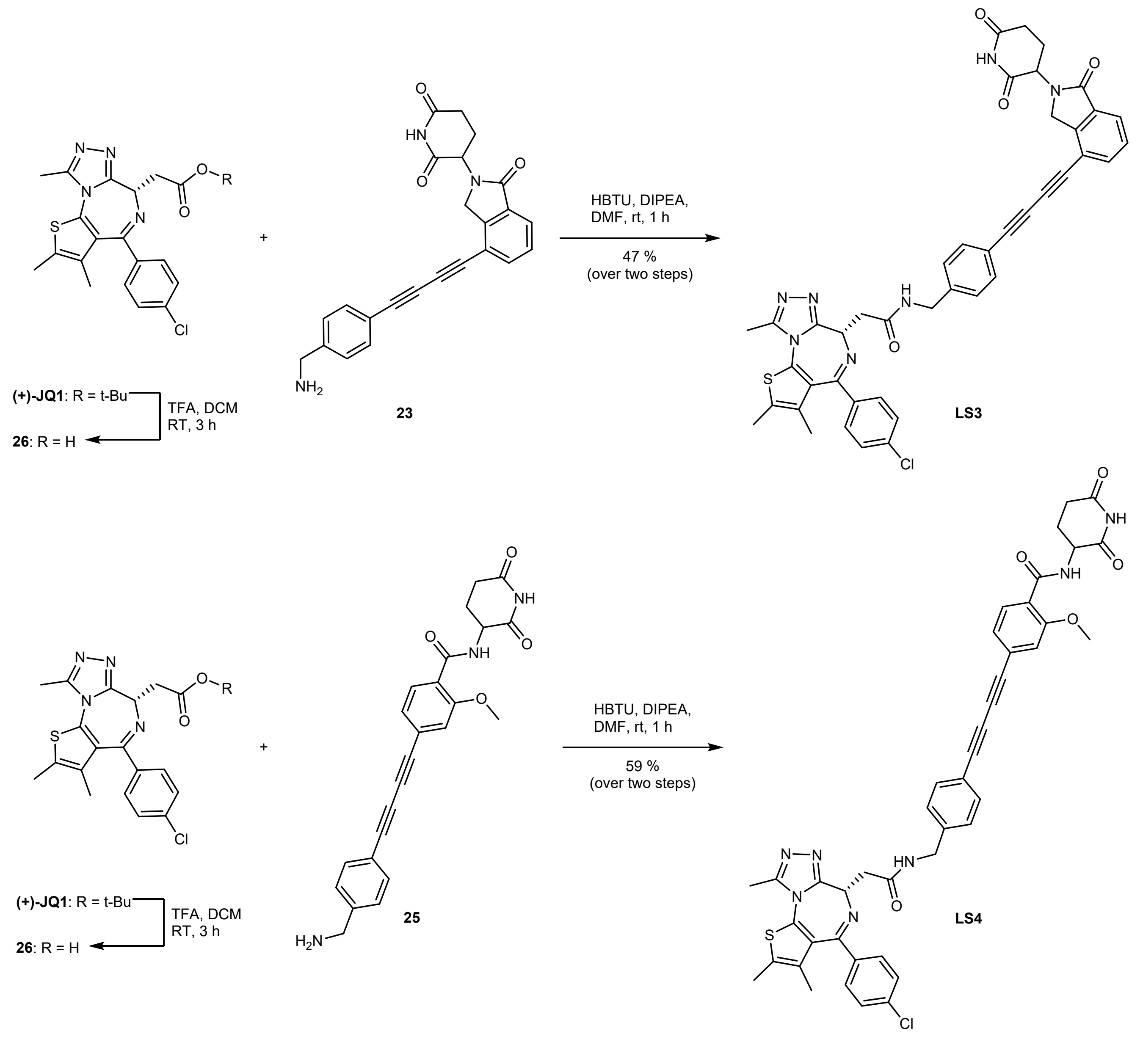

2-[(9*S*)-7-(4-Chlorophenyl)-4,5,13-trimethyl1-3-thioa-1,8,11,12-tetraazatricyclo[8.3.0.0^{2,6}]trideca-2(6),4,7,10,12-pentaen-9-yl]acetic acid (26)

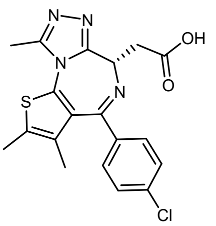

(+)-JQ1 (20.0 mg, 0.06 mmol) was dissolved in 60% TFA/DCM solution (0.2 mL) and stirred at room temperature for 3 h. The mixture was dried under nitrogen and a 1:1 mixture of DCM/Et_2O (5 mL) was added to the residue. The solvent was removed under reduced pressure and the sequence repeated four times to give (+)-JQ1-OH **26** as a yellow solid (17.2 mg, quant.) which was used to the next reaction without further purification. **R_f** (10 % MeOH in DCM) = 0.23.

2-[(9*S*)-7-(4-Chlorophenyl)-4,5,13-trimethyl-3-thia-1,8,11,12-tetraazatricyclo[8.3.0.0^{2,6}] trideca-2(6),4,7,10,12-pentaen-9-yl]-*N*-[(4-{4-[2-(2,6-dioxopiperidin-3-yl)-1-oxo-2,3-dihydro-1*H*-isoindol-4-yl]buta-1,3-diyn-1-yl}phenyl)methyl]acetamide (LS3)

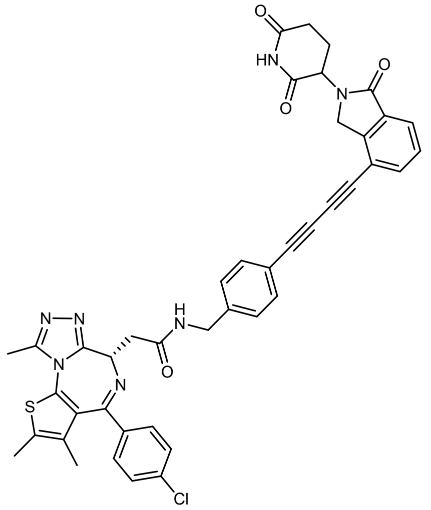

To a solution of JQ1-OH **26** (8.0 mg, 0.02 mmol) in DMF (0.3 mL) was added HBTU (10 mg, 0.03 mmol) and DIPEA (0.013 mL, 0.08 mmol). After 10 min, amine **23** (10.0 mg, 0.02 mmol) was added and the mixture was stirred at room temperature for 1 h. After completion of the reaction, as indicated by TLC, the mixture was diluted with EtOAc (10 mL) and was with $NaHCO_3$ (3 × 10 mL). The organic residue was dried over $MgSO_4$ and concentrated *in vacuo*. The product was purified using column chromatography (0 - 5 % MeOH in DCM) to give amide **LS3** as a yellow solid (7.0 mg, 47%). **R_f** (5 % MeOH in DCM) = 0.32. **IR** (neat, cm^{-1}) 3285 (N-H), 3037 (N-H), 2248 (C≡C), 2216 (C≡C), 1711 (C=O), 1661 (C=O). **^{1}H NMR** (500 MHz, DMSO-d_6) δ 11.00 (s, 1H, N*H*), 8.81 (t, *J* = 6.1 Hz, 1H, N*H*), 7.87 (d, *J* = 7.6 Hz, 1H, Ar*H*), 7.84 (d, *J* = 7.6 Hz, 1H, Ar*H*), 7.63-7.58 (m, 3H, Ar*H*), 7.47 (d, *J* = 8.5 Hz, 2H, Ar*H*), 7.40 (d, *J* = 8.5 Hz, 2H, Ar*H*), 7.35 (d, *J* = 8.2 Hz, 2H, Ar*H*), 5.16 (dd, *J* = 13.3, 5.1 Hz, 1H, C*H*), 4.60 (d, *J* = 17.9 Hz, 1H, CH_AH_B), 4.54 (dd, *J* = 6.3, 3.1 Hz, 1H, C*H*), 4.44-4.33 (m, 3H, CH_AH_B and CH_2), 3.36 (dd, *J* = 15.2, 7.3 Hz, 1H, CH_CH_D), 3.28 (dd, *J* = 15.2, 7.3 Hz, 1H, CH_CH_D), 2.96-2.88 (m, 1H, CH_EH_F), 2.65-2.60 (m, 1H, CH_EH_F), 2.60 (s, 3H, C*H*$_3$), 2.49-2.45 (m, 1H, CH_GH_H), 2.41 (s, 3H, C*H*$_3$), 2.04-1.99 (m, 1H, CH_GH_H), 1.62 (s, 3H, C*H*$_3$). **^{13}C NMR** (126 MHz, DMSO-d_6) δ 172.82 (C=O), 170.86 (C=O), 169.79 (C=O), 167.22 (C=O), 163.09 (C=N), 155.06 (ArC), 145.28 (ArC), 142.32 (ArC), 136.69 (ArC), 135.31 (ArC), 135.23 (ArC), 132.45 (2ArCH), 132.28 (ArC), 132.27 (ArC), 130.71 (ArC), 130.08 (2ArCH), 129.81 (ArCH), 129.54 (ArCH), 128.90 (ArCH), 128.43 (2ArCH), 127.81 (ArC), 127.75 (2ArCH), 124.50 (ArC), 118.30 (ArC), 116.18 (ArC), 83.42 (C), 77.96 (C), 77.34 (C), 72.96 (C), 53.92 (CH), 51.65 (CH), 46.92 (CH_2), 41.87 (CH_2), 37.64 (CH_2), 31.17 (CH_2), 22.31 (CH_2), 14.05 (CH_3), 12.66 (CH_3), 11.29 (CH_3). **HRMS** (ESI) $[M+Na]^+$ found 802.1974, $C_{43}H_{34}ClN_7O_4SNa$ requires 802.1979.

4-{4-[4-({2-[(9*S*)-7-(4-Chlorophenyl)-4,5,13-trimethyl-3-thia-1,8,11,12-tetraazatricyclo [8.3.0.0^{2,6}] trideca-2(6),4,7,10,12-pentaen-9-yl]acetamido}methyl) phenyl]buta-1,3-diyn-1-yl}-N-(2,6-dioxopiperidin-3-yl) -2-methoxybenzamide (LS4)

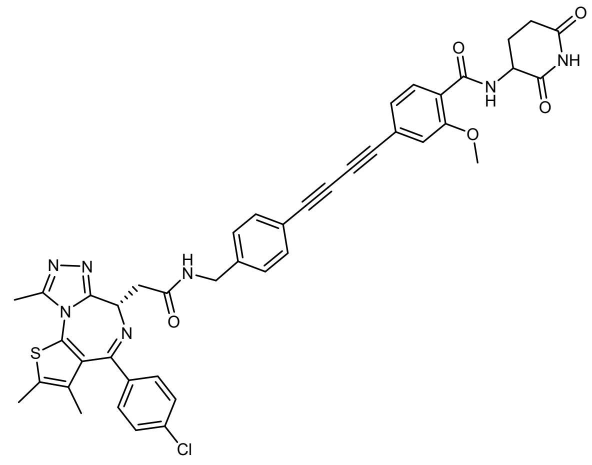

To a solution of JQ1-OH **26** (8.0 mg, 0.02 mmol) in DMF (0.2 mL) was added HBTU (6.0 g, 0.02 mmol) and DIPEA (0.010 mL, 0.05 mmol). After 10 min, amine **25** (6.0 mg, 0.01 mmol) was added and the mixture was stirred at room temperature for 16 h. After completion of the reaction, as indicated by TLC, the mixture was diluted with EtOAc (10 mL) and was with $NaHCO_3$ (3 × 10 mL). The organic residue was dried over $MgSO_4$ and concentrated *in vacuo*. The product was purified using flash column chromatography (0 - 5 % MeOH in DCM) to give amide **LS4** as a yellow solid (7.0 mg, 47%). **R_f** (5 % MeOH in DCM) = 0.36. **IR** (neat, cm^{-1}) 3362 (N-H), 3049 (N-H), 1724 (C=O), 1698 (C=O), 1641 (C=O). **^{1}H NMR** (601 MHz, DMSO-d_6)

δ 10.88 (s, 1H, N*H*), 8.81 (t, *J* = 6.1 Hz, 1H, N*H*), 8.64 (d, *J* = 7.5 Hz, 1H, N*H*), 7.83 (d, *J* = 8.0 Hz, 1H, Ar*H*), 7.58 (d, *J* = 8.1 Hz, 2H, Ar*H*), 7.48 (d, *J* = 8.8 Hz, 2H, Ar*H*), 7.42-7.38 (m, 3H, Ar*H*), 7.35 (d, *J* = 8.8 Hz, 2H, Ar*H*), 7.29 (d, *J* = 8.0 Hz, 1H, Ar*H*), 4.77-4.73 (m, 1H, C*H*), 4.54 (dd, *J* = 8.5, 5.9 Hz, 1H, C*H*), 4.42 (dd, *J* = 15.8, 6.1 Hz, 1H, CH_AH_B), 4.35 (dd, *J* = 15.8, 6.1 Hz, 1H, CH_AH_B), 3.94 (s, 3H, CH_3), 3.36 (dd, *J* = 15.1, 8.5 Hz, 1H, CH_CH_D), 3.28 (dd, *J* = 15.1, 8.5 Hz, 1H, CH_CH_D), 2.81-2.75 (m, 1H, CH_EH_F), 2.60 (s, 3H, CH_3), 2.55-2.51 (m, 1H, CH_EH_F), 2.41 (s, 3H, CH_3), 2.12-2.08 (m, 2H, CH_2), 1.62 (s, 3H, CH_3). **^{13}C NMR** (151 MHz, DMSO-d_6) δ 172.90 (C=O), 172.09 (C=O), 169.78 (C=O), 163.84 (C=N), 163.09 (C=O), 157.02 (ArC), 155.04 (ArC), 142.23 (ArC), 136.70 (ArC), 135.23 (ArC), 132.46 (2ArCH), 132.27 (ArC), 131.12 (2ArCH), 130.71 (ArC), 130.09 (2ArCH), 129.81 (ArC), 129.55 (ArC), 128.43 (ArCH), 127.73 (2ArCH), 124.61 (ArCH), 124.38 (ArC), 123.45 (ArC), 118.44 (ArC), 115.89 (ArCH), 82.92 (C), 81.02 (C), 75.17 (C), 73.07 (C), 56.38 (CH_3), 53.93 (CH), 50.02 (CH), 41.87 (CH_2), 37.65 (CH_2), 30.91 (CH_2), 24.03 (CH_2), 14.04 (CH_3), 12.67 (CH_3), 11.29 (CH_3). **HRMS** (ESI) $[M+Na]^+$ found 820.2079, $C_{43}H_{36}ClN_7O_5SNa$ requires 820.2085.

***tert*-Butyl 2-[(9*S*)-4,5,13-trimethyl-7-[4-(4-phenylbuta-1,3-diyn-1-yl)phenyl]-3-thia1,8,11,12-tetraazatricyclo[8.3.0.0^{2,6}]trideca-2(6),4,7,10,12-pentaen-9-yl]acetate ((+)-JQ1-*p*BADY)**

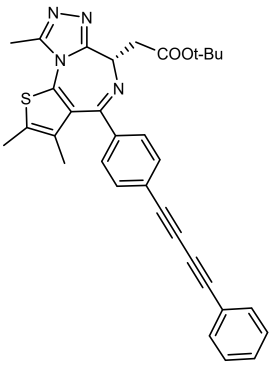

To a solution of alkyne **7** (50.0 mg, 0.08 mmol) in THF (0.8 mL) was added tetrabutylammonium fluoride (26.0 mg, 0.10 mmol) dropwise. The mixture was stirred at room temperature for 30 min. After completion of the reaction, THF was removed *in vacuo.* The residue was dissolved in EtOAc (10 mL) and washed with NH_4Cl (3 × 10 mL, sat. aq.). The organic residue was dried over $MgSO_4$ and concentrated *in vacuo* to give terminal alkyne **20** as a yellow oil (37.0 mg) which was used in the next step without further purification. To a solution of CuCl (8.0 mg, 0.08 mmol) in acetone (0.07 mL) was added tetramethylethylenediamine (0.02 mL, 0.17 mmol) and the reaction was stirred in open atmosphere for 10 min. The crude terminal alkyne **20** (37.0 mg, 0.08 mmol) and phenyl acetylene (25.0 mg, 0.25 mmol) were dissolved in DCM (0.2 mL) and added slowly to the copper solution. The reaction was stirred for 2 h at room temperature. After completion of the reaction, the reaction mixture was concentrated *in vacuo*. The residue was dissolved in DCM (10 mL) and partitioned with NH_4Cl (30 mL, sat. aq.). The aqueous layer was extracted with DCM (2 × 10 mL). The combined organic residues were washed with brine (15 mL), dried over $MgSO_4$ and concentrated *in vacuo*. The residue was purified using column chromatography (0 to 5 % MeOH in DCM) to give a yellow solid which was then redissolved in DCM (1 mL). Quadrapure®IDA resin (100 mg) was added and the solution was agitated for 16 h. The solution was concentrated *in vacuo* to give **(+)-JQ1-*p*BADY** as a yellow solid (40.0 mg, 30%). **R_f** (3 % MeOH in DCM) = 0.35. **IR** (neat, cm^{-1}) 2216 (C≡C), 1725 (C=O), 1591 (C=N). **^{1}H NMR** (500 MHz, $CDCl_3$) δ 7.53-7.50 (m, 4H, Ar*H*), 7.45 (d, *J* = 8.0 Hz, 2H, Ar*H*), 7.38-7.33 (m, 3H, Ar*H*), 4.58 (dd, *J* = 7.7, 6.3 Hz, 1H, C*H*), 3.56 (dd, *J* = 16.9, 7.0 Hz, 1H, CH_AH_B), 3.54 (dd, *J* = 16.9, 7.0 Hz, 1H, CH_AH_B), 2.67 (s, 3H, CH_3), 2.41 (s, 3H, CH_3), 1.69 (s, 3H, CH_3), 1.50 (s, 9H, CH_3). **^{13}C NMR** (126 MHz, $CDCl_3$) δ 170.98 (C=O), 164.04 (C=N), 138.80 (ArC), 137.41 (ArC), 132.69 (2ArCH), 132.67 (2ArCH), 131.01 (ArC), 130.84 (ArC), 130.60 (ArC), 129.56 (ArCH), 128.90 (ArC), 128.80 (ArC), 128.67 (2ArCH), 124.15 (ArC), 121.73 (ArC), 82.81 (C), 81.10 (C), 80.99 (C), 76.08 (C), 73.83 (C), 54.20 (CH), 38.00 (CH_2), 28.33 ($3CH_3$), 14.54 (CH_3), 13.24 (CH_3), 12.06 (CH_3). **HRMS** (ESI) $[M+Na]^+$ found 569.1982, $C_{33}H_{30}N_4O_2SNa$ requires 569.1987.

3-[1-Oxo-4-(4-phenylbuta-1,3-diyn-1-yl)-2,3-dihydro-1*H*-isoindol-2-yl]piperidine-2,6-dione (Lenalidomide-BADY)

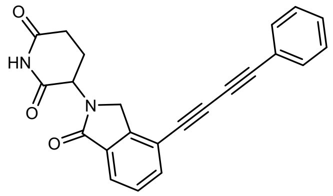

To a solution of terminal alkyne **11** (6.0 mg, 0.02 mmol) and iodoethynyl(benzene) (9.0 mg, 0.03 mmol) in anhydrous DMF (0.3 mL), triethylamine (0.01 mL, 0.06 mmol) was added. $Pd(dba)_2$ (0.5 mg, 1.0 µmol) and CuI (0.1 mg, 1.0 µmol) were added and the mixture was stirred at room temperature for 1 h. After completion of the reaction, as indicated by TLC, the mixture was diluted with ice-cold EtOAc (10 mL) and filtered through celite. The organic residue was concentrated under reduced pressure, and the product was purified using flash column chromatography (60 % EtOAc in Petroleum Ether 40-60) to give the diyne **Lenalidomide-BADY** as an orange solid (4.0 mg, 31%). $\mathbf{R_f}$ (70 % EtOAc in Petroleum Ether 40-60) = 0.51. **IR** (neat, cm^{-1}) 3080 (N-H), 2226 (C≡C), 1705 (C=O), 1662 (C=O), 1612 (C=O). **^{1}H NMR** (601 MHz, DMSO-d_6) δ 11.00 (s, 1H, N*H*), 7.88 (d, *J* = 7.7 Hz, 1H, Ar*H*), 7.85 (d, *J* = 7.7 Hz, 1H, Ar*H*), 7.64 (d, *J* = 7.6 Hz, 2H, Ar*H*), 7.62 (t, *J* = 7.7 HZ, 1H, Ar*H*), 7.53-7.50 (m, 1H, Ar*H*), 7.46 (t, *J* = 7.6 Hz, 2H, Ar*H*), 5.18-5.13 (m, 1H, C*H*), 4.60 (d, *J* = 17.8 Hz, 1H, CH_AH_B with H_A), 4.43 (d, *J* = 17.8 Hz, 1H, CH_AH_B), 2.95-2.88 (m, 1H, $C\mathit{H}_C H_D$), 2.62-2.58 (m, 1H, $CH_C\mathit{H}_D$), 2.49-2.44 (m, 1H, $C\mathit{H}_E H_F$), 2.03-1.98 (m, 1H, $CH_E\mathit{H}_F$). **^{13}C NMR** (151 MHz, DMSO-d_6) δ 172.83 (C=O), 170.87 (C=O), 167.21 (C=O), 145.31 (ArC), 135.35 (ArCH), 132.47 (2ArCH), 130.32 (ArC), 128.90 (2ArCH), 124.54 (ArCH), 122.48 (ArCH), 120.03 (ArC), 116.12 (ArC), 83.31 (C), 77.85 (C), 77.44 (C), 73.09 (C), 51.64 (CH), 46.92 (CH_2), 31.16 (CH_2), 22.29 (CH_2). **HRMS** (ESI) $[M+Na]^+$ found 391.1053, $C_{23}H_{16}N_2O_3Na$ requires 391.1059.

References

1. D. A. Case *et al.*, *J. Chem. Inf. Model.*, 2023, **63**, 6183–6191.
2. J. A. Maier, C. Martinez, K. Kasavajhala, L. Wickstrom, K. E. Hauser and C. Simmerling, *J. Chem. Theory Comput.*, 2015, **11**, 3696–3713.
3. W. L. Jorgensen, J. Chandrasekhar, J. D. Madura, R. W. Impey and M. L. Klein, *J. Chem. Phys.*, 1983, **79**, 926–935.
4. I. S. Joung and T. E. Cheatham, *J. Phys. Chem. B*, 2008, **112**, 9020–9041.
5. I. S. Joung and T. E. Cheatham, *J. Phys. Chem. B*, 2009, **113**, 13279–13290.
6. F. Duarte, P. Bauer, A. Barrozo, B. A. Amrein, M. Purg, J. Åqvist and S. C. L. Kamerlin, *J. Phys. Chem. B*, 2014, **118**, 4351–4362.
7. J. Wang, R. M. Wolf, J. W. Caldwell, P. A. Kollman and D. A. Case, *J. Comput. Chem.*, 2004, **25**, 1157–1174.
8. A. Jakalian, B. L. Bush, D. B. Jack and C. I. Bayly, *J. Comput. Chem.*, 2000, **21**, 132–146.
9. A. Jakalian, D. B. Jack and C. I. Bayly, *J. Comput. Chem.*, 2002, **23**, 1623–1641.
10. T. Darden, D. York and L. Pedersen, *J. Chem. Phys.*, 1993, **98**, 10089–10092.
11. Z. Zhang, X. Liu, K. Yan, M. E. Tuckerman and J. Liu, *J. Phys. Chem. A*, 2019, **123**, 6056–6079.
12. S. Miyamoto and P. A. Kollman, *J. Comput. Chem.*, 1992, **13**, 952–962.
13. J. P. Ryckaert, G. Ciccotti and H. J. C. Berendsen, *J. Comput. Phys.*, 1977, **23**, 327–341.
14. M. Yoneya, H. J. C. Berendsen and K. Hirasawa, *Mol. Simul.*, 1994, **13**, 395–405.
15. P. Filippakopoulos *et al.*, *Nature*, 2010, **468**, 1067–1073.
16. F. Begnini *et al.*, *J. Med. Chem.*, 2022, **65**, 3473–3517.
17. C. Qin *et al.*, *J. Med. Chem.*, 2018, **61**, 6685–6704.
18. S. E. Huff, F. A. Mohammed, M. Yang, P. Agrawal, J. Pink, M. E. Harris, C. G. Dealwis and R. Viswanathan, *J. Med. Chem.*, 2018, **61**, 666–680.
19. W. Wen *et al.*, *J. Med. Chem.*, 2022, **65**, 9126–9143.

LC-MS Chromatograms for LS1-4

LS1

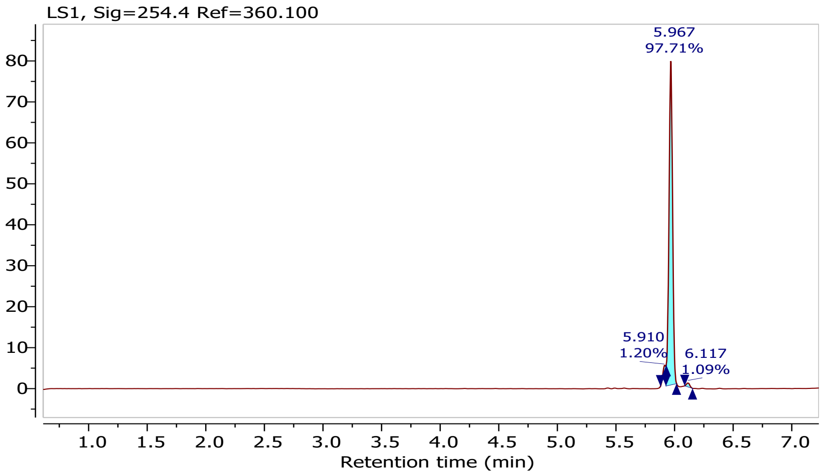

LS1, Sig-254.4 Ref=360.100

Peak #	Retention Time (min)	Type	Height	Area	Total Area %	Start time	End time
1	5.910	BB	1.721	2.10	1.20	5.880	5.930
2	5.967	VB	79.060	96.65	97.71	5.927	6.017
3	6.117	BB	1.017	9.440	1.09	6.087	6.153

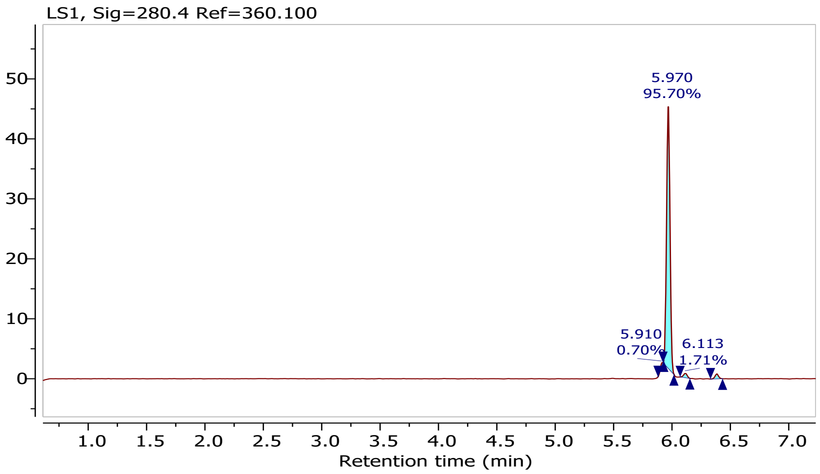

LS1, Sig=280.4 Ref=360.100

Peak #	Retention Time (min)	Type	Height	Area	Total Area %	Start time	End time
1	5.910	BB	0.618	3.302	0.70	5.880	5.930
2	5.970	BB	43.657	448.747	95.70	5.923	6.017
3	6.113	BB	0.751	8.022	1.71	6.070	6.153
4	6.383	BB	0.841	8.822	1.88	6.330	6.433

LS2

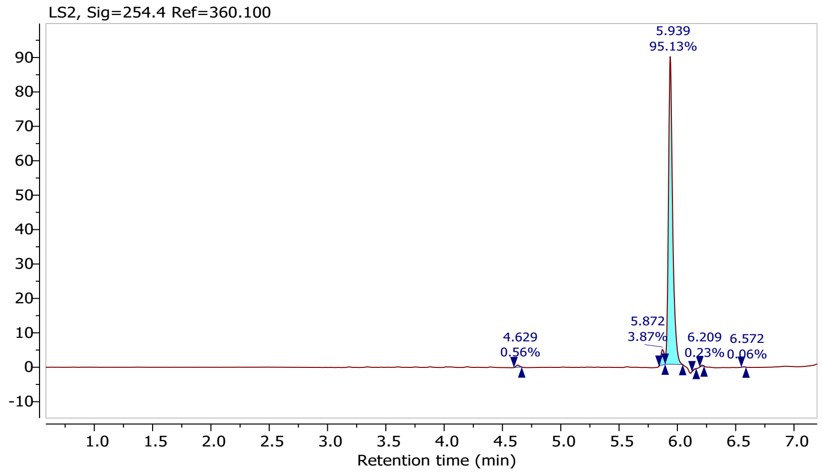

LS2, Sig-254.4 Ref=360.100

Peak #	Retention Time (min)	Type	Height	Area	Total Area %	Start time	End time
1	4.629	BB	0.671	6.608	0.56	4.599	4.665
2	5.872	BB	4.548	45.625	3.87	5.842	5.895
3	5.939	BB	89.458	1121.071	95.13	5.895	6.045
4	6.142	BB	0.251	1.791	0.15	6.125	6.162
5	6.209	BB	0.402	2.713	0.23	6.192	6.229
6	6.572	BB	0.095	0.686	0.06	6.549	6.589

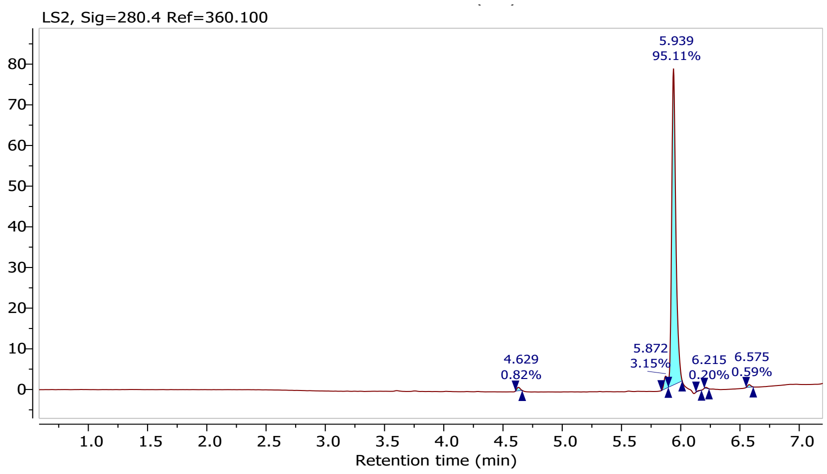

LS2, Sig-280.4 Ref=360.100

Peak #	Retention Time (min)	Type	Height	Area	Total Area %	Start time	End time
1	4.629	BB	0.905	8.186	0.82	4.605	4.662
2	5.872	BV	3.105	31.487	3.15	5.835	5.895
3	5.939	VB	77.801	949.707	95.11	5.895	6.012
4	6.145	BB	0.142	1.212	0.12	6.129	6.175
5	6.215	BB	0.282	1.982	0.20	6.199	6.239
6	6.575	BB	0.647	5.918	0.59	6.552	6.612

LS3

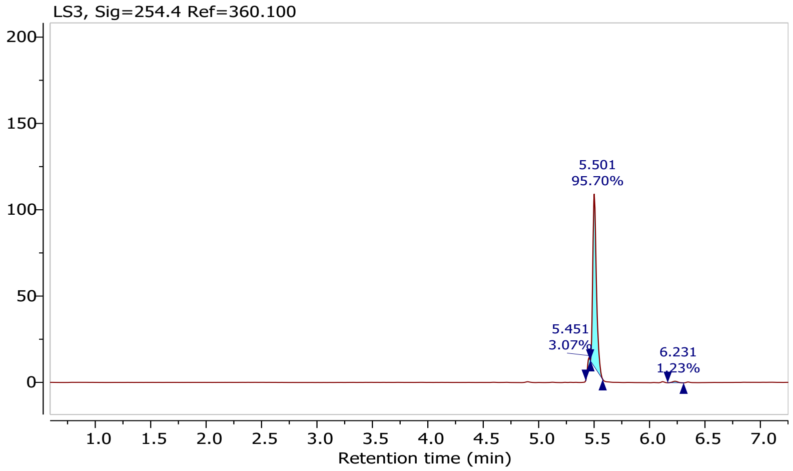

LS3, Sig-254.4 Ref=360.100

Peak #	Retention Time (min)	Type	Height	Area	Total Area %	Start time	End time
1	5.451	BB	5.880	36.770	3.07	5.424	5.467
2	5.501	BB	99.949	1147.575	95.70	5.464	5.577
3	6.231	BB	0.898	14.795	1.23	6.164	6.307

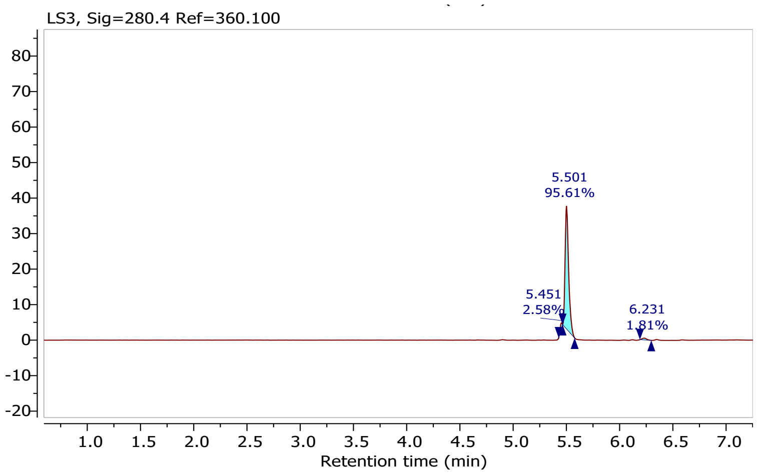

LS3, Sig-284.4 Ref=360.100

Peak #	Retention Time (min)	Type	Height	Area	Total Area %	Start time	End time
1	5.451	BB	1.815	10.742	2.58	5.427	5.464
2	5.501	BB	34.632	398.205	95.61	5.464	5.577
3	6.231	BB	0.514	7.524	1.81	6.191	6.297

LS4

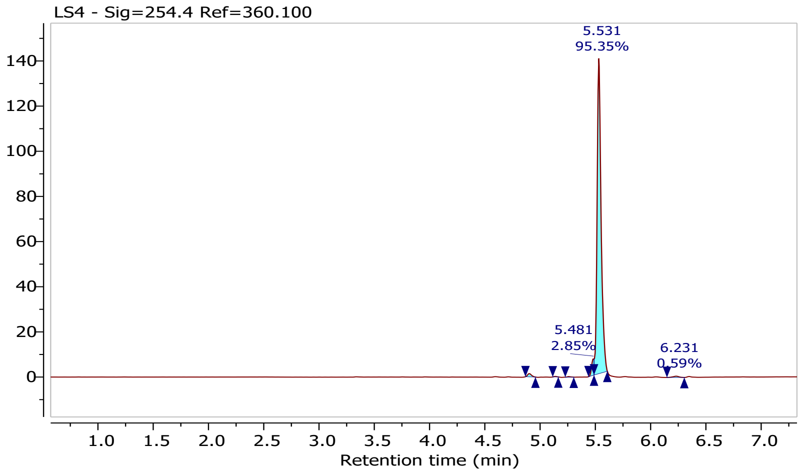

LS4, Sig-254.4 Ref=360.100

Peak #	Retention Time (min)	Type	Height	Area	Total Area %	Start time	End time
1	4.901	BB	1.484	17.685	0.96	4.868	4.958
2	5.135	BB	0.230	2.012	0.11	5.115	5.165
3	5.258	BB	0.256	2.547	0.14	5.228	5.305
4	5.481	BV	7.409	52.503	2.85	5.438	5.488
5	5.531	VB	139.627	1754.620	95.35	5.488	5.608
6	6.231	BB	0.573	10.852	0.59	6.148	6.305

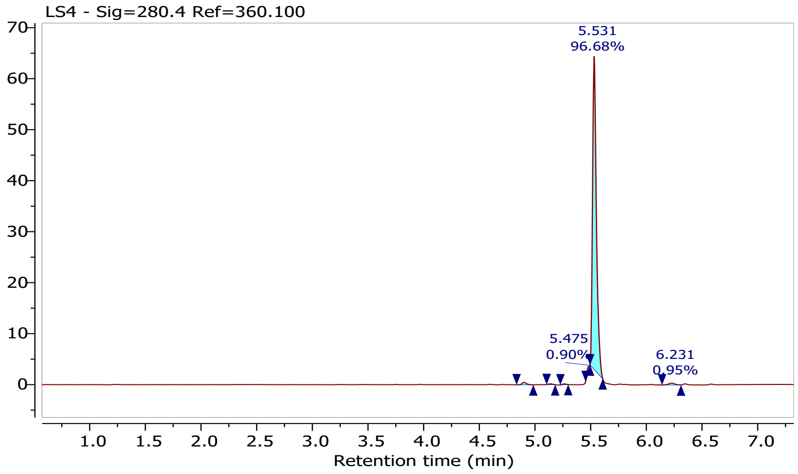

LS4, Sig-280.4 Ref=360.100

Peak #	Retention Time (min)	Type	Height	Area	Total Area %	Start time	End time
1	4.901	BB	0.501	7.044	0.93	4.835	4.985
2	5.138	BB	0.180	2.282	0.30	5.105	5.181
3	5.258	BB	0.195	1.860	0.24	5.228	5.298
4	5.475	BB	1.148	6.827	0.90	5.455	5.495
5	5.531	BB	61.403	734.617	96.68	5.495	5.608
6	6.231	BV	0.367	7.232	0.95	6.141	6.311

^{1}H and ^{13}C NMR Spectra

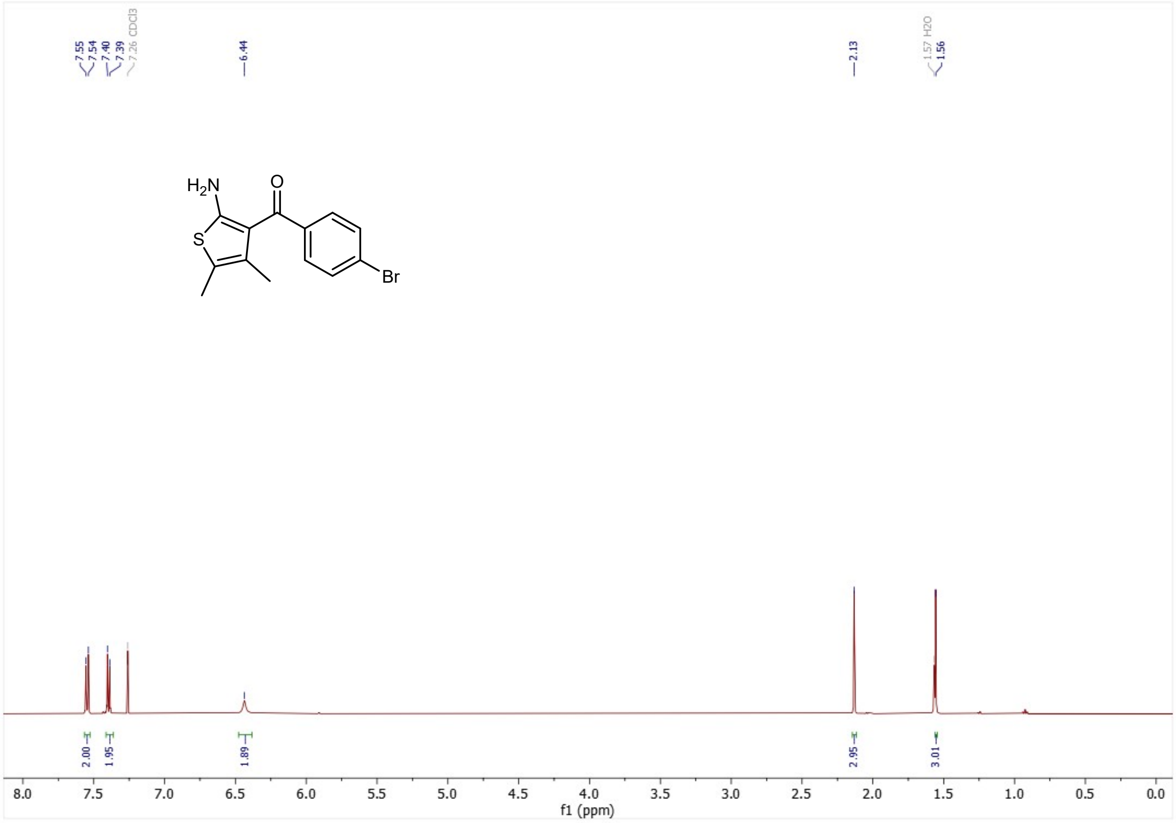

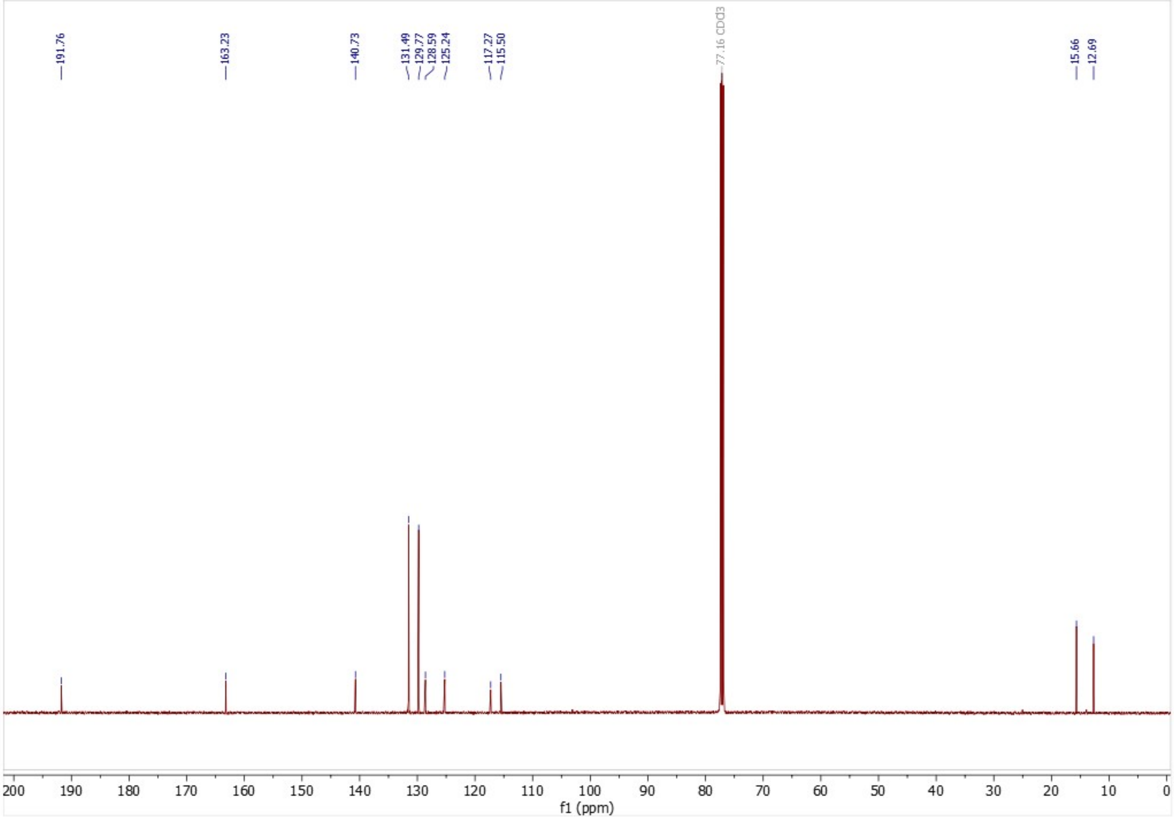

Figure S19. ^{1}H NMR (top) and ^{13}C NMR (bottom) spectra for **3a**.

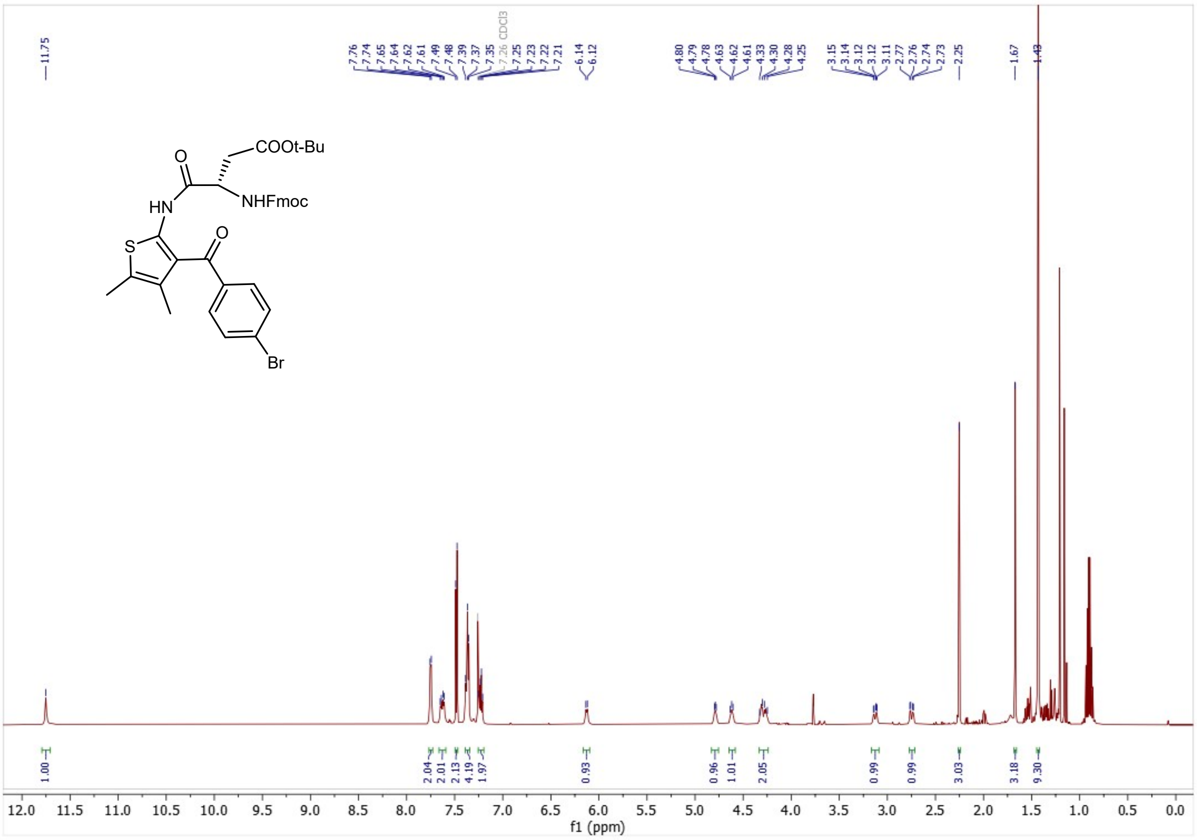

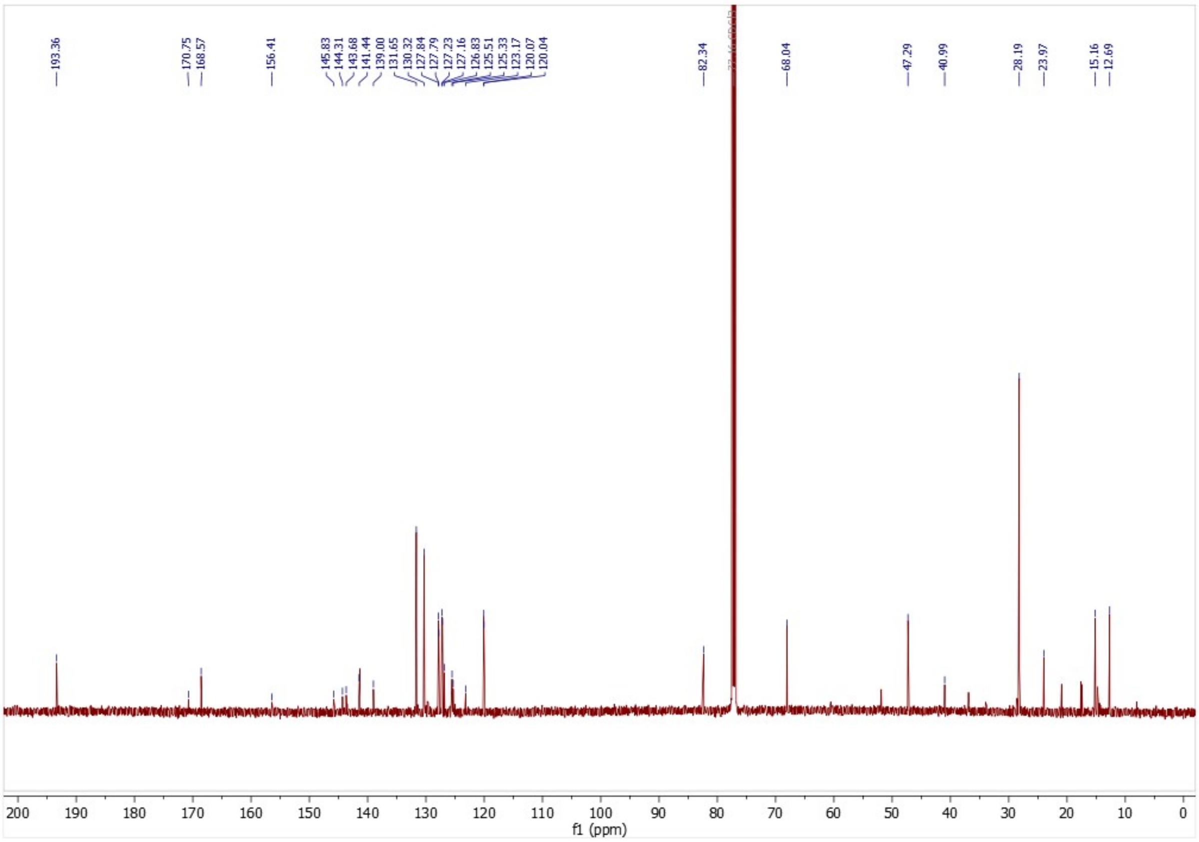

Figure S20. ^{1}H NMR (top) and ^{13}C NMR (bottom) spectra for **4a**.

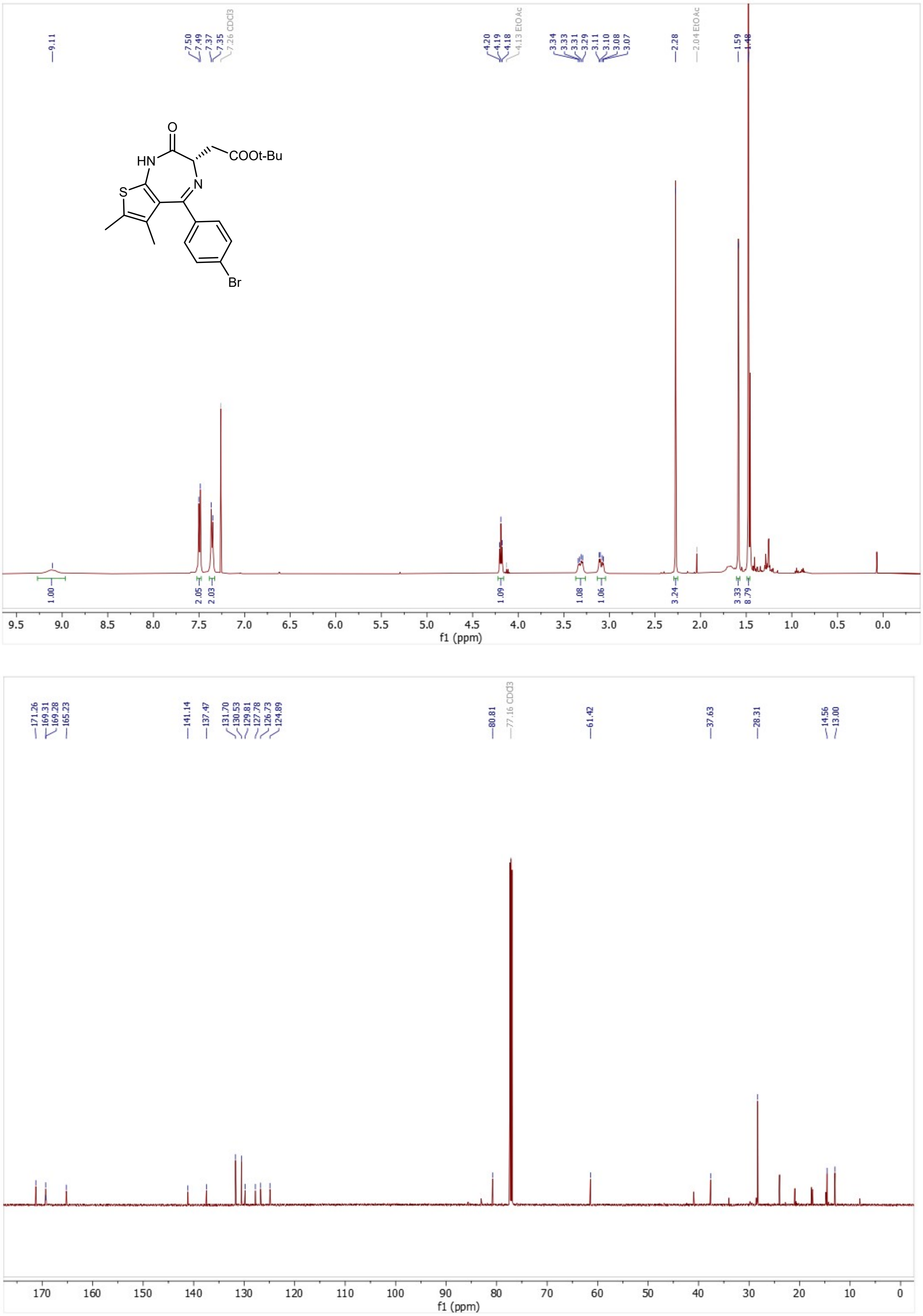

Figure S21. ^{1}H NMR (top) and ^{13}C NMR (bottom) spectra for **5a**.

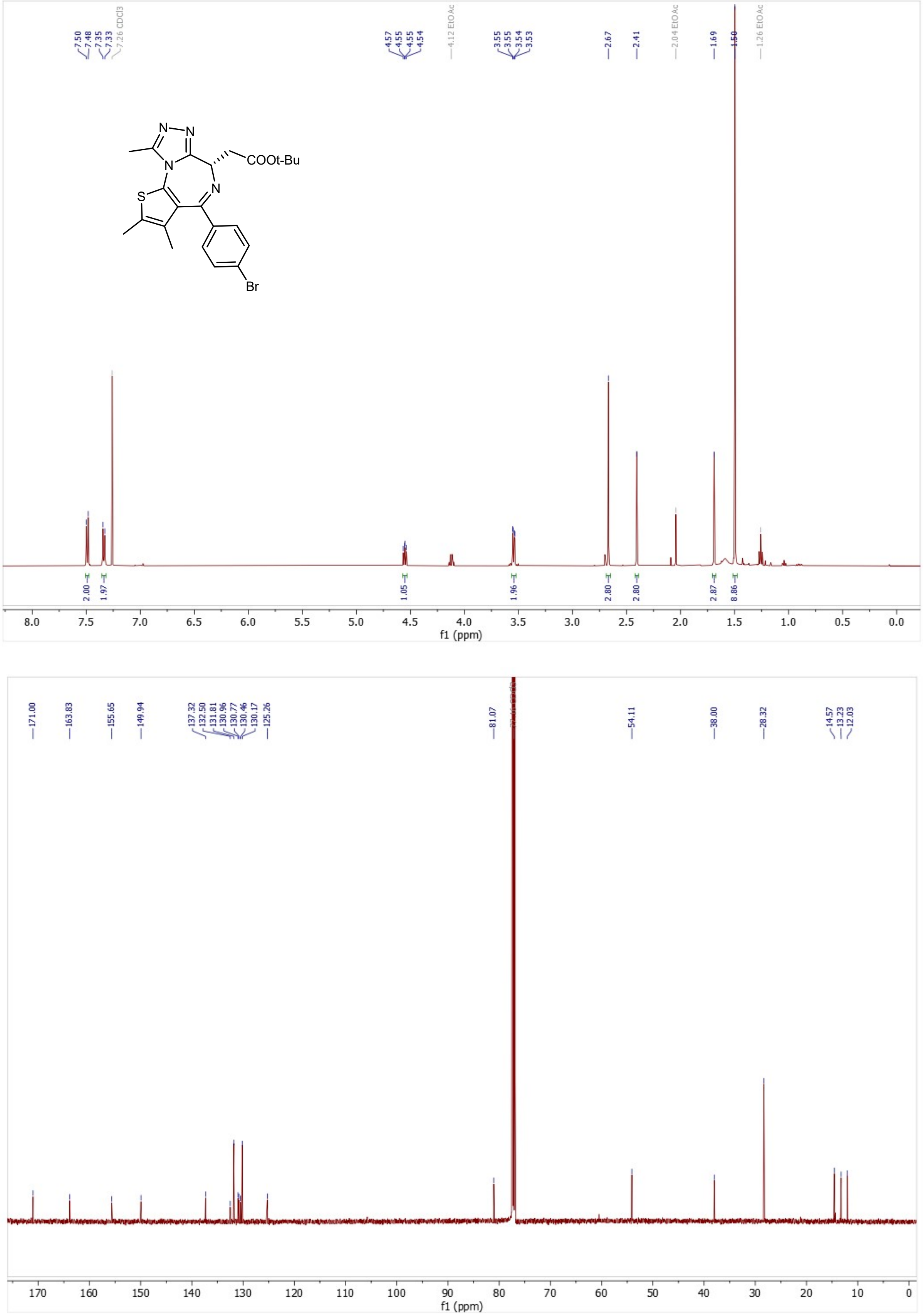

Figure S22. ^{1}H NMR (top) and ^{13}C NMR (bottom) spectra for **6**.

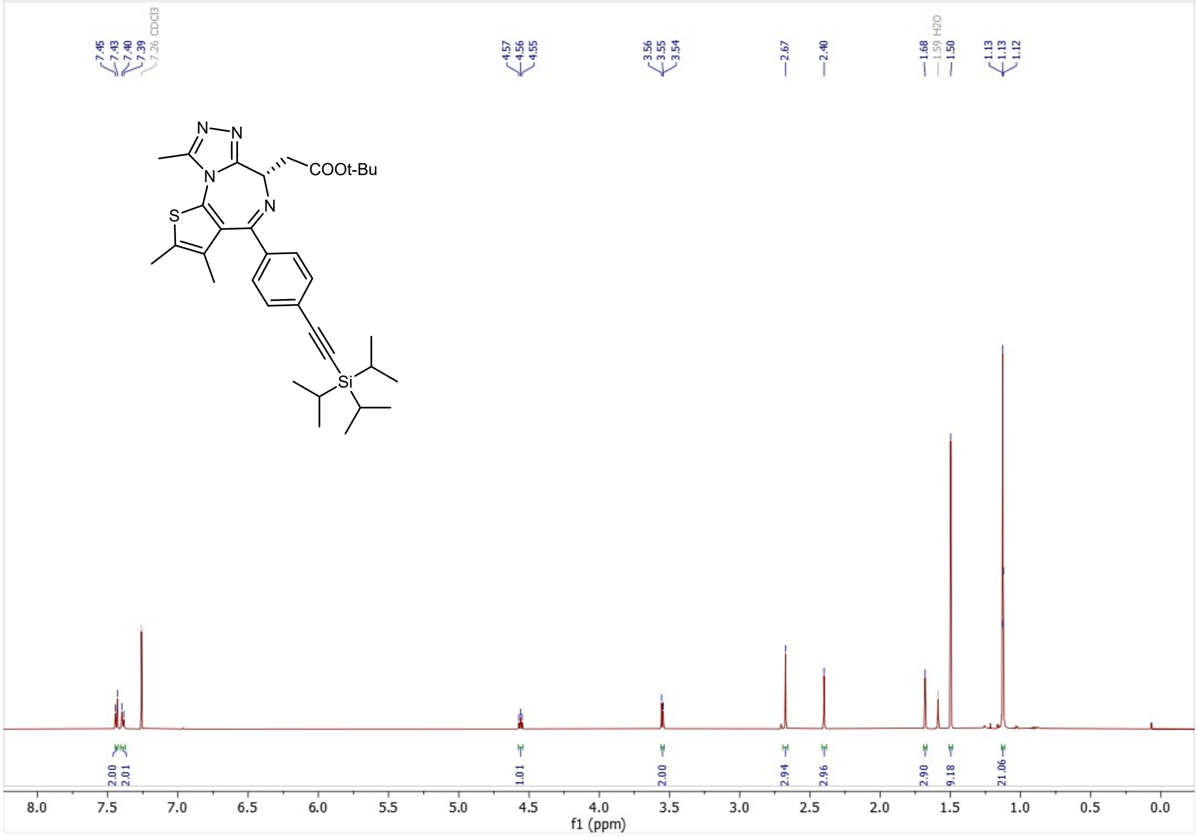

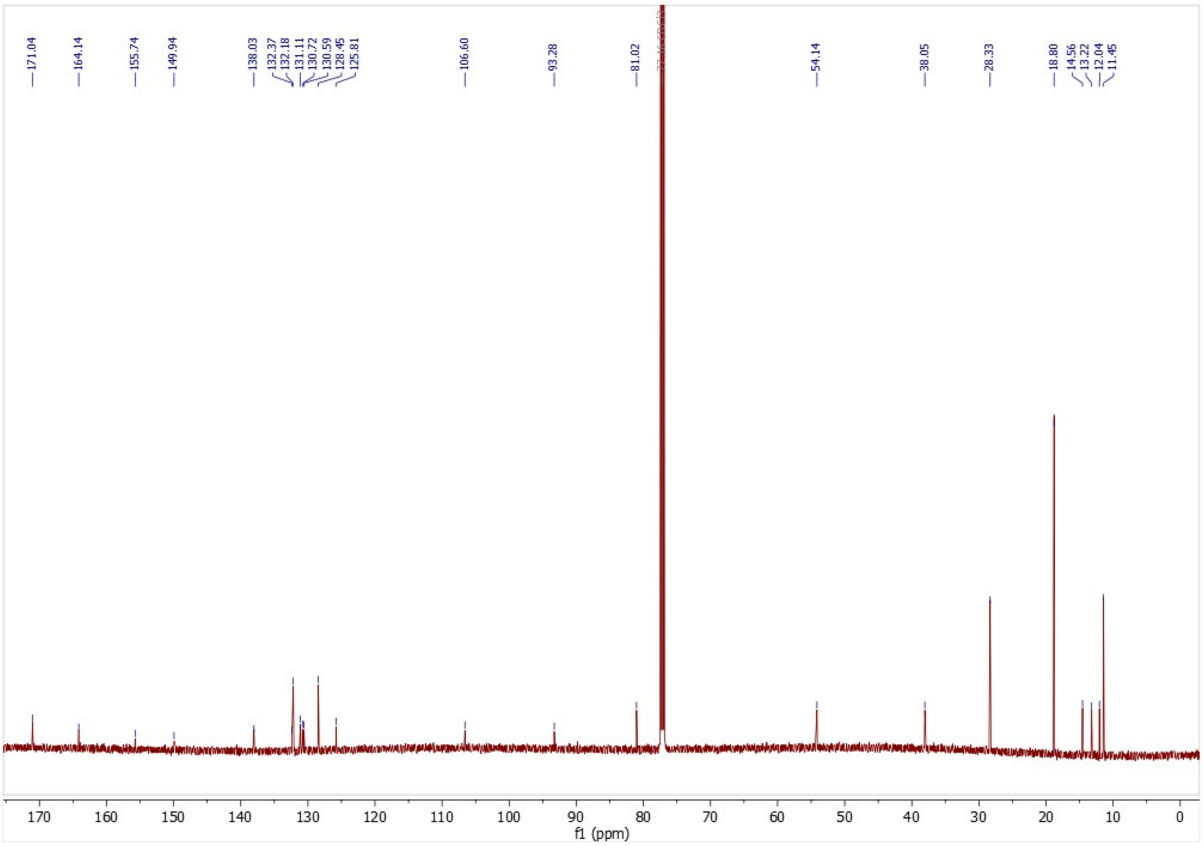

Figure S23. ¹H NMR (top) and ¹³C NMR (bottom) spectra for **7**.

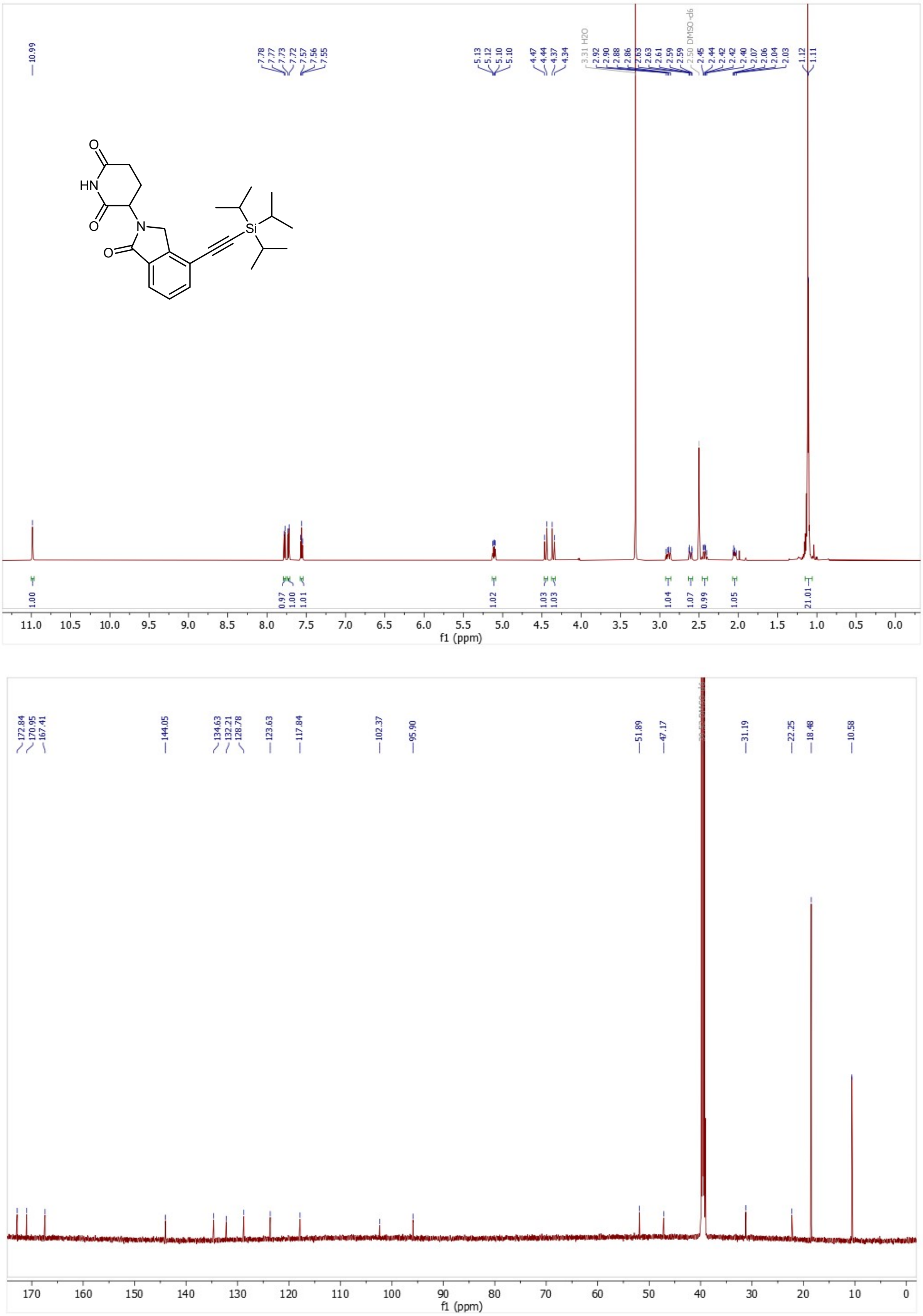

Figure S24. ^{1}H NMR (top) and ^{13}C NMR (bottom) spectra for **10**.

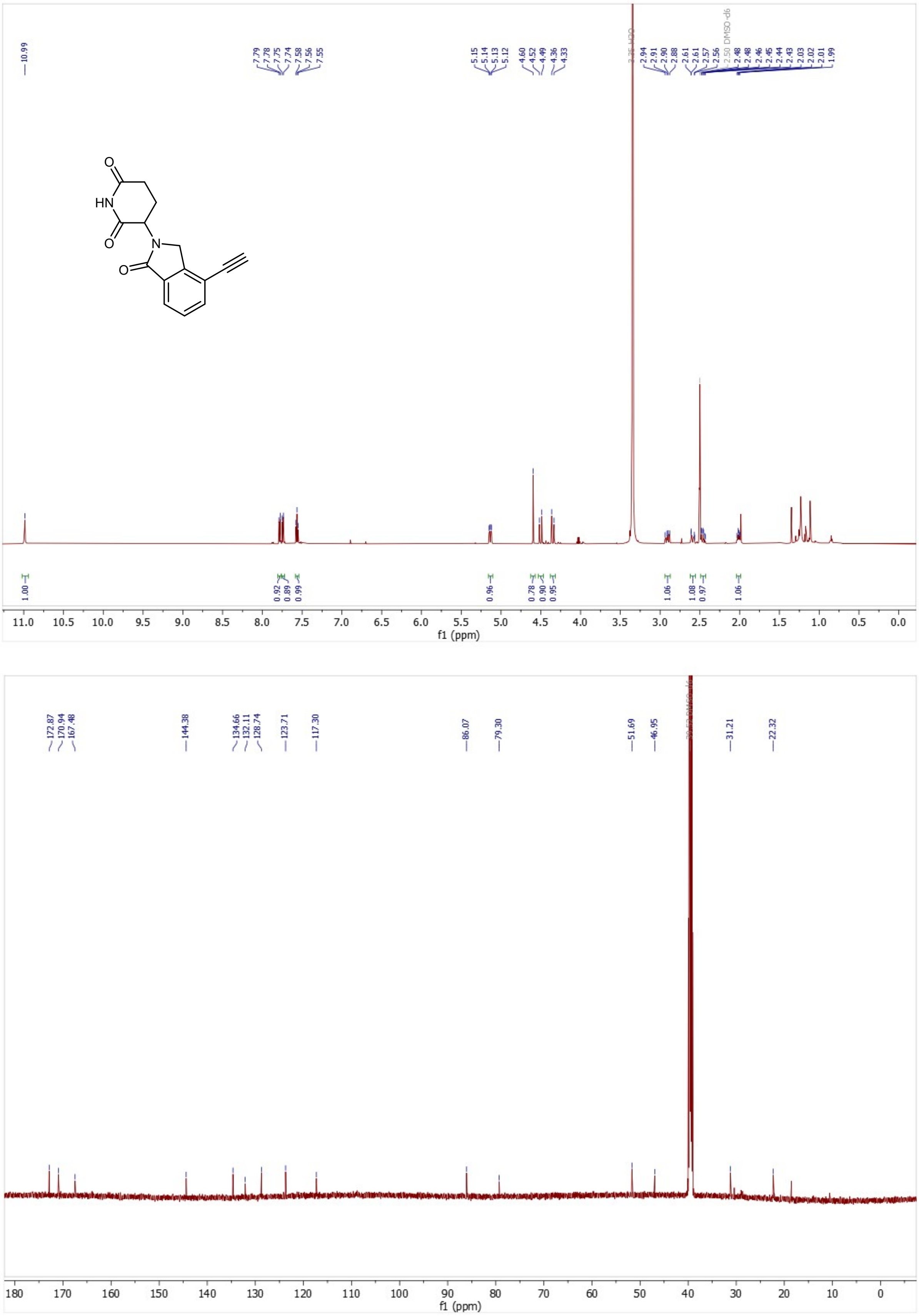

Figure S25. ^{1}H NMR (top) and ^{13}C NMR (bottom) spectra for **11**.

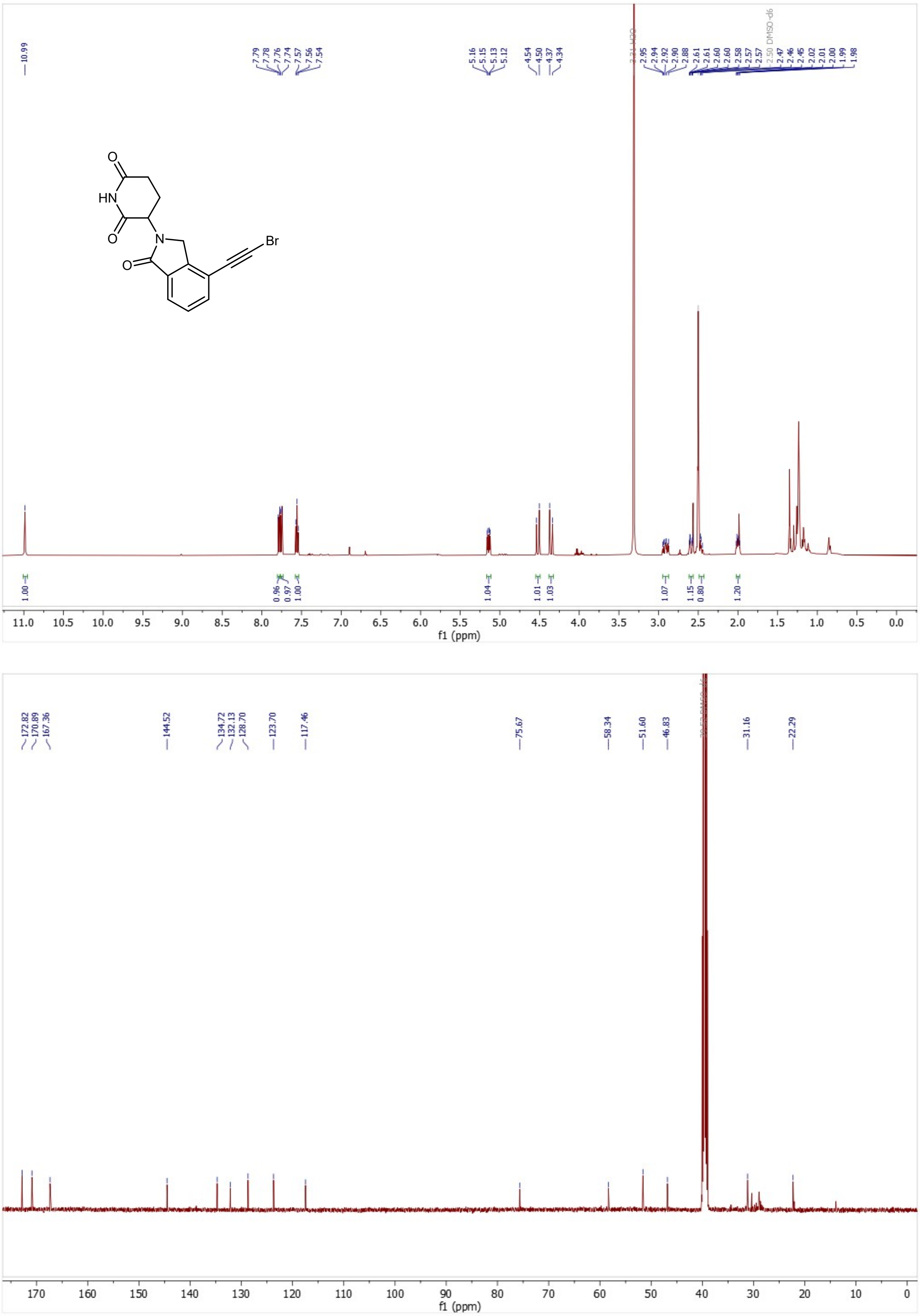

Figure S26. ^{1}H NMR (top) and ^{13}C NMR (bottom) spectra for **12**.

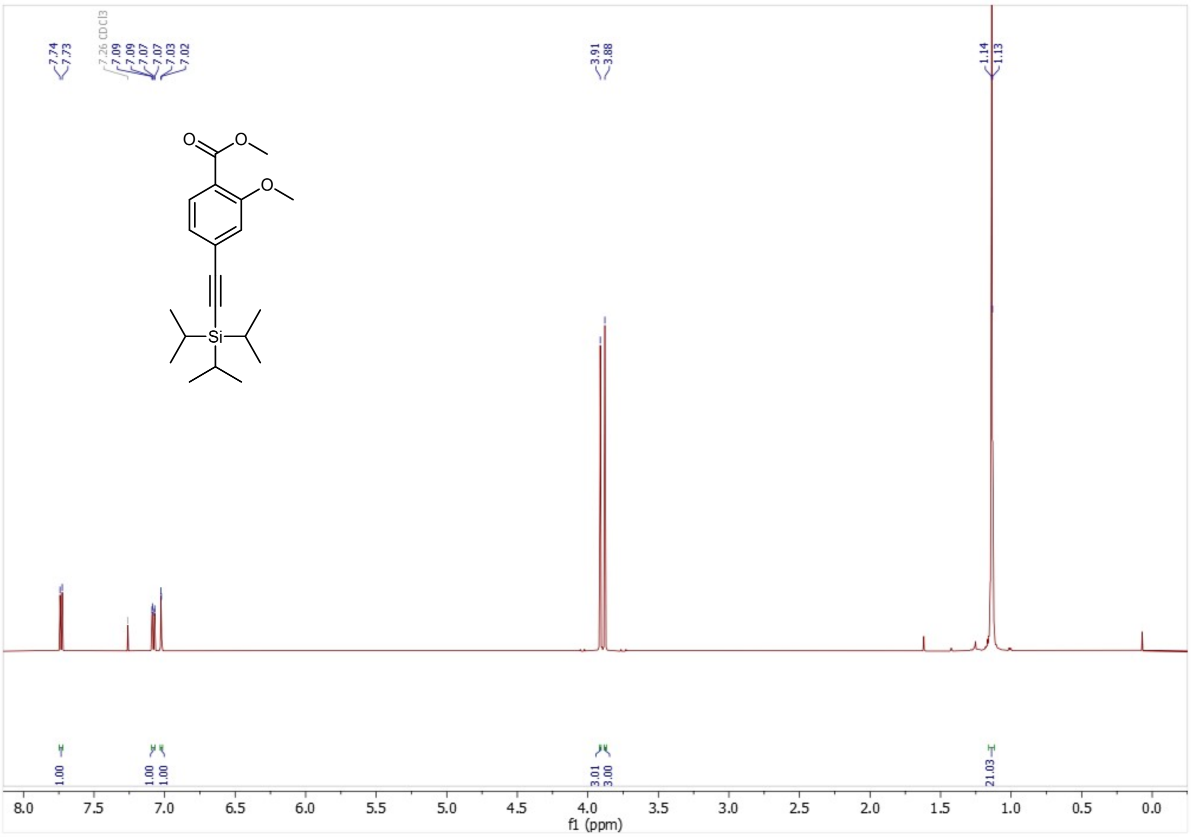

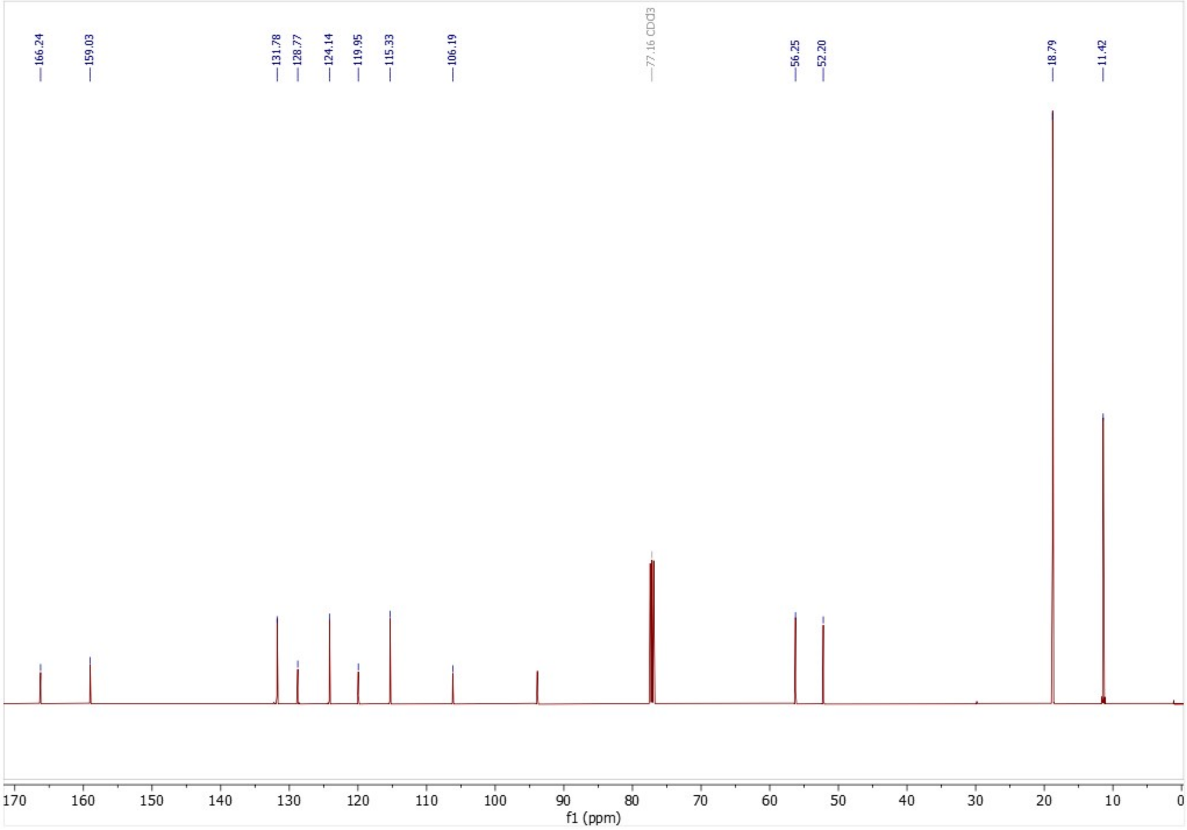

Figure S27. ^1H NMR (top) and ^{13}C NMR (bottom) spectra for **15**.

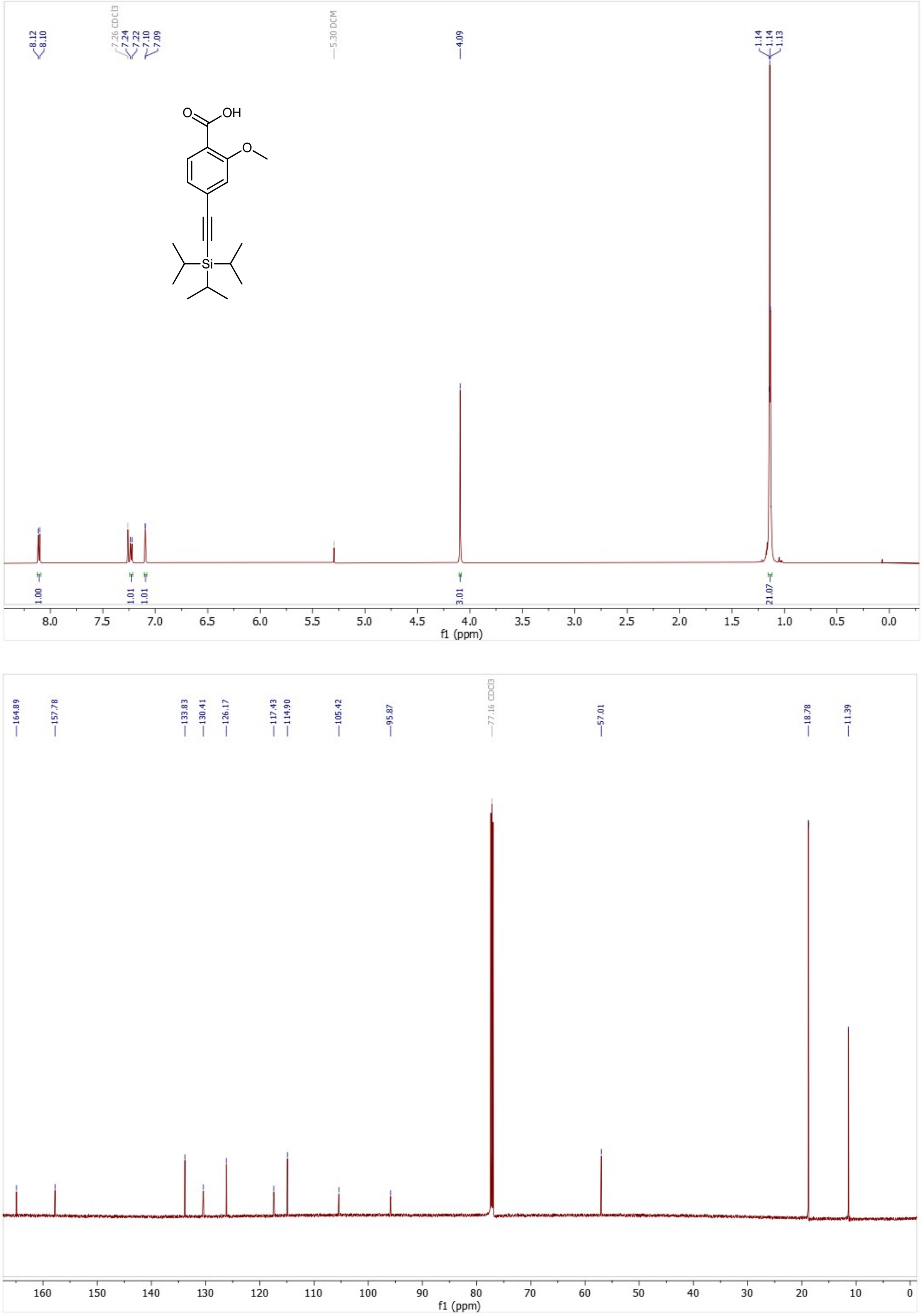

Figure S28. ^{1}H NMR (top) and ^{13}C NMR (bottom) spectra for **16**.

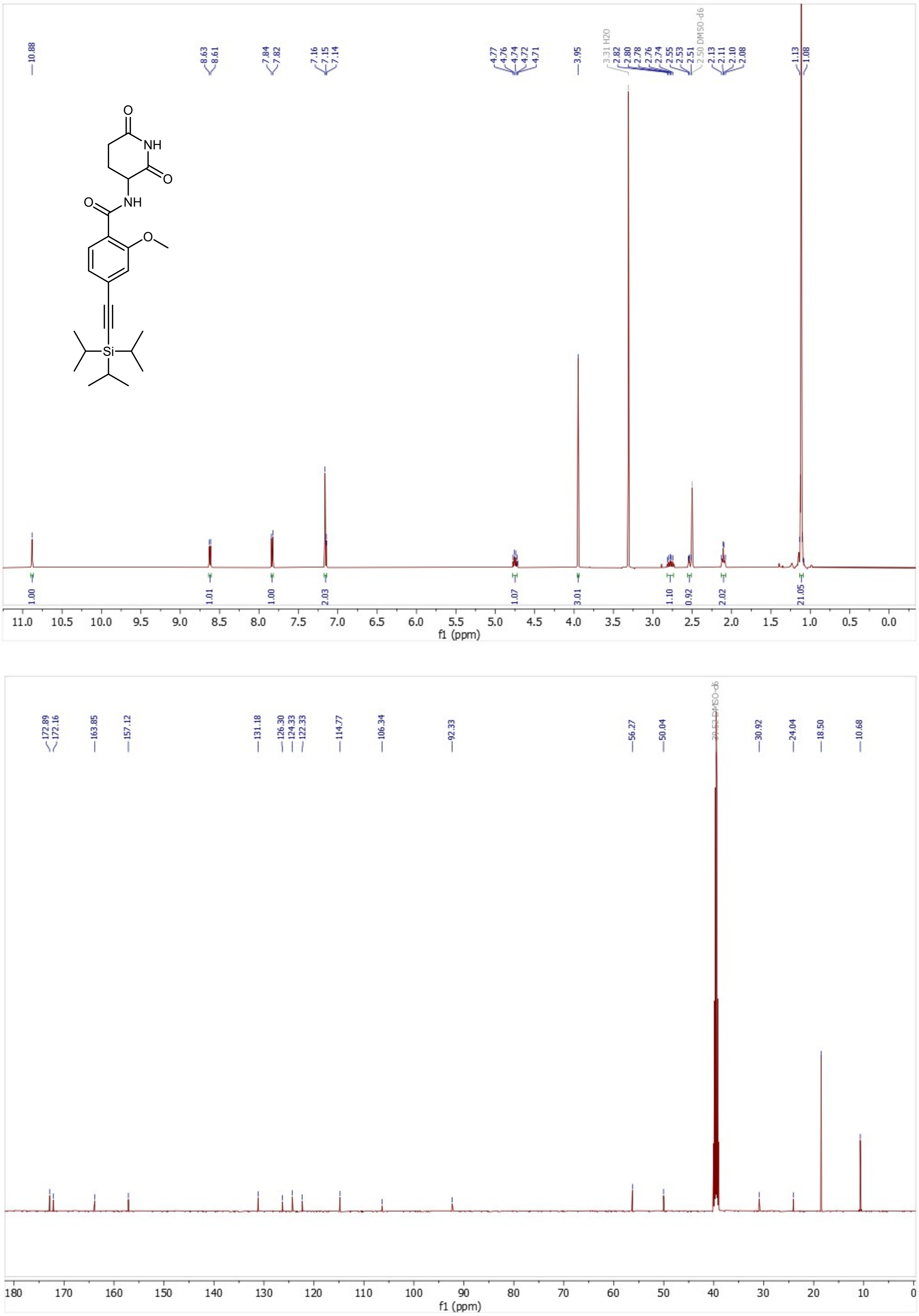

Figure S29. ^{1}H NMR (top) and ^{13}C NMR (bottom) spectra for **17**.

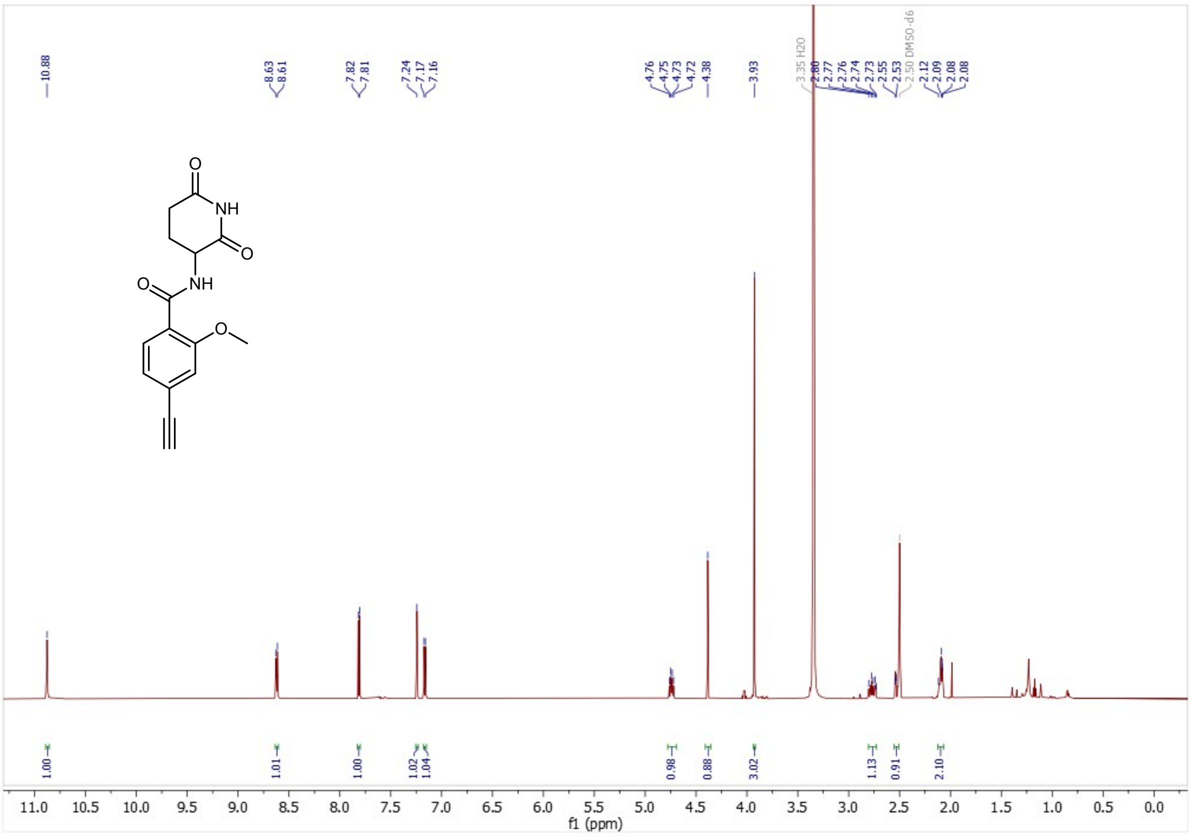

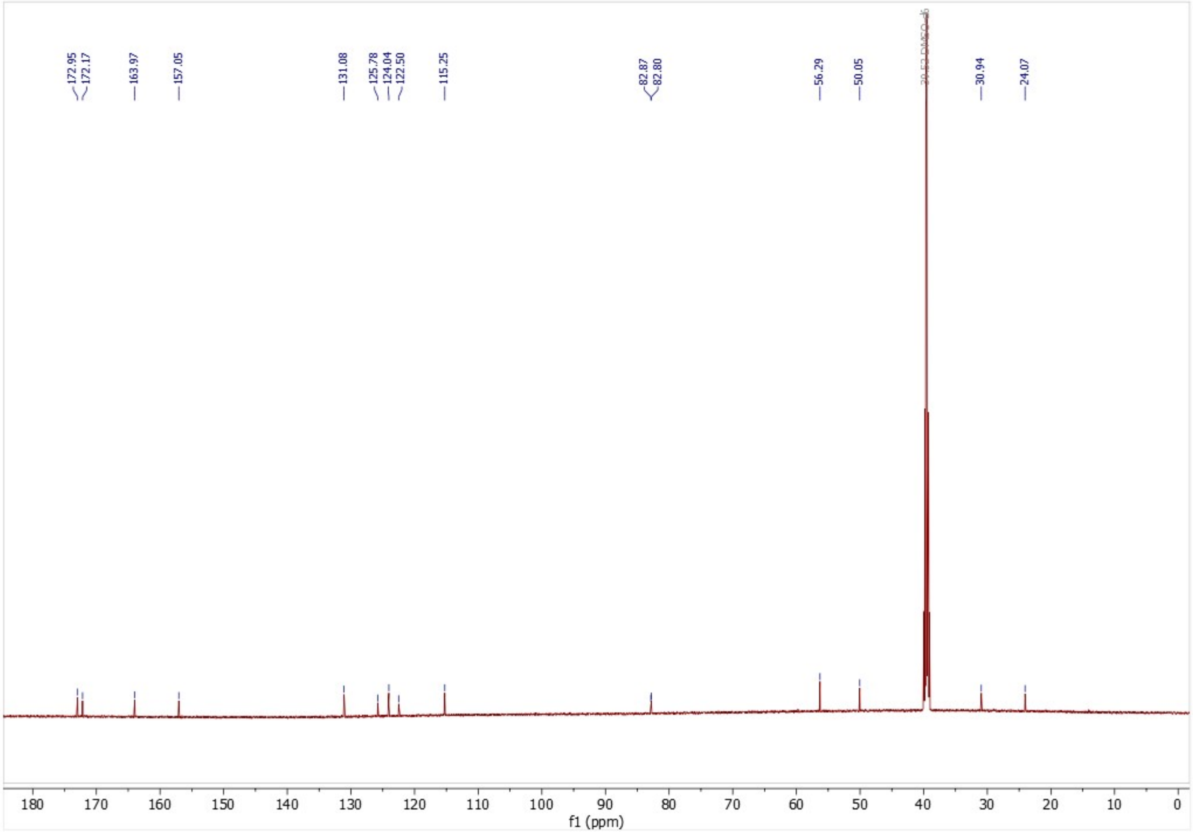

Figure S30. ^{1}H NMR (top) and ^{13}C NMR (bottom) spectra for **18**.

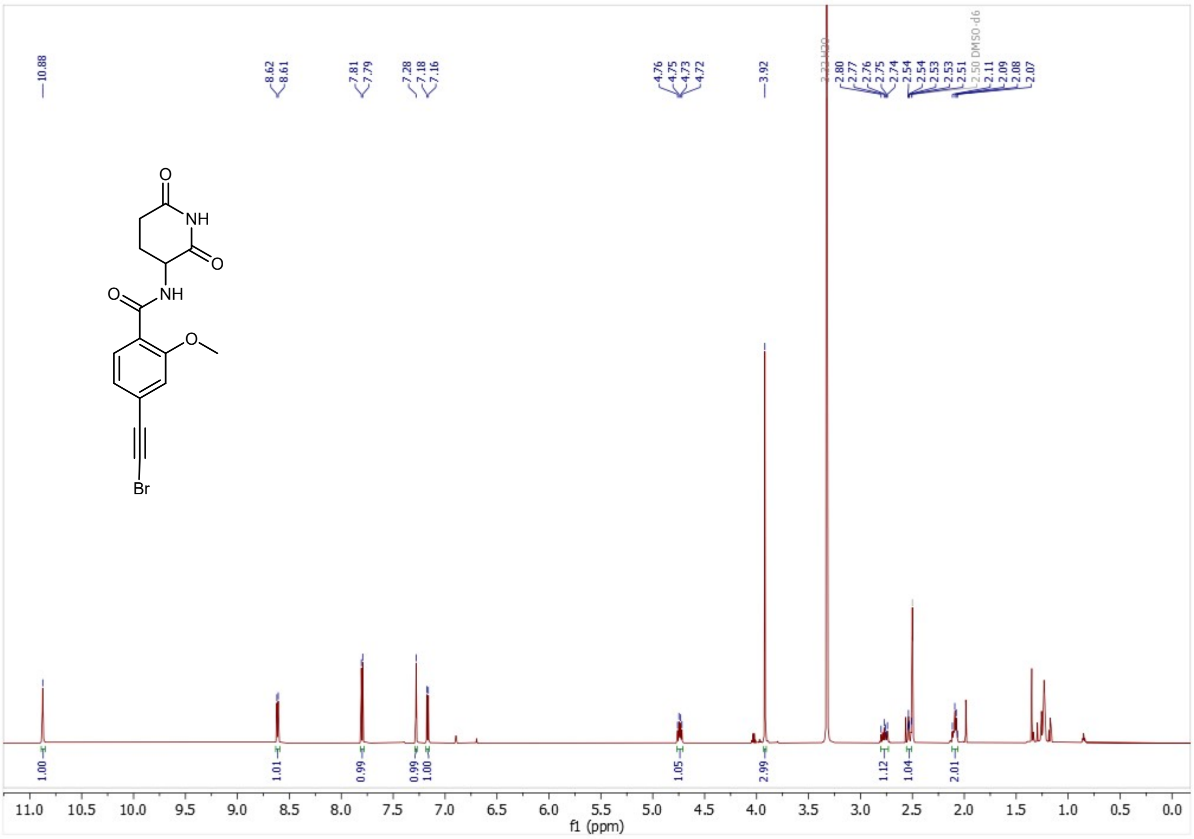

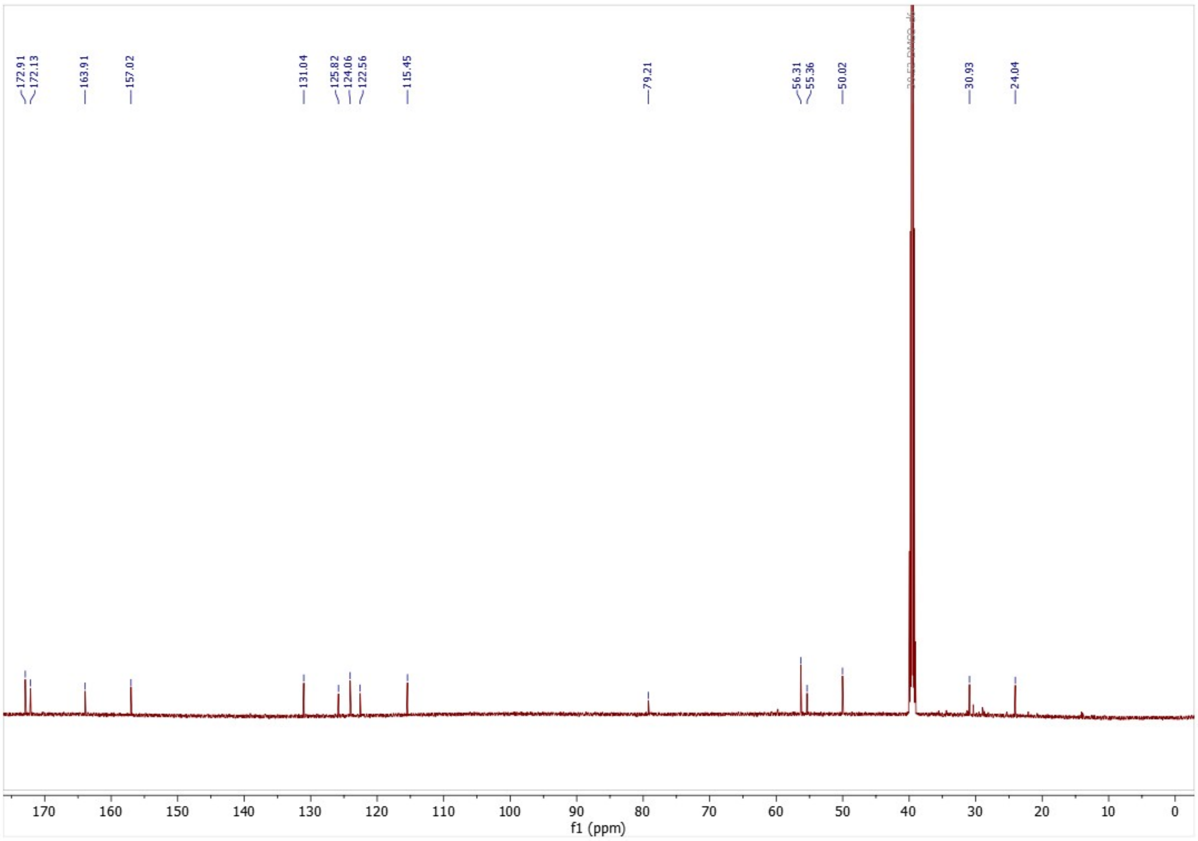

Figure S31. ^{1}H NMR (top) and ^{13}C NMR (bottom) spectra for **19**.

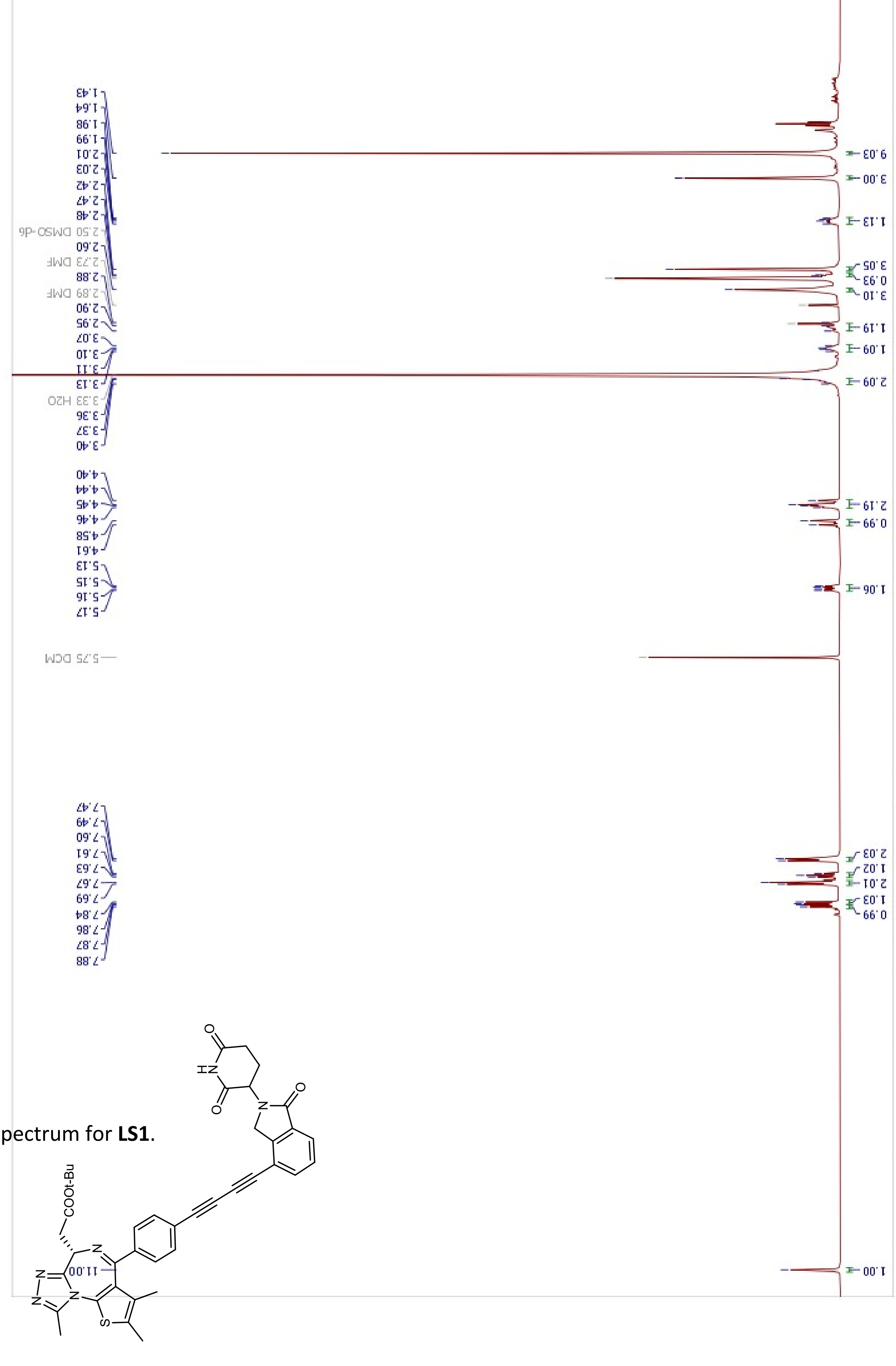

Figure S32. ^{1}H NMR spectrum for **LS1**.

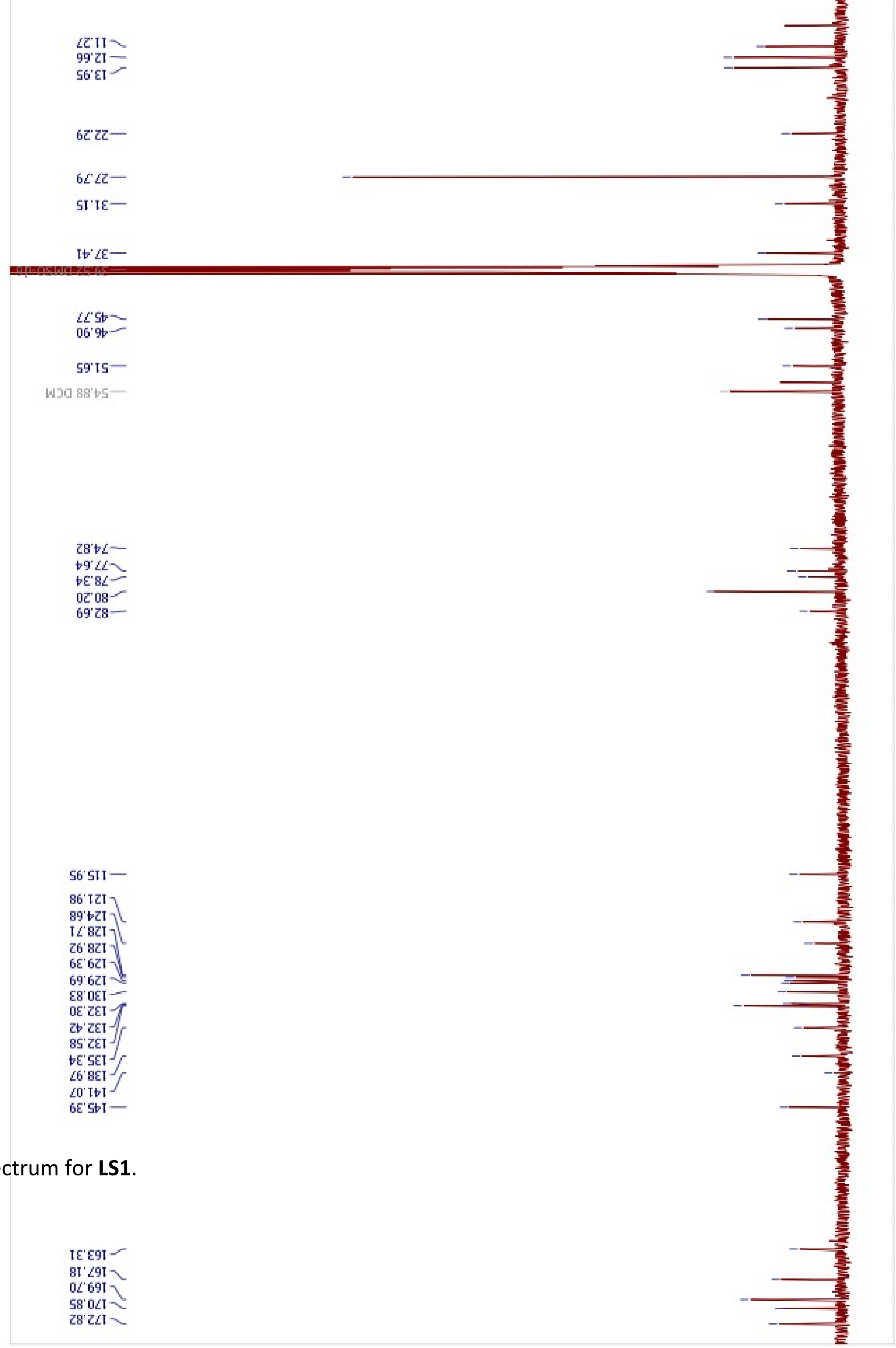

Figure S33. ^{13}C NMR spectrum for **LS1**.

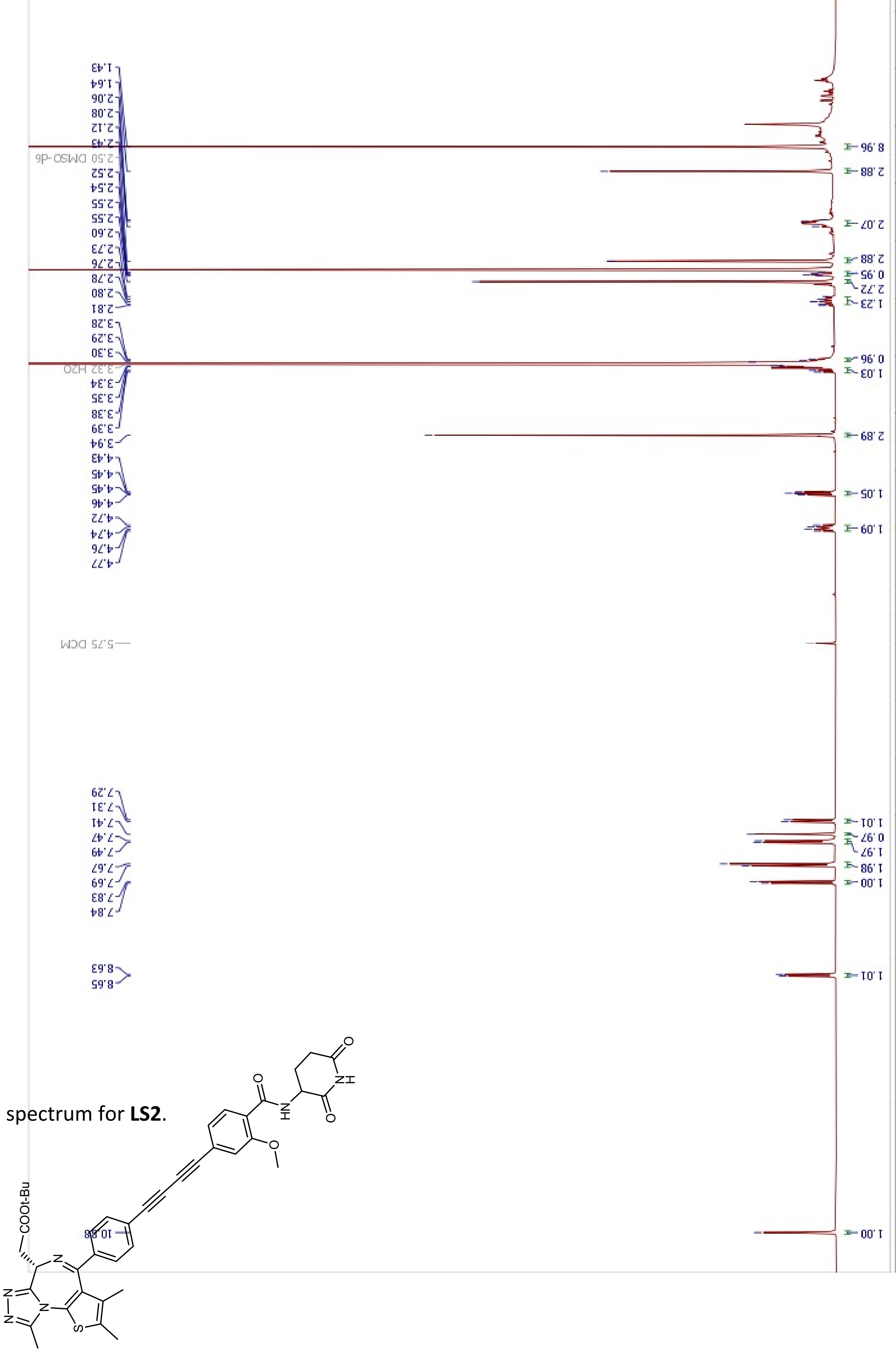

Figure S34. ^{1}H NMR spectrum for **LS2**.

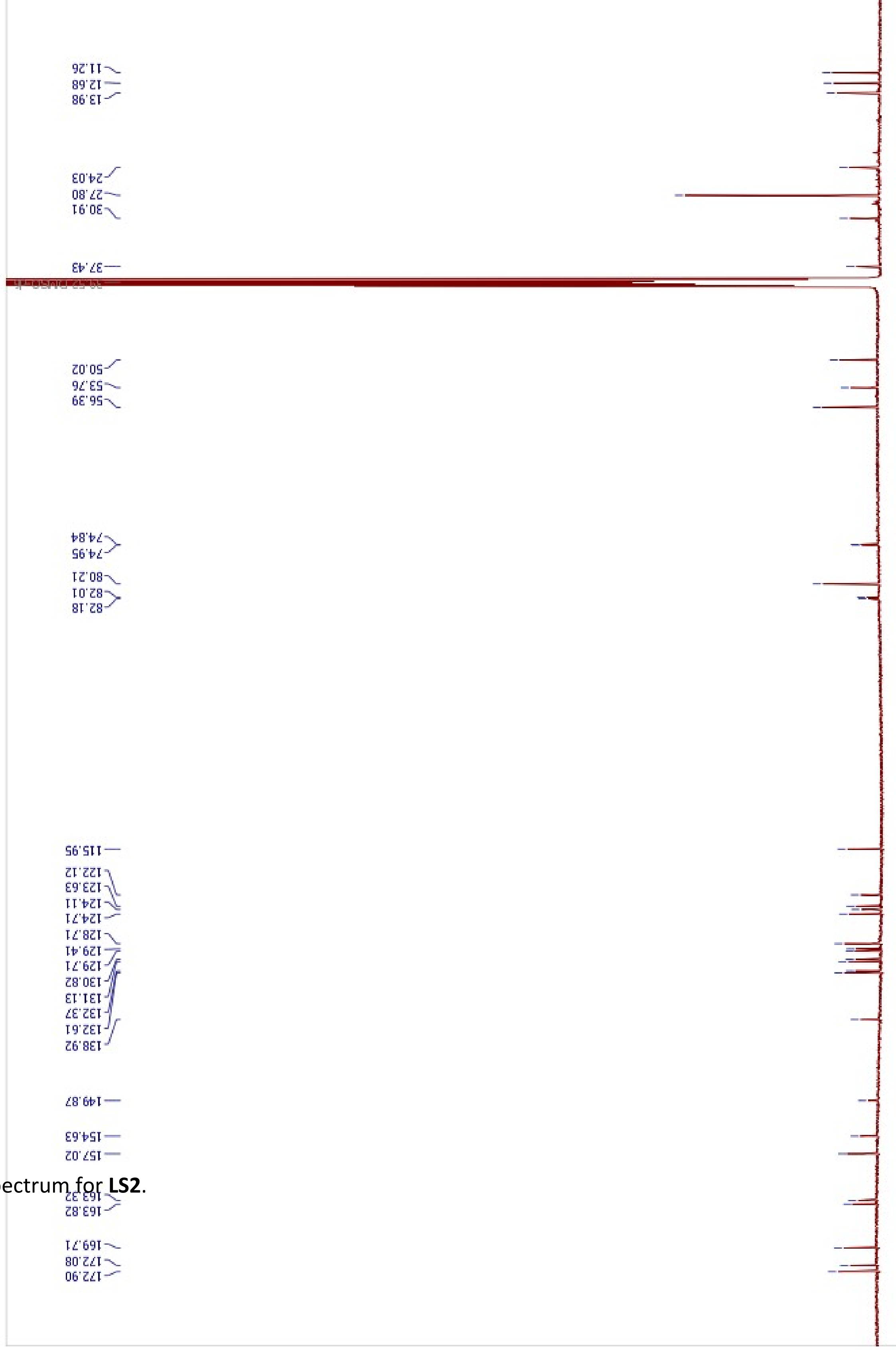

Figure S35. ^{13}C NMR spectrum for **LS2**.

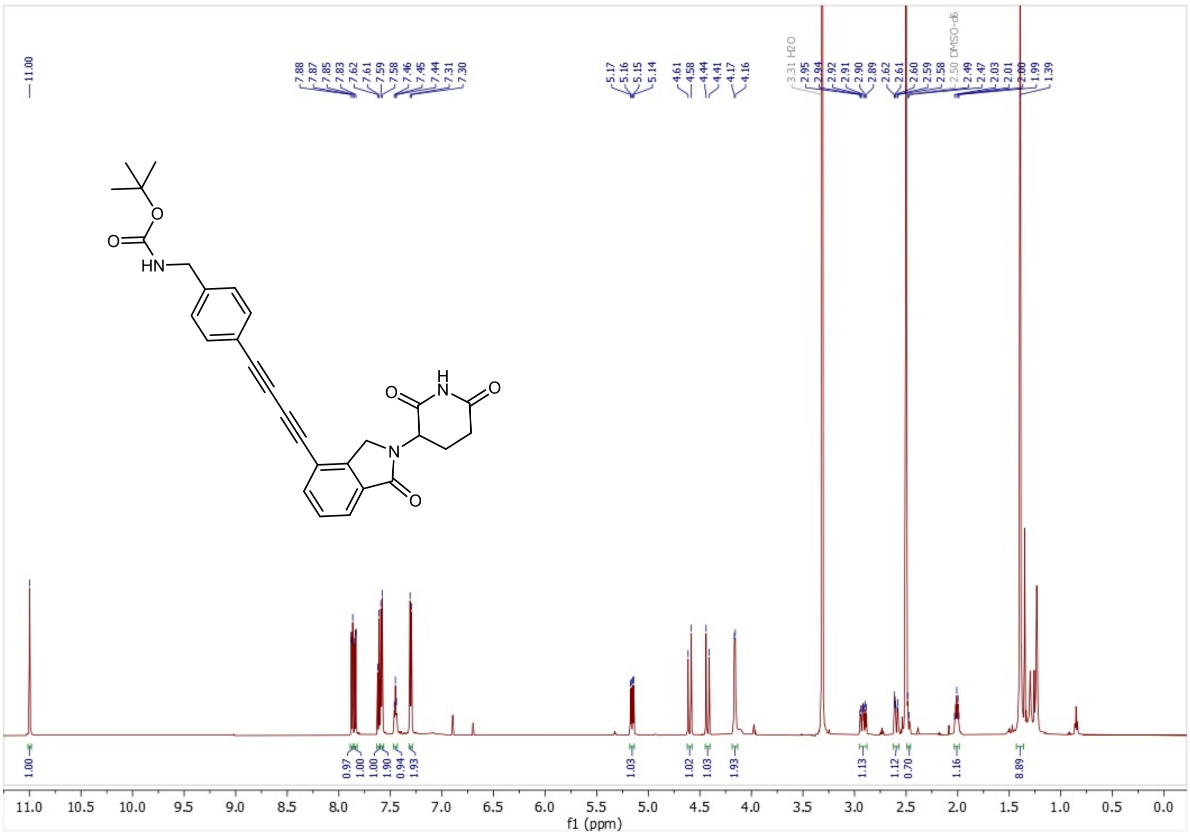

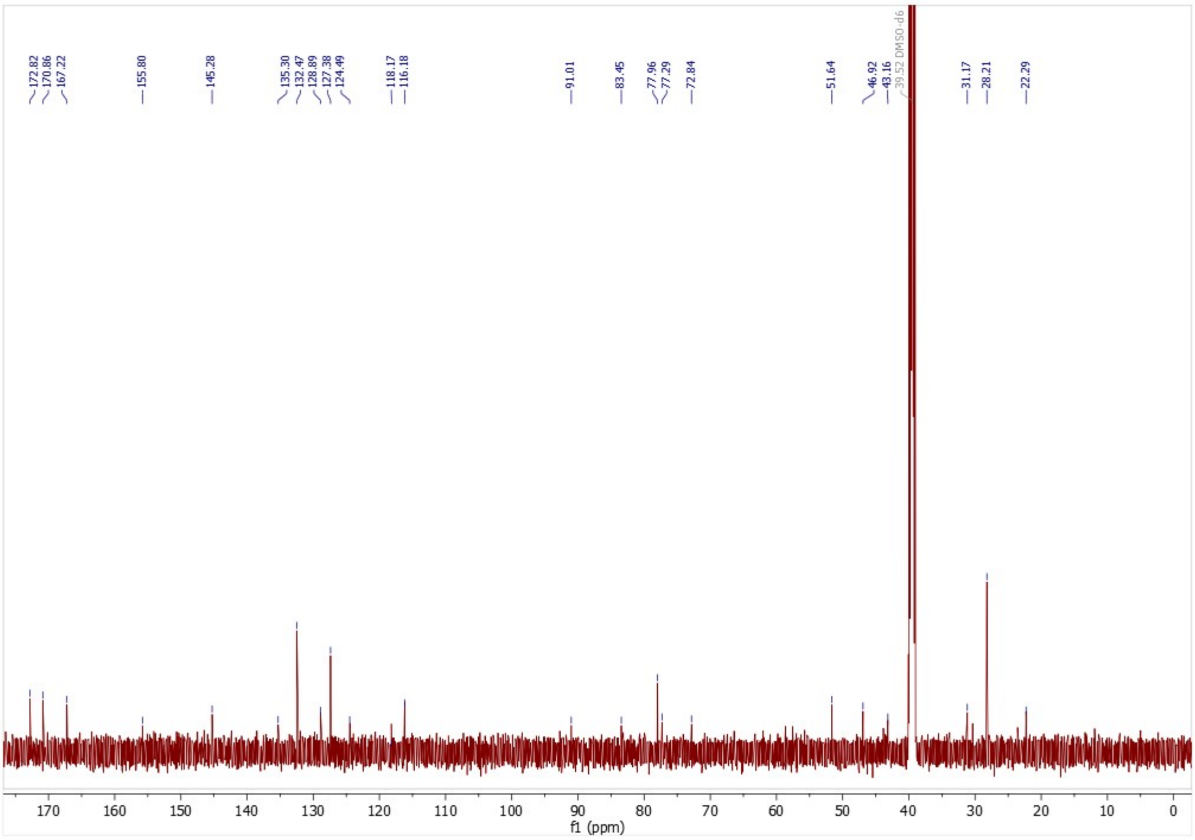

Figure S36. ^{1}H NMR (top) and ^{13}C NMR (bottom) spectra for **22**.

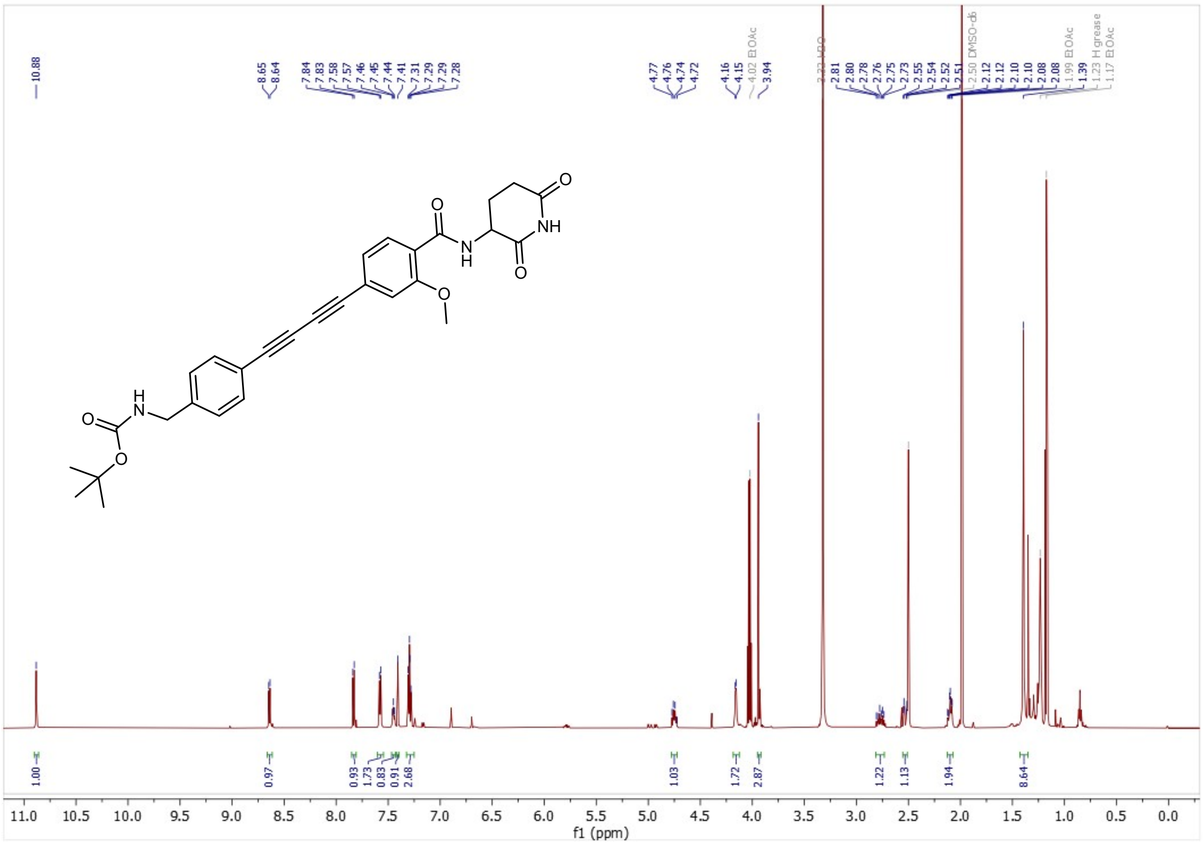

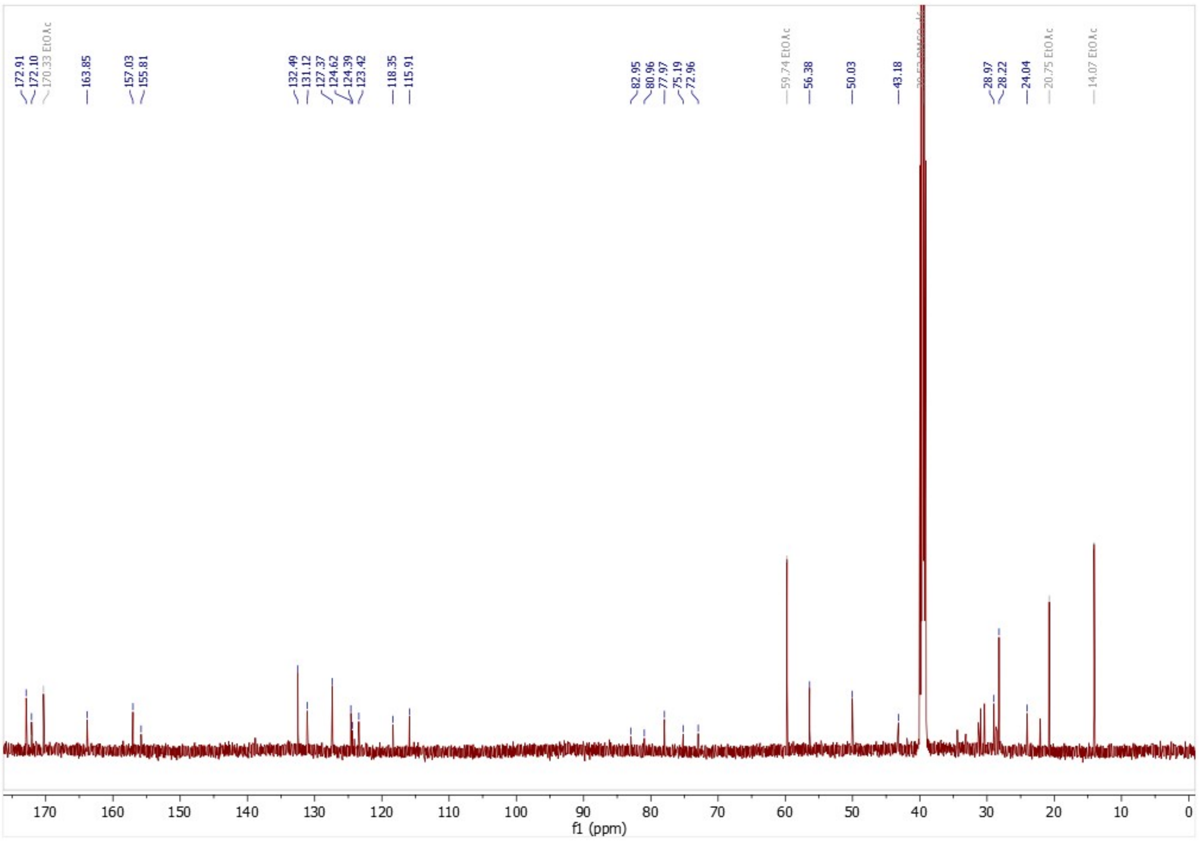

Figure S37. ^{1}H NMR (top) and ^{13}C NMR (bottom) spectra for **24**.

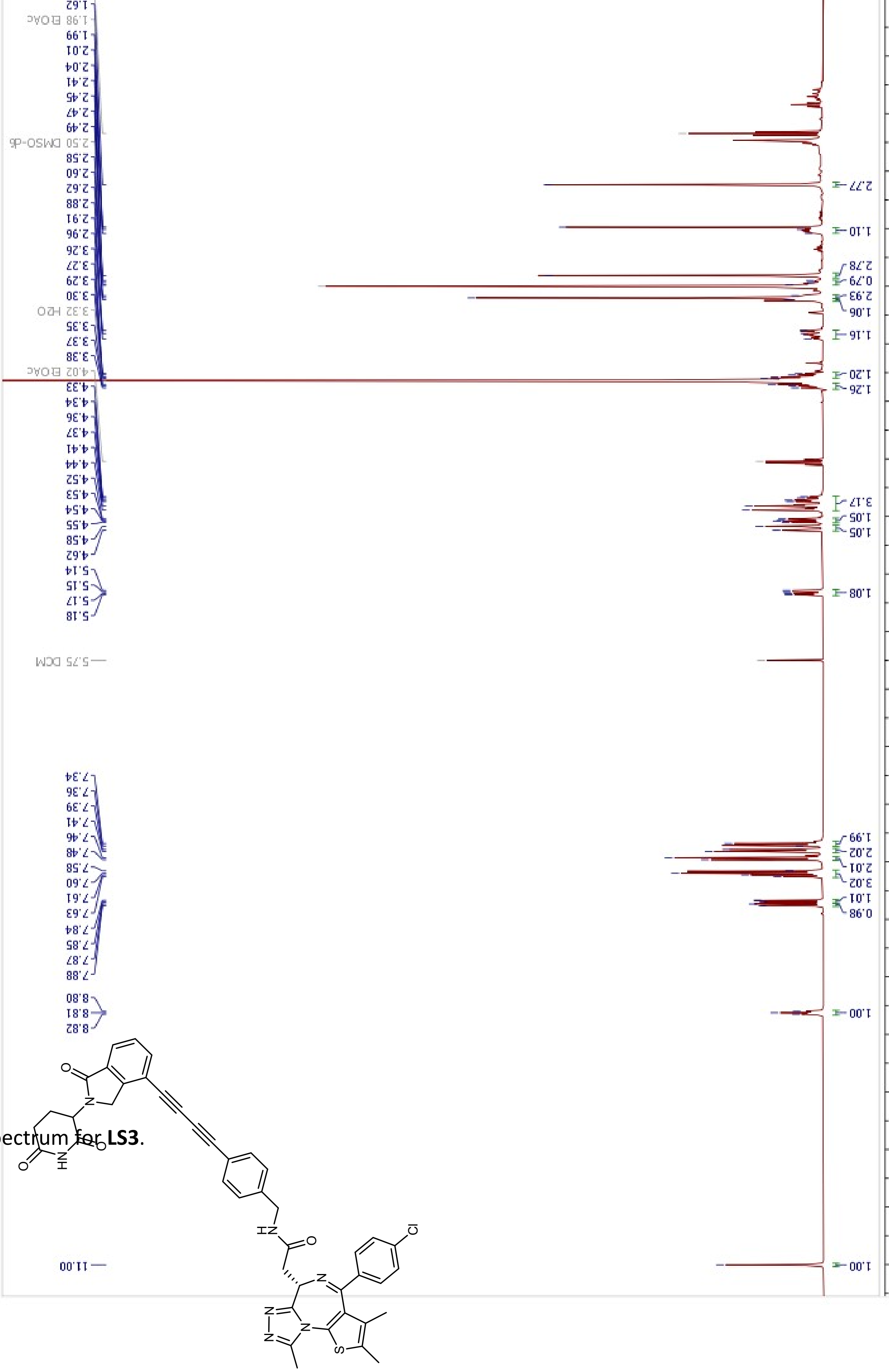

Figure S38. ^{1}H NMR spectrum for **LS3**.

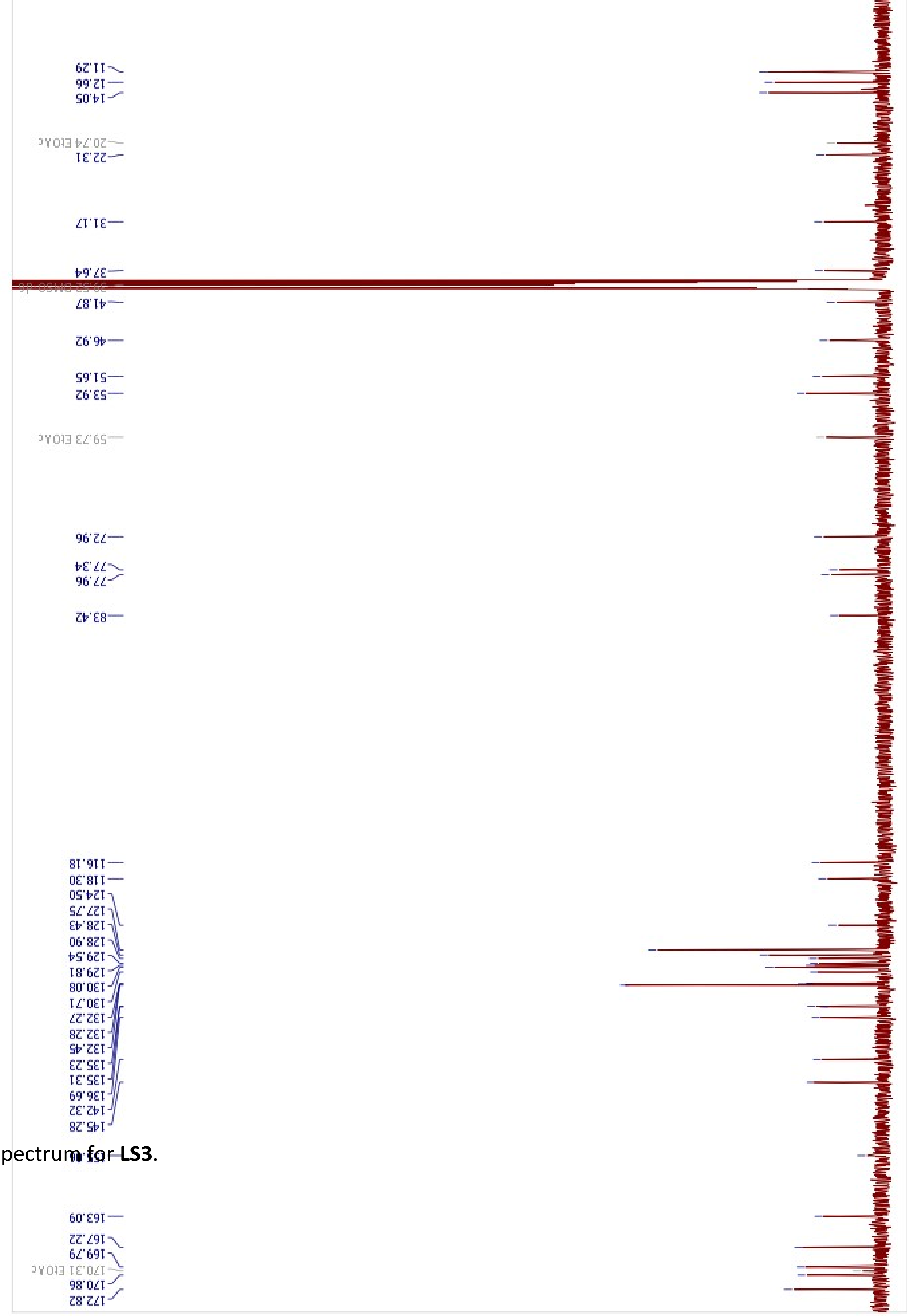

Figure S39. ^{13}C NMR spectrum for **LS3**.

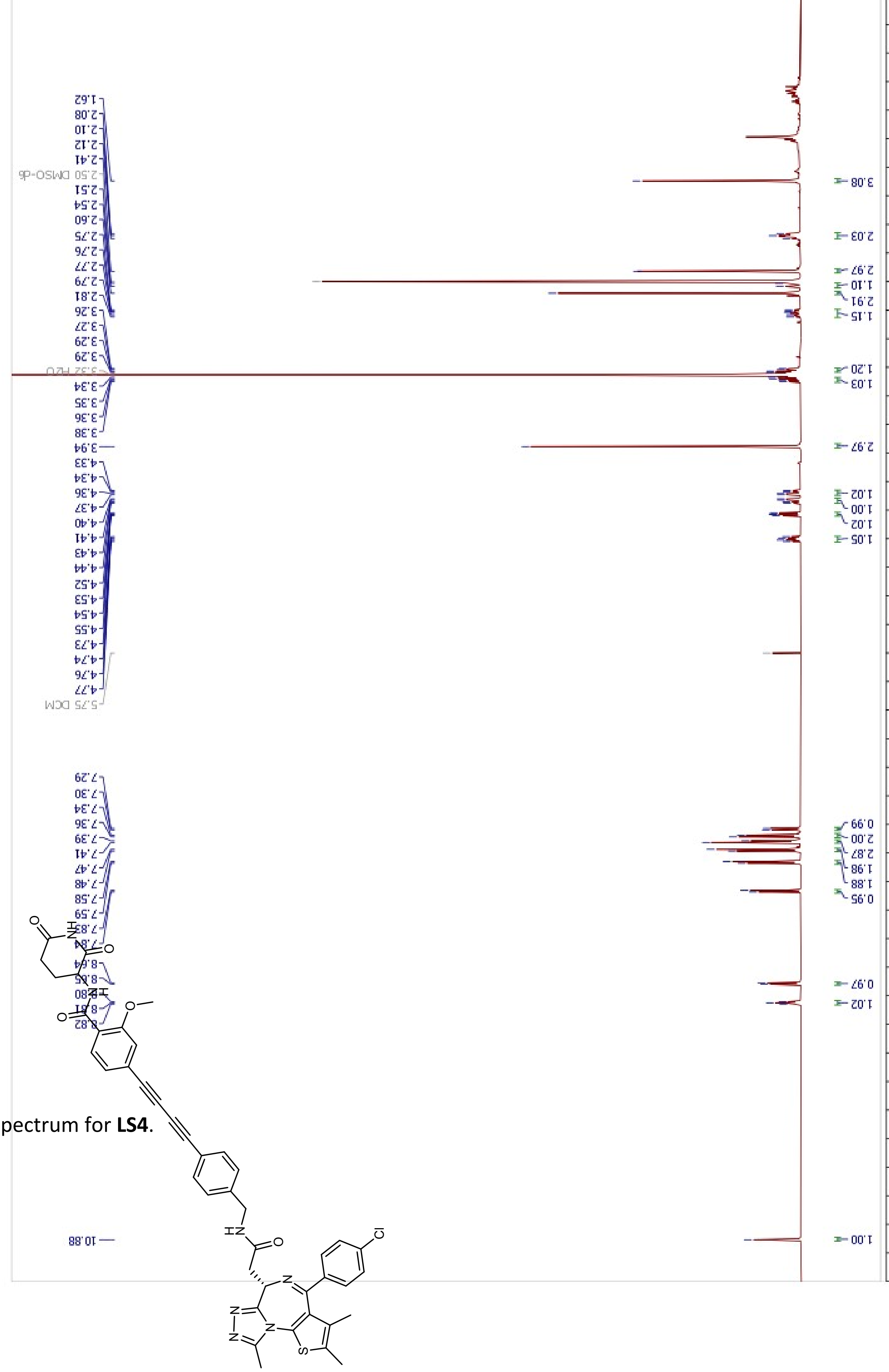

Figure S40. ^{1}H NMR spectrum for **LS4**.

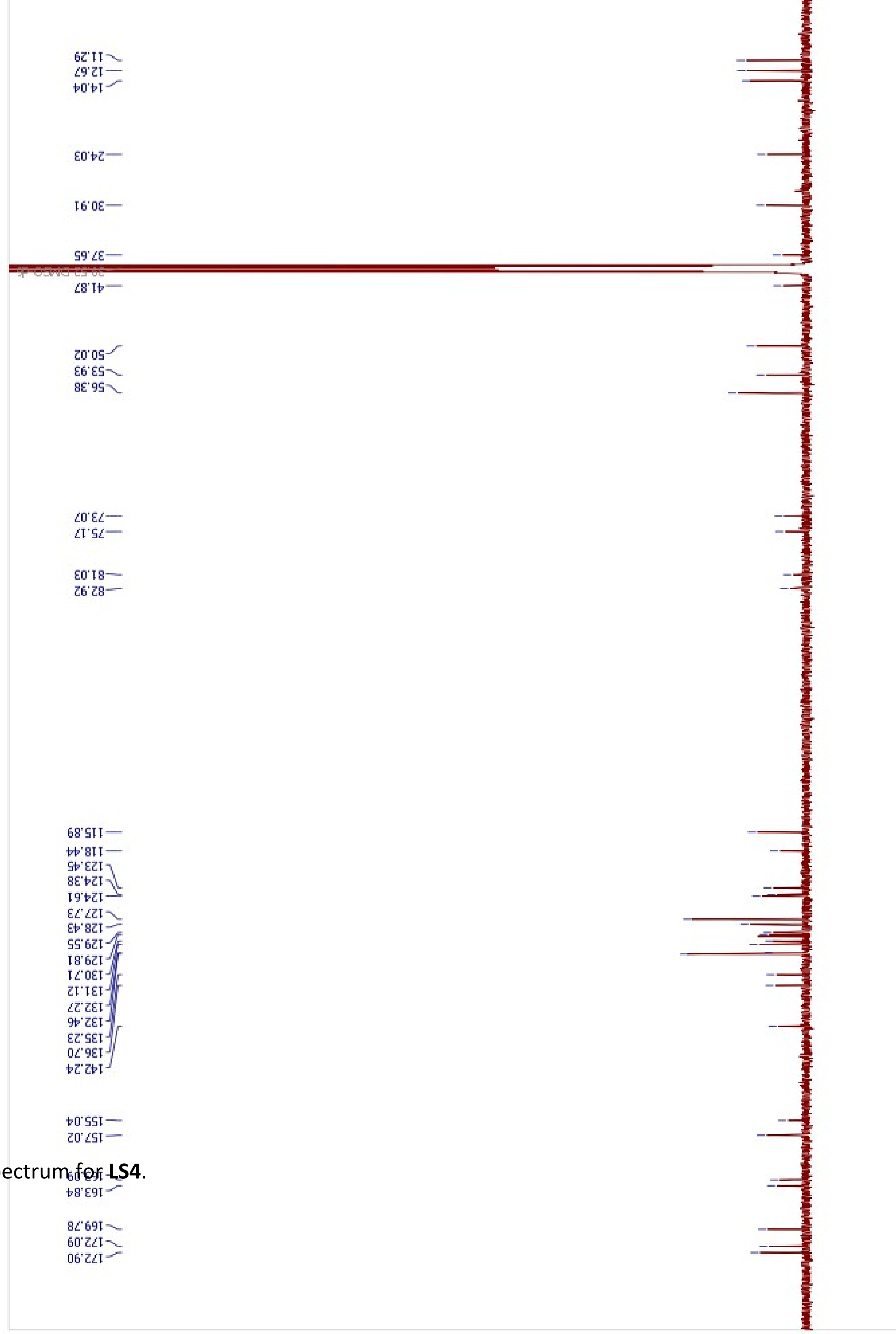

Figure S41. ^{13}C NMR spectrum for **LS4**.

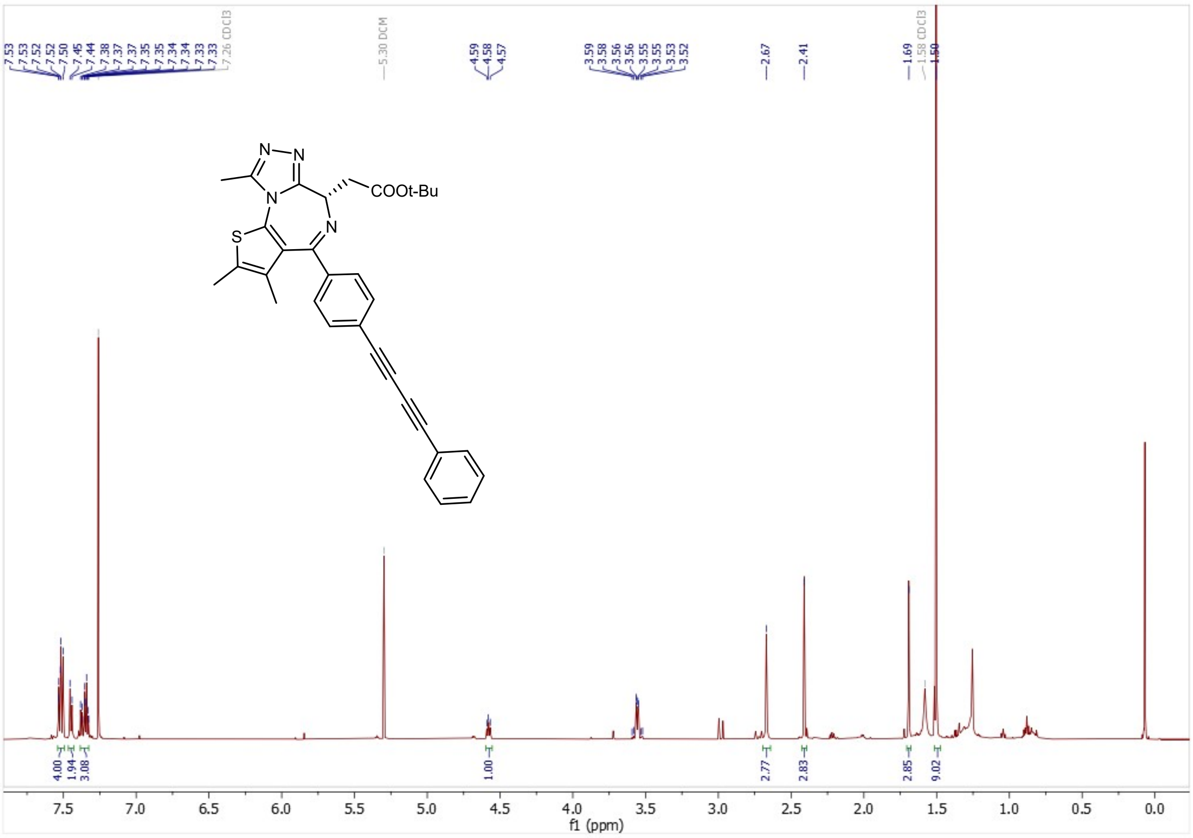

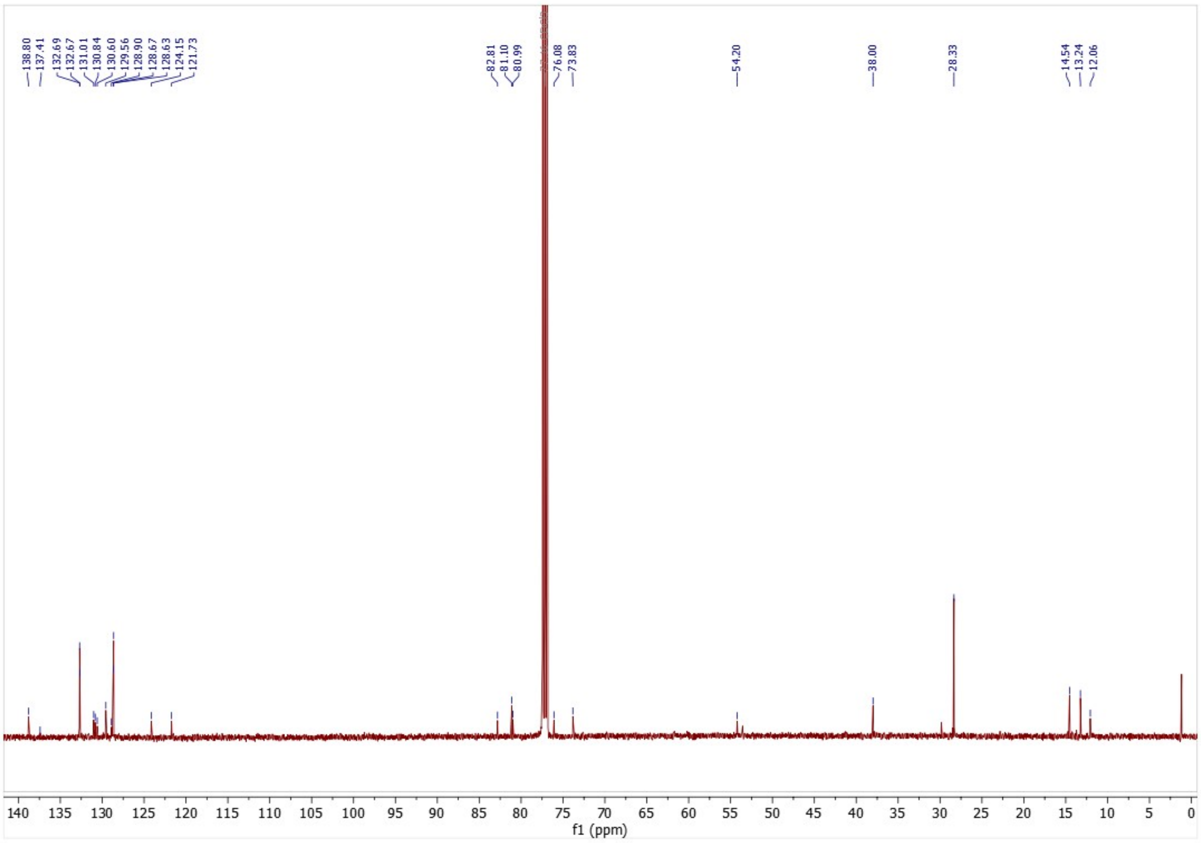

Figure S42. ^{1}H NMR (top) and ^{13}C NMR (bottom) spectra for **(+)-JQ1-*p*BADY**.

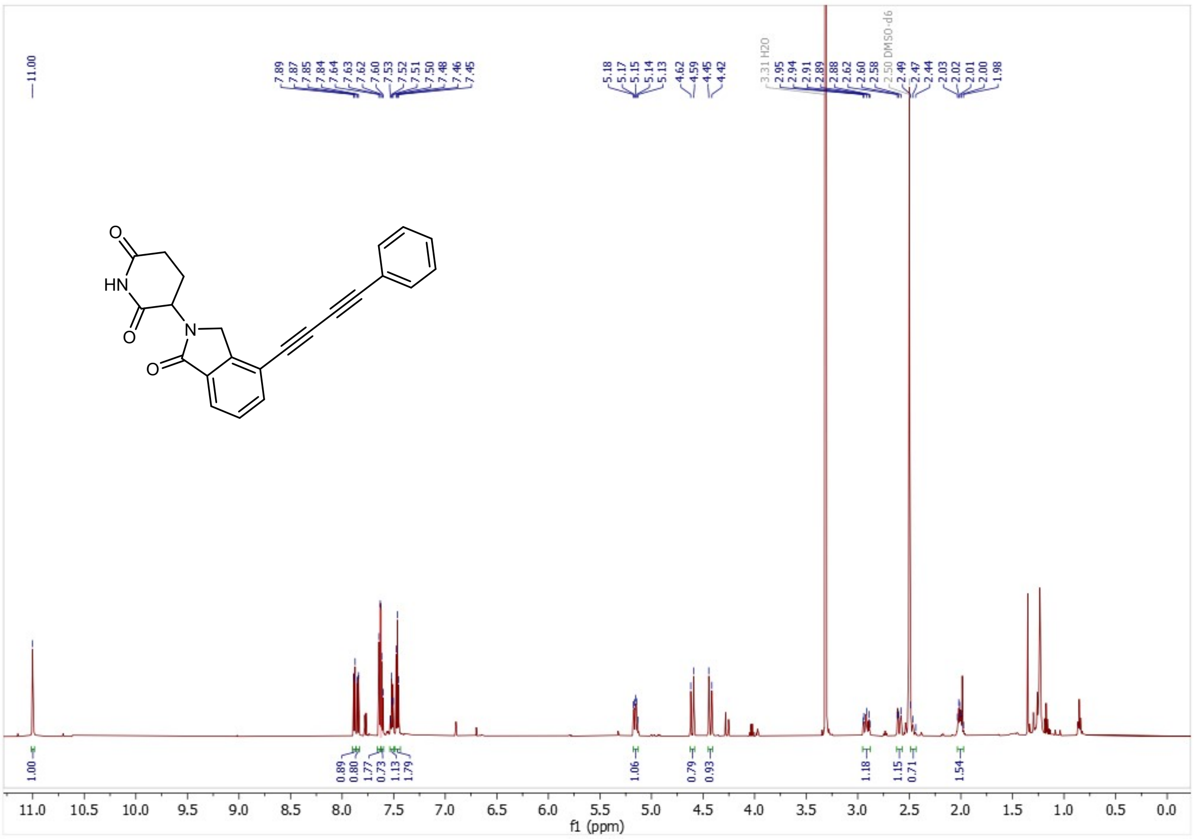

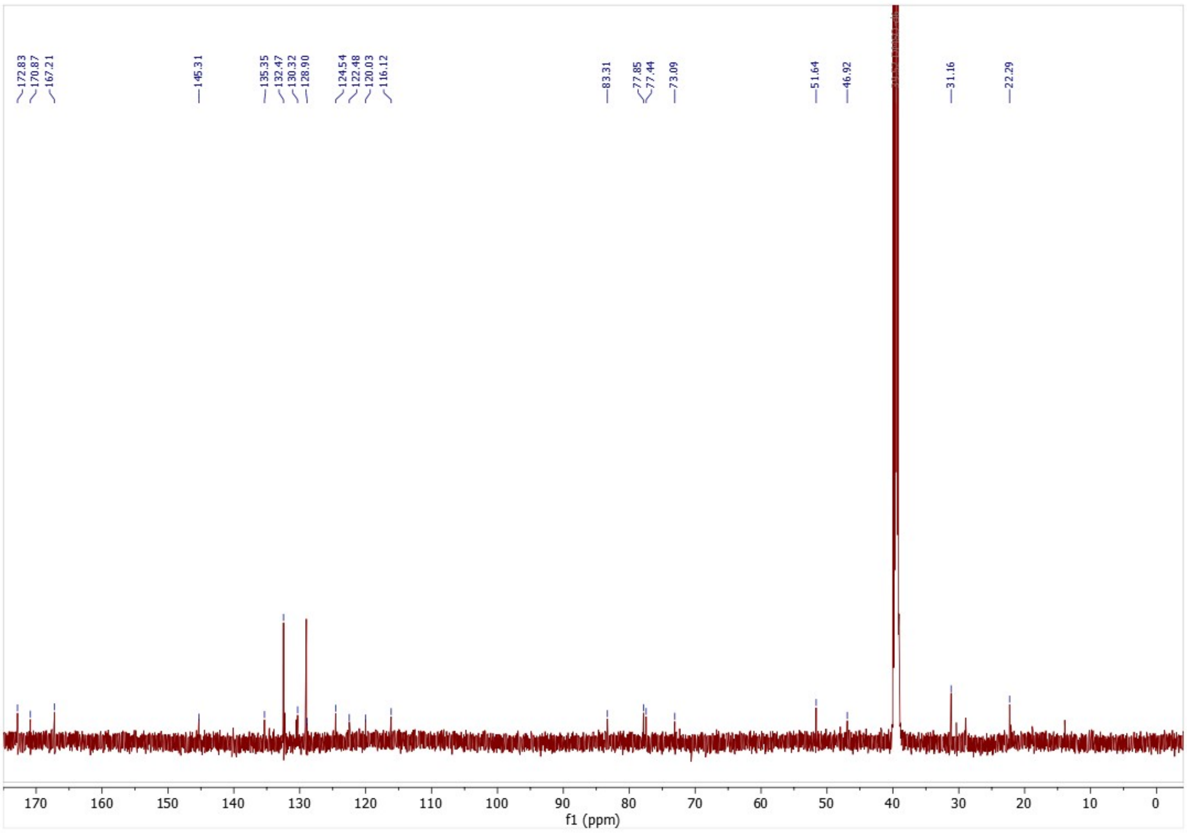

Figure S43. ^{1}H NMR (top) and ^{13}C NMR (bottom) spectra for **Lenalidomide-BADY**.
